# Intermolecular Sp^3^C─H Metalation of Non‐Nucleophilic Brønsted Bases Using Simple Lewis Acids

**DOI:** 10.1002/anie.202512254

**Published:** 2025-07-16

**Authors:** Anna V. Schellbach, Dominic R. Willcox, Miriana Guarnaccia, Gary S. Nichol, Valerio Fasano, Michael J. Ingleson

**Affiliations:** ^1^ EaStCHEM School of Chemistry University of Edinburgh Edinburgh EH9 3FJ UK; ^2^ Institute of Chemical Sciences Heriot‐Watt University Edinburgh EH14 4AS UK; ^3^ Department of Chemistry University of Milan Milan Via Golgi 19 Milan 20133 Italy

**Keywords:** Aluminium trichloride, C─H functionalisation, Frustrated Lewis Pairs, Lewis acids, Non‐nucleophilic bases

## Abstract

2,6‐Di‐*tert*‐butyl substituted pyridines (*^t^* Bu_2_‐py) are widely used non‐nucleophilic Brønsted bases. Their ubiquity is due to their highly hindered basic site and chemically robust nature. Herein we report that simple M_2_X_6_ Lewis acids (M═Al or Ga, X═Cl, Br or I) effect intermolecular sp^3^C─H metalation of *^t^* Bu_2_‐py bases under mild conditions. The sp^3^C─H metalated products can be converted in situ into ─BPin, ─iodo, ─bromo and ─hydroxy derivatives for further elaboration. Mechanistic investigations indicate that: i) a frustrated Lewis pair effects sp^3^C─H heterolysis to form the C─M bond and a protonated pyridine; ii) C─H metalation requires singly halide‐bridged super‐electrophilic M_2_X_6_ dimers for sufficiently low barriers. Finally, sp^3^C─H metalation using M_2_X_6_ is not limited to *^t^* Bu_2_‐py bases. Thus, it is important to be aware of this facile sp^3^C─H functionalisation when using a range of non‐nucleophilic Brønsted bases.

## Graphical abstract

Simple M_2_X_6_ Lewis acids (M═Al or Ga, X═Cl, Br or I) effect the sp^3^C─H metalation of a number of hindered bases, including the ubiquitous non‐nucleophilic 2,6‐ditertbutyl substituted pyridine/pyrimidines. Mechanistic studies indicate a frustrated Lewis pair mechanism requiring singly halide‐bridged super‐electrophilic M_2_X_6_ dimers for sufficiently low C─H metalation barriers.

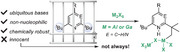

## Introduction

**Figure 1:**
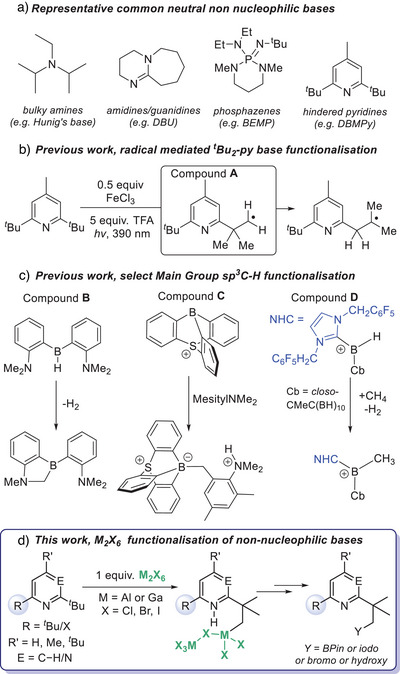
a) Select non‐nucleophilic bases. b) Radical‐based functionalisation of *^t^* Bu_2_‐py. c) Previous work on main group compounds effecting sp^3^C─H functionalisation. d) This work on sp^3^C─H functionalisation using just M_2_X_6_.

Sterically hindered Brønsted bases, termed non‐nucleophilic bases, are ubiquitous in chemistry. This is due to their ability to sequester protons while not (or only weakly) interacting with most other electrophiles. There are multiple classes of non‐nucleophilic bases, with the most established neutral examples being based on bulky amines (e.g., Hünig's base, Figure 1a),^[^ ^1^ ^]^ amidines/guanidines (e.g., DBU),^[^ ^2^, ^3^ ^]^ bulky phosphine derivatives (e.g., BEMP)^[^ ^2^ ^]^ and 2,6‐*^t^* Bu_2_‐pyridines (e.g., 2,6‐*^t^* Bu_2_‐4‐Me‐pyridine, DBMPy), with this family termed *^t^* Bu_2_‐py herein.^[^ ^4^, ^5^ ^]^ Of these bases, the most chemically robust are the *^t^* Bu_2_‐py class. While the utility of *^t^* Bu_2_‐py bases is long established,^[^ ^4^, ^5^ ^]^ they remain pervasive in the current literature (>450 references using them in the period 2020 to 2024).^[^ ^6^ ^]^ The robust nature of *^t^* Bu_2_‐py bases comes from their low energy HOMO, high aromatic stabilization energy, extreme bulk around N,^[^ ^7^ ^]^ and a periphery consisting of unactivated sp^3^C─H bonds. The functionalisation of the latter is a significant challenge,^[^ ^8^, ^9^, ^10^, ^11^, ^12^, ^13^ ^]^ and to our knowledge, there is only one report on the C─H functionalisation of a *^t^* Bu unit in a *^t^* Bu_2_‐py base.^[^ ^14^ ^]^ This required high energy radicals generated by photolysis that abstract H^•^ from protonated DBMPy to form primary radical **A** (Figure 1b). **A** rearranges to a more stable 3° radical before reacting further. The identification of another sp^3^C─H functionalisation of *^t^* Bu_2_‐py bases would be notable, particularly if the transformation proceeds using simple commodity reagents under mild conditions.

Conceptually, if a strong Lewis acid (LA) interacts with one of the *^t^* Bu sp^3^C─H σ bonds in *^t^* Bu_2_‐py this could lower the pK_a_ sufficiently to enable deprotonation by the proximal pyridine and concomitant C─(LA) bond formation.^[^ ^15^ ^]^ This would represent an intermolecular frustrated Lewis pair (FLP) type C─H cleavage process as a Lewis acid and a Brønsted base are both required (i.e., strong pyridyl coordination to the Lewis acid needs to be disfavoured). While FLP functionalisation of sp and sp^2^C─H bonds is now well‐documented,^[^ ^16^, ^17^, ^18^, ^19^ ^]^ to date FLPs that cleave sp^3^C─H bonds are rare.^[^ ^20^, ^21^ ^]^ The examples reported to date have the Brønsted base, Lewis acid and sp^3^C─H unit all preorganized in one molecule (e.g., compound **B**, Figure 1c),^[^ ^22^ ^]^ involve relatively complex strong Lewis acids (e.g., compounds **C** and **D**)^[^ ^23^, ^24^ ^]^ or use toxic heavy metals (e.g., Tl(O_2_CCF_3_)_3_).^[^ ^25^ ^]^ It would be notable if FLP‐type functionalisation of unactivated sp^3^C─H bonds could be achieved using simple main group Lewis acids such as aluminium trichloride. This would represent the first catalyst free homogenous alumination of an unactivated sp^3^C‐H bond.^[^ ^26^, ^27^ ^]^

Herein we report that *^t^* Bu_2_‐py bases undergo sp^3^C─H metalation using M_2_X_6_ Lewis acids (M═Al or Ga, X═Cl, Br or I) under mild conditions. Furthermore, we show that intermolecular sp^3^C─H functionalisation using M_2_X_6_ proceeds with other less hindered Brønsted bases and that the sp^3^C─M products (e.g., compound **1**) can be converted into useful bench stable compounds.

## Results and Discussion

**Scheme 1:**
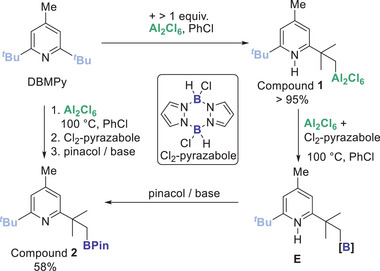
The formation of compounds **1** and **2**. Yields by NMR spectroscopy versus an internal standard.

During our studies using pyrazabole derivatives in electrophilic borylation,^[^ ^28^, ^29^ ^]^ it was observed that heating dichloro‐pyrazabole, Al_2_Cl_6_, DBMPy and activated arenes in chlorobenzene (PhCl) led to a new lower symmetry DBMPy species. This was identified as an sp^3^C─H functionalised product by conversion to the sp^3^C─BPin species, compound **2** (Scheme 1). Repeating this reaction in the absence of an activated arene led to the formation of **2** as the major product (by NMR spectroscopy). Compound **2** displays the expected NMR resonances, including a *δ*_11B_ = 33.5 and aliphatic ^1^H resonances in a 3:9:6:2 ratio. Given the widespread use of *^t^* Bu_2_‐py bases, including in S_E_Ar reactions mediated by Al_2_Cl_6_,^[^ ^30^ ^]^ it was assessed if a boron electrophile was required for sp^3^C─H functionalisation. In the absence of dichloro‐pyrazabole sp^3^C─H functionalisation still occurred, with an effectively quantitative (>95%, Scheme 1) conversion when using ≥1 equiv. of Al_2_Cl_6_. When less than one equiv. of Al_2_Cl_6_ was used, unreacted DBMPy remained. Optimal outcomes were observed using a small excess of Al_2_Cl_6_ (e.g., 1.25 equiv.). The in situ ^1^H NMR spectrum of the sp^3^C‐H functionalised product displayed a N─ *H* resonance (broad at 10.5 ppm), and aliphatic resonances in a 3:6:9:2 ratio. These observations combined with subsequent calculations (vide infra) led us to assign the C─H metalated product as compound **1** (Scheme 1). This formulation was supported by analysis in CDCl_3_ (which revealed two inequivalent *meta* Aryl─ *H* resonances). Notably, the addition of dichloro‐pyrazabole/Al_2_Cl_6_ to **1** led to transmetalation and formation of species assigned as **E** (Scheme 1). **E** is not well‐defined as it is a range of pyrazaboles varying in the exocyclic groups, but these convert into **2** on in situ pinacol protection. This suggests the observed sp^3^C─H borylation proceeds via initial sp^3^C─H alumination, a hypothesis supported by attempts to borylate DBMPy with pyrazabole activated by [Ph_3_C][B(C_6_F_5_)_4_] leading to no borylated product post pinacol protection.

**Scheme 2:**
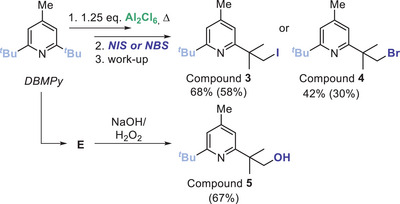
One‐pot alumination/halogenation, and one‐pot alumination / transmetalation / oxidation of DBMPy. Yields versus an internal standard with isolated yields in parentheses.

With the functionalisation of DBMPy using Al_2_Cl_6_ established the conditions when this occurs were explored. This revealed that formation of **1** occurs even at ambient temperature (albeit slowly), and at 80 °C in PhCl > 95% conversion to **1** takes only 2 h despite the incomplete dissolution of Al_2_Cl_6_ under these conditions. Given the sensitivity of **1** and **2** to protodemetalation during purification, the in situ conversion of **1** into more stable derivatives and potentially useful (e.g., for subsequent derivatisation) was explored. This was achieved by the reaction of crude **1** with *N*‐iodo‐succinimide (NIS) at room temperature for 18 h. This enabled isolation of **3** in 58% yield (Scheme 2). Note, under these conditions DBMPy does not react with NIS, even in the presence of Al_2_Cl_6_,^[^ ^31^ ^]^ indicating initial C─H alumination is essential for accessing **3**. NBS also reacted analogously affording **4**, albeit in a lower yield than when using NIS. The mass balance in these reactions was free DBMPy which is reformed to some extent on addition of NIS/NBS. Attempts to use **1** to alkylate benzophenone were unsuccessful, with this also resulting in the formation of DBMPy, in this case the Ph_2_C═O→AlCl_3_ adduct was identified as the by‐product.^[^ ^32^ ^]^ Thus, the addition of carbonyl compounds to **1** can lead to protodemetalation of the sp^3^C─Al unit by the N─H unit leading back to DBMPy. Another sp^3^C‐functionalised product is accessible post transmetalation by oxidation of **E** to form alcohol **5** in 67% isolated yield (Scheme 2).

**Figure 2:**
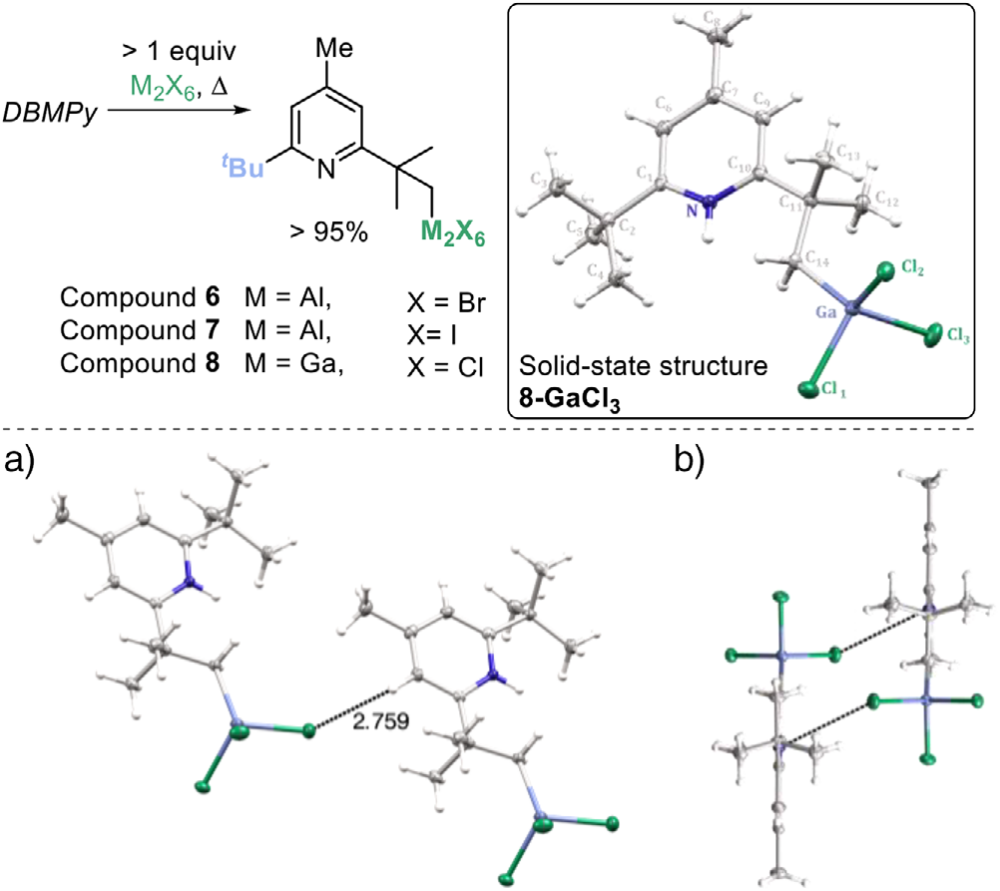
Top, the formation of compounds **6**–**8**. Top right, solid‐state structure of **8‐GaCl_3_**, ellipsoids at 50% probability. Bottom, the extended structure of **8‐GaCl_3_** showing select intermolecular interactions as black dashed lines.

Next, we explored what other Lewis acids effected sp^3^C─H metalation of DBMPy. The heavier halide congeners, AlBr_3_ and AlI_3_ both perform C─H metalation to form **6** and **7**, respectively (Figure 2, top). These reactions proceed more rapidly compared to those using Al_2_Cl_6_, presumably in part due to the improved solubility of these Lewis acids in PhCl. Note, ≥ 1 equiv. of Al_2_X_6_ is required in each case for full consumption of DBMPy. In contrast to Al_2_X_6_, Al_2_Me_6_, Al_2_Et_6_, Al_2_Me_4_Cl_2_, and ethyl aluminium sesquichloride each resulted in no C─H metalation even after heating in PhCl (by in situ NMR spectroscopy). Metalation is not limited to Al_2_X_6_, as Ga_2_Cl_6_ effects C─H metalation to produce **8** (Figure 2 top). However, other Group 13 Lewis acids, specifically BCl_3_, BBr_3_, BI_3_, InCl_3_, and InBr_3_ all led to no C─H functionalisation (by in situ NMR spectroscopy). A number of other metal halides also resulted in no observable sp^3^C─H metalation (TiCl_4_, ZrCl_4_, SiCl_4_, PCl_5_, and ZnCl_2_). While limited to Al/Ga Lewis acids this is still notable as to our knowledge this is the first homogenous alumination/galliation of unactivated sp^3^C─H bonds.

On standing, crystals suitable for single crystal X‐ray diffraction analysis slowly (over weeks) formed from a reaction of Ga_2_Cl_6_ and DBMPy. This confirmed formation of an sp^3^C─H metalated product, specifically **8‐GaCl_3_**.^[^ ^33^ ^]^ This zwitterion contains one four coordinate anionic gallium unit and a protonated pyridine (inset Figure 2). The structure of **8‐GaCl_3_** contains a Ga‐C bond length (1.980(2) Å) comparable to that in other [RGaCl_3_]^−^ anions.^[^ ^34^, ^35^ ^]^ However, there is evidence for steric induced distortions in **8‐GaCl_3_**. This includes a large C─C─Ga angle (120.22(15)°) and the two eclipsed (with the proximal methyls) chlorides being angled away from the quaternary carbon (C─Ga─Cl_eclipsed_ = 117.28(7) and 118.31(7), whereas C─Ga─Cl_non‐eclipsed_ = 106.14(7)). The extended structure of **8‐GaCl_3_** contains multiple Ga─Cl⋯H─C interactions (see Figure 2a) with the shortest being to a *meta*‐arylC─H (at 2.7591(6) Å). In contrast, the shortest intermolecular Cl⋯H─N contact (e.g., Figure 2b) is significantly longer (>3.2 Å), consistent with considerable steric shielding of the N─H unit by the two flanking *^t^* Bu substituents.

**Scheme 3:**
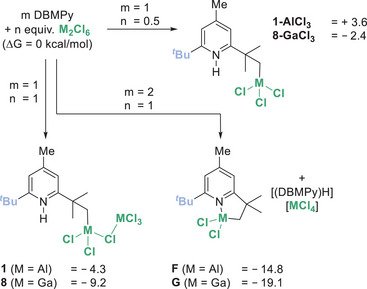
Calculated free energies (kcal mol^−1^) of C─H metalation products.

While confirming C─H functionalisation the structure of **8‐GaCl_3_** is not consistent with the stoichiometry required for complete consumption of DBMPy (≥ 1 equiv. of Ga_2_Cl_6_ is required, Figure S100). This suggested that **8‐GaCl_3_** is not the major product in solution, but instead forms on standing (over weeks) due to it precipitating as a crystalline solid. To assess this hypothesis the thermodynamics of the C─H metalation reactions using Al_2_Cl_6_ and Ga_2_Cl_6_ were calculated at the MN15/Def2TSVPP (SMD = chlorobenzene) level (Scheme 3); note all calculations herein are performed at this level. These calculations indicated that the bimetallic C─M_2_X_6_ products (e.g., **1**/**8**) are thermodynamically favored over the formation of the mono‐metallic derivatives **1‐AlCl_3_** and **8‐GaCl_3_**. While the formation of **8‐GaCl_3_** is also exergonic from DBMPy and 0.5 equiv. Ga_2_Cl_6_, the formation of **1‐AlCl_3_** is endergonic. The latter is consistent with the outcome from the reaction of **1** and benzophenone, which react to form Ph_2_C═O→AlCl_3_ and presumably mono‐metallic **1‐AlCl_3_**. **1‐AlCl_3_** then would convert to DBMPy (which is observed by NMR spectroscopy) in line with the relative calculated energies. This highlights the importance of the bimetallic unit in **1** for providing an exergonic metalation process when using Al_2_Cl_6_.

Notably, the formation of the cyclometalated compounds **F**/**G** and an equiv. of [(DBMPy)H][MCl_4_] was found to be favoured thermodynamically over the formation of **1/8**. However, **F/G** are not observed in any reaction mixtures using DBMPy, nor is any significant amount of [(DBMPy)H]^+^ observed (< 5% of [(DBMPy)H]^+^ is observed which is attributed to trapping of Brønsted acids generated by reaction of M_2_X_6_ with trace protic impurities). Furthermore, combining **1/8** with free DBMPy does not lead to formation of **F/G** and [(DBMPy)H][MCl_4_]. This indicates a significant kinetic barrier to the interconversion of **1**(**8**) and **F**(**G**), presumably due to the steric bulk surrounding the N─H unit in **1/8** which prevents proton transfer to free DBMPy. These observations preclude the intermediacy of **F/G** in these metalation reactions and thus disfavours a N‐directed C─H metalation mechanism (involving initial _py_N→M_x_Cl_3x_ Lewis adduct formation and metalation then proceeding via the cation [(DBMPy)MCl_2_]^+^) as this would be expected to form **F/G** as metalation products.

Attention thus turned to identifying a feasible mechanism for the C─H metalation of DBMPy. First, C─H metalation to form **1** proceeds analogously when performed in the dark. This, coupled with the absence of any rearrangement products (as observed when radical **A** is an intermediate), and there being no activation of the Aryl‐*CH _3_* unit (which contain weaker C─H bonds relative to the *^t^* Bu C‐H bonds), disfavours a radical mechanism. Analysis of the Lewis acids effective for metalation of DBMPy reveals some correlation with Lewis acidity, with only the most Lewis acidic aluminium compounds resulting in C─H functionalisation.^[^ ^36^, ^37^ ^]^ However, it is significant that BX_3_ Lewis acids do not effect DBMPy sp^3^C─H functionalisation despite BX_3_ Lewis acids having comparable (or greater) calculated Lewis acidity toward “soft” nucleophiles″ (e.g., hydride/methide) relative to monomeric MX_3_ (M═Ga/Al) species.^[^ ^36^ ^]^ The major difference between these boron and aluminium/gallium Lewis acids is that boron trihalides exist as monomers in solution, while the latter exist as Al_2_X_6_/Ga_2_X_6_ dimers in weakly coordinating solvents (such as PhCl). The disparate outcomes observed using dimeric M_2_X_6_ Lewis acids versus BX_3_ could be kinetic and/or thermodynamic in origin, with the latter consistent with the binding of chloride to form bimetallic [M_2_Cl_7_]^−^ anions calculated to be more thermodynamically favored than the binding of chloride to form monometallic anions [MCl_4_]^−^;^[^ ^38^ ^]^ while the former would be consistent with carbonyl‐olefin metathesis reactions having lower barriers when using M_2_X_6_ dimeric Lewis acids compared to that catalyzed with MX_3_.^[^ ^39^ ^]^ This was attributed to the greater Lewis acidity of mono‐halide bridged M_2_X_6_ Lewis acids relative to MX_3_. To provide more insight into the disparate reactivity we explored computationally the sp^3^C─H metalation of DBMPy using dimeric M_2_Cl_6_ (M═Al/Ga) and BCl_3_.

**Figure 3:**
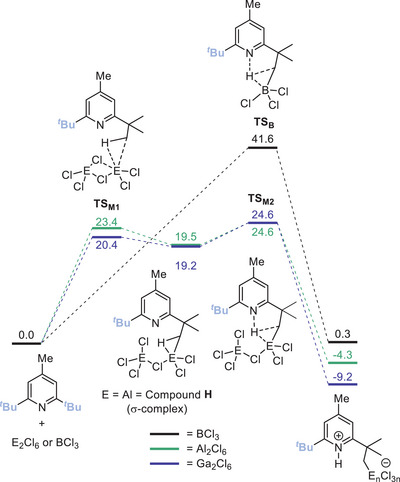
Calculated sp^3^C─H metalation mechanism for BCl_3_, Al_2_Cl_6_ and Ga_2_Cl_6_ (free energies in kcal mol^−1^).

A mechanism was identified (Figure 3) that proceeds via opening of one μ‐Cl to form σ‐complex **H** (at Δ*G* + 19.5 kcal mol^−1^), via a feasible barrier (**TS_Al1_** = +23.4 kcal mol^−1^). The reaction then proceeds by a concerted metalation/deprotonation process with a barrier of +24.6 kcal mol^−1^ (**TS_Al2_**). The structure of **H** contains a single μ─Cl and an elongated Al⋯ּAl distance relative to that in Al_2_Cl_6_ (3.88 Å in **H** versus 3.20 Å calculated for Al_2_Cl_6_). A significant interaction between a *^t^* Bu C─H and the proximal Al centre in **H** was indicated by the compressed ΣCl─Al─Cl angles of 340.1° (relative to 360° for a trigonal planar Al) and a short Al⋯H_C_ distance of 1.96 Å. The C─H unit interacting with the aluminium centre in **H** is preorganized for deprotonation, with a short N⋯H distance (N⋯H = 1.989 Å). An analogous mechanism was calculated for Ga_2_Cl_6_ with comparable intermediate / transition states (with the highest barrier **TS_Ga2_** = +24.6 kcal mol^−1^). It is notable that while homogenous alumination/galliation of unactivated sp^3^C─H bonds has not been previously reported to our knowledge, alkane C─H cleavage has been reported using heterogeneous systems, e.g., alumina and Ga‐doped zeolites,^[^ ^40^, ^41^ ^]^ albeit at much higher temperatures than required to form **1/8**. While these heterogeneous systems involve ill‐defined Al/Ga based Lewis acids, multiple calculations support an ambiphilic mechanism involving a strongly Lewis acidic Al/Ga centre and a proximal oxygen‐basic site that combined effect C─H heterolysis.^[^ ^40^, ^41^ ^]^ Thus, the homogeneous sp^3^C─H metalations of DBMPy using M_2_X_6_ represent well‐defined model systems for heterogeneous Al/Ga mediated alkane activation processes.

Moving to calculations on sp^3^C─H functionalisation using BCl_3_, the overall reaction is less energetically favoured than the Al and Ga analogues, being effectively thermoneutral (Figure 3). Furthermore, the calculated mechanism is different with no σ‐complex analogous to **H** found. Instead sp^3^C─H metalation using BCl_3_ has a single transition state involving concerted cleavage of the C─H bond by BCl_3_ and the pyridyl lone pair. Notably, this barrier is much higher (**TS_B_** = +41.6 kcal mol^−1^) than those involving Al_2_Cl_6_/Ga_2_Cl_6_. Thus, there is a dramatic difference when singly bridged bimetallic M_2_Cl_6_ complexes are involved in sp^3^C─H cleavage. We attribute this to the greater Lewis acidity of the mono bridged Cl_3_M─(μ─Cl)─MCl_2_ species. This is consistent with Schlinder's studies on carbonyl‐olefin metathesis,^[^ ^39^ ^]^ and with alkane activation using heavy main group complexes where more electrophilic complexes have lower barriers to sp^3^C─H metalation (specifically relative barriers are Pb(IV) < Tl(III) < Hg(II) for the M(O_2_CCF_3_)_x_ series).^[^ ^25^ ^]^

**Figure 4:**
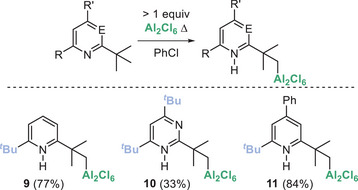
Other *^t^* Bu_2_‐py bases amenable to sp^3^C─H alumination. Yields by NMR spectroscopy versus an internal standard.

With a plausible mechanism identified, our attention turned to identifying other bases that undergo this C─H metalation. Firstly, another widely used non‐nucleophilic base 2,6‐di‐tert‐butyl‐pyridine was found to undergo alumination with Al_2_Cl_6_. Based on the DBMPy studies we assign the product as **9** which is formed in 77% yield by in situ NMR spectroscopy (Figure 4). Note, in situ monitoring is facilitated by the C─H aluminated products containing a diagnostic C *H*_2_─Al resonance (see Supporting Information). For this substrate, sp^3^C─H functionalisation was confirmed by reaction of **9** with NIS to form the *para*‐H analogue of **3** (termed **3‐*p*‐H**) in 45% isolated yield. Another common non‐nucleophilic base, 2,4,6‐tri‐*^t^* Bu‐pyrimidine, also reacted with Al_2_Cl_6_ by *^t^* Bu sp^3^C─H metalation which proceeded in 33% yield after 18 h at 120 °C. C─H alumination was confirmed again by conversion to the sp^3^C─iodinated pyrimidine product. This pyrimidine undergoes a selective single C─H alumination (for the *^t^* Bu group located between both N atoms), with no double C─H alumination observed. The absence of a second C─H alumination is presumably due to the first C─H cleavage quaternising one nitrogen thus reducing the Brønsted basicity of the second N center. The aluminated product is assigned as compound **10** by analogy to the DBMPy alumination product. Incorporating a *para* phenyl resulted in selective sp^3^C─H alumination (to form **11**), with no sp^2^C─H alumination of the phenyl unit observed.

**Scheme 4:**
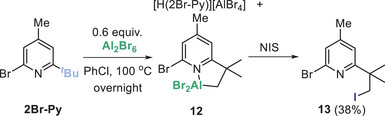
sp^3^C─H alumination of 2Br─Py using Al_2_Br_6_. Yield of isolated compound **13** (relative to **12**).

Reducing the *ortho* steric bulk by using 2,6‐diisopropyl‐pyridine resulted in no C─H metalation using Al_2_Cl_6_ under a range of conditions. In contrast, 2‐bromo‐6‐*^t^* Bu‐pyridine (termed 2Br‐Py herein) was amenable to C─H alumination (using Al_2_Br_6_ to preclude formation of mixtures from C─X/Al─X halide scrambling).^[^ ^42^ ^]^ Under a range of conditions, the maximum C─H alumination yield (versus an internal standard) for 2Br‐Py was ca. 45%. In addition, ca. 55% protonated 2Br‐Py was formed. In this case, the lower bulk around the nitrogen centre in 2Br─Py, relative to that in DBMPy, is expected to result in lower barriers for proton transfer steps for 2Br‐Py derivatives. Thus, formation of the 2Br‐Py analogue of **F** (Scheme 3) is feasible, which would lead to compound **12** (Scheme 4) and [H(2Br‐Py)]^+^ in a 1:1 ratio (the theoretical maximum yield of **12** of 50% is not achieved due to the presence of trace protic species which leads to formation of an additional ca. 5% of [H(2Br‐Py)]^+^). Attempts to crystallize **12** to confirm a cyclo‐metalated structure were not successful. However, the in situ NMR data is consistent with this outcome, furthermore **12** is soluble in pentane (in contrast to **1**) and only 0.6 equiv. of Al_2_Br_6_ is required for full consumption of 2Br‐Py (as **12** is a mono‐metallic species in contrast to bimetallic **1**). While formulation as cyclo‐metalated **12** is tentative, C─H alumination was confirmed by reaction of **12** with NIS to form **13**. Compound **13** is bench stable enabling isolation and full characterization.

**Scheme 5:**
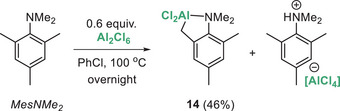
C─H alumination of MesNMe_2_ using Al_2_Cl_6_. Yield by NMR spectroscopy versus an internal standard.

With a range of *ortho*‐*^t^* Bu‐substituted pyridine bases found to react with Al_2_X_6_ other hindered nitrogen bases were tested. When Hünig's base was combined with Al_2_Cl_6_ no C─H alumination was observed even on prolonged heating. We surmised that this was due to the shorter hydrocarbyl unit in Hünig's base relative to that in ^t^Bu_2_‐py bases, resulting in a more strained (and thus higher energy) transition state for sp^3^C─H cleavage. To test this hypothesis an alternative hindered tertiary amine was selected, specifically, MesNMe_2_ as this amine contains a longer hydrocarbyl chain (N─C_sp2_─C_sp2_─C_sp3_) and undergoes C─H cleavage using bora‐triptycene **C**.^[^ ^24^ ^]^ Notably, combination of MesNMe_2_ with Al_2_Cl_6_ led to ca. 46% sp^3^C─H alumination (by in situ NMR spectroscopy) with the mass balance being [MesN(H)Me_2_][Al_x_Cl_3x+1_]. Consistent with this outcome, only 0.6 equiv. of Al_2_Cl_6_ was required to fully consume MesNMe_2_. By analogy to **12** we assign the C─H alumination product derived from MesNMe_2_ as the cyclo‐metalated compound **14** (Scheme 5). This demonstrates that sp^3^C─H alumination using just Al_2_Cl_6_ is not limited to *ortho*‐*^t^* Bu‐pyridine bases.

**Scheme 6:**
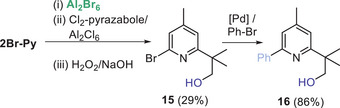
Sequential functionalisation of 2Br─Py to form **15** and **16**. Isolated yields shown.

Finally, as pyridine units are privileged moieties in active pharmaceutical ingredients the C─H alumination of 2Br‐Py was used to rapidly access functionalized pyridines (Scheme 6). Specifically, 2Br─Py underwent a sequence of alumination / transmetalation / oxidation in one‐pot to form compound **15** in 29% overall yield (note this is a reasonable yield given the multiple steps involved and the fact that only 50% of 2Br─Py can undergo C─H alumination). Subsequent cross coupling of **15** was then straight forward, as exemplified by formation of **16** under standard Suzuki‐Miyaura coupling conditions. This sequence provides an alternative to Minisci chemistry to access these products, which is notable as Minisci‐type hydroxy alkylation of pyridines often gives mixtures of C2/C4 hydroxy‐alkylated products.^[^ ^43^ ^]^ In contrast only formation of the C2 *^t^* Bu─OH group occurs via this C─H alumination process.

## Conclusion

The ubiquitous class of non‐nucleophilic Brønsted bases based on 2,6‐di‐tert butyl pyridines/pyrimidines undergo facile sp^3^C─H functionalisation. This intermolecular sp^3^C─H functionalisation occurs with simple aluminium and gallium M_2_X_6_ Lewis acids and proceeds even at room temperature. Thus, it is surprising that this transformation has gone unnoticed to date. Mechanistic studies indicate that dimeric Lewis acids are essential for sp^3^C‐H metalation, an observation attributed to the extreme Lewis acidity of mono‐halide bridged M_2_X_6_ units which is key to enable a sufficiently low barrier concerted C‐H bond heterolysis. The sp^3^C‐aluminated products can be converted into a range of useful derivatives, specifically: sp^3^C─BPin, ‐halogenated and ‐hydroxylated, thus this transformation represents a novel route to access functionalised pyridines. Furthermore, this transformation is not limited to extremely hindered pyridines, with a 2‐bromo‐pyridyl derivative and a hindered tertiary amine, Mesityl‐NMe_2_, also undergoing intermolecular sp^3^C─H alumination. Given the widespread use of these hindered bases combined with the facile nature of this sp^3^C─H functionalisation, it is important to be aware of this transformation when considering using these non‐nucleophilic bases.

## Conflict of Interests

The authors declare no conflict of interest.

## Supporting information



Supporting Information

Supporting Information

Supporting Information

## Data Availability

The data that support the findings of this study are available in the Supporting Information of this article.
