# Supplementary material for: Intermolecular Sp3C─H Metalation of Non‐Nucleophilic Brønsted Bases Using Simple Lewis Acids
Source: Angew Chem Int Ed Engl. 2025 Jul 16;64(35):e202512254. doi: 10.1002/anie.202512254 (PMC12377440; doi:10.1002/anie.202512254)
Supplement: Supplementary file 1 — Supporting Information [file ANIE-64-e202512254-s003.pdf]

Supporting Information

Intermolecular sp^3C-H Metalation of Non-Nucleophilic Brønsted Bases Using Simple Lewis Acids

Anna V. Schellbach, Dominic R. Willcox, Miriana Guarnaccia, Gary S. Nichol, Valerio Fasano and Michael J. Ingleson*

School of Chemistry, University of Edinburgh, Edinburgh, EH9 3 FJ, UK

Table of Contents

1. General Considerations 4
2. C(sp^3)−H metalation 6
   2.1 General Procedure 1 6
   2.2 Lewis Acid Scope and Characterisation of Metallated DBMPy products from using MX_3 (Compounds 1-8) 6
   2.3. DBMPy functionalisation with $AlCl_3$ (1) 7
   2.4. DBMPy functionalisation with $AlBr_3$ (6) 7
   2.5. DBMPy functionalisation with AlI_3 (7) 8
   2.6. DBMPy functionalisation with $GaCl_3$ (8) 9
3. NMR Spectra of Metalated DBMPy 10
4. Synthesis and Activation of Pyrazaboles 24
   4.1. Synthesis of pyrazabole 24
   4.2 Synthesis of dichloropyrazabole 24
5. NMR Spectra of Synthesised Pyrazabole species 26
6. Functionalisation of metalated DBMPy 28
   6.1. General Procedure 2 – Transmetallation using a Pyrazabole Derivative 28
   6.2. Synthesis of the C(sp^3)−H borylated, pinacol protected DBMPy (2) 28
   6.3. Synthesis of the C(sp^3)−H iodinated DBMPy (3) 29
   6.4. Synthesis of the C(sp^3)−H brominated DBMPy (4) 30
   6.5. Synthesis of the C(sp^3)−H functionalised, oxidised DBMPy (5) 30
7. NMR Spectra of functionalised DBMPy 32
8. Alumination of other substrates 39
   8.1. Synthesis of the C(sp^3)−H aluminated and iodinated (9) 39
   8.2. Synthesis of the C(sp^3)−H aluminated (10) 40
   8.5. Synthesis of the C(sp^3)−H aluminated and iodinated (11) 41
   8.3. Synthesis of the C(sp^3)−H aluminated (12) 42
   8.1. Synthesis of the C(sp^3)−H aluminated (14) 42
9. NMR Spectra of other aluminated Substrates 44
10. Synthesis of C(sp^3)−H functionalised 2Br-py 71
   10.1. Synthesis of 2-bromo-4-methyl-6-*t*Bu-pyridine (2Br-Py) 71
   10.2. Synthesis of (13) 71
   10.3. Synthesis of (15) 72
   10.4. Synthesis of (16) 73
11. NMR Spectra of the C(sp^3)−H functionalised 2Br-Py 74
12. Mechanistic Studies 78
   11.1. Effect of 1.2 or 2.5 equiv. of MX_3 on metallation of pyridine 78

11.2. Reaction of 2,6-*t*Bu-4-methyl-pyridine with 2.5 equiv. $GaCl_3$ over time 79
11.3. *In-situ* NMR for the alumination of $MesNMe_2$ 80
11.3. Attempt at *in-situ* alkylation of benzophenone using compound 1 82
13. Computational Data 83
13.1. Computational Details 83
14. Crystallographic Data 84
14.1. Crystal structure of compound 8-$GaCl_3$ 84
15. References 86

1. General Considerations

All reactions were performed under inert conditions using standard Schlenk techniques or in an *MBraun Unilab* glovebox (< 0.1 ppm H_2O / O_2).

Unless otherwise stated, solvents were degassed with nitrogen, dried over activated aluminium oxide (Solvent Purification System: *Inert PureSolv MD5 SPS*) and stored over 3 Å molecular sieves in ampules equipped with J. Young's valves. Chlorobenzene, 1,2-difluorobenzene and 1,2-dichlorobenzene were dried over calcium hydride, distilled and stored over 3 Å molecular sieves. Deuterated solvents ($CDCl_3$ (99.6% D, Sigma Aldrich)) were stored over 3 Å molecular sieves. All chemicals were, unless stated otherwise, purchased from commercial sources and used as received. BCl_3 (1M in DCM) was transferred to an ampule fitted with a Young's Tap prior to use.

Column chromatography was performed on a *Teledyne Isco CombiFlash® 100* instrument using *Advion-Interchim* columns (spherical silica, 25 µm).

NMR spectra (^{1}H, ^{1}H{^{11}B}, ^{11}B, ^{11}B{^{1}H}, ^{13}C{^{1}H} and ^{27}Al) were recorded on *Bruker Avance III 400 MHz*, *Bruker Avance III 500 MHz or Bruker PRO 500 MHz* spectrometers. Chemical shifts (δ) are quoted in parts per million (ppm), coupling constants (J) are given in hertz (Hz) to the nearest 0.5 Hz, and as positive vales regardless of their real individual signs. ^{1}H and ^{13}C shifts are referenced to the appropriate residual solvent peak while ^{11}B and ^{27}Al are referenced relative to external $BF_3{\cdot}Et_2O$ and $Al(NO_3)_3$, respectively. Abbreviations used are s (singlet), d (doublet), t (triplet), q (quartet), p (pentet), dd (doublet of doublets), dt (doublet of triplets), m (multiplet), br (broad). ^{13}C resonances of carbon atoms directly bonded to boron or aluminium atoms were usually observed as broadened signals due to the quadrupolar relaxation effects. The observation of very broad signals at *ca*. 0 ppm in ^{11}B NMR spectra owes to the use of borosilicate glass NMR tubes and boron-containing parts in the NMR cavity. Unless otherwise stated, all NMR spectra were recorded at 20 °C.

Mass spectrometry was performed by the *Scottish Instrumentation and Resource Centre for Advanced Mass Spectrometry* (SIRCAMS) at the University of Edinburgh using electron impact (EI) or electrospray ionisation (ESI) techniques. Mass spectrometry on the highly moisture sensitive sp^{3}C-aluminated products was attempted multiple times, however the molecular ion (or fragmentation product) was not observed, presumably due to the high sensitivity of these compounds to ambient atmosphere.

All crystal structures have been measured on a Rigaku Oxford Diffraction Synergy-S diffractometer using a Mo microfocus source and a Hypix Arc-100 detector. Crystallography

data have been deposited with the Cambridge crystallographic data centre as supplementary publication numbers 2455501. Copies of the data can be obtained free of charge on application to CCDC, 12 Union Road, Cambridge CB21EZ, UK (fax: (+44)1223-336-033; E-mail: deposit@ccdc.cam.ac.uk).

2. C(sp^3)−H metalation

Note, all C(sp^3)-H metalations were initially performed in J. Young's Tap NMR tubes using varying ratios of base : Lewis acid (including stoichiometric e.g., 1 base : 2 MX_3). These reactions are then monitored in-situ by NMR spectroscopy to allow for reaction conditions (e.g., temperature/time/ratios) to be optimised.

2.1 General Procedure 1

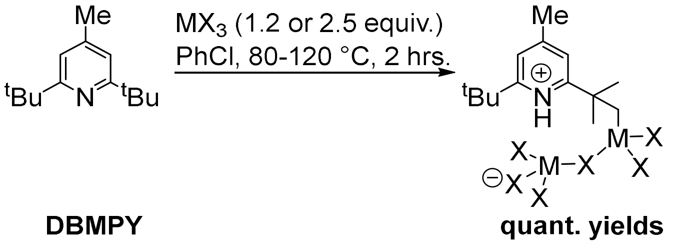

DBMPy (0.021 g, 0.10 mmol, 1 equiv.) and MX_3 (0.25/0.12 mmol, 2.5/1.2 equiv.) were suspended in chlorobenzene (1 mL) and heated to 80 °C or 100 °C in a sealed J. Young's Tap NMR tube for 2-18 hrs. Upon cooling, conversion was checked via NMR spectroscopy. An internal standard was added to the reaction mixture and the compound characterised through 1- and 2D-NMR spectroscopy in chlorobenzene and then in $CDCl_3$, respectively. Any residual solvent, by-products and impurities are noted as such underneath the spectra.

2.2 Lewis Acid Scope and Characterisation of Metallated DBMPy products from using MX_3 (Compounds 1-8)

Assignment of Aryl−H peaks in the reaction solvent, protio chlorobenzene, was verified through ^{1}H-^{13}C{^{1}H} HSQC NMR and where possible ^{1}H-^{1}H COSY NMR. However, multiplicities, integrals and sometimes the product Aryl−H peaks could not be determined in chlorobenzene due to coincidence with the solvent resonance. The diagnostic CH_2 (Al−CH_2) resonance was verified through ^{1}H-^{13}C{^{1}H} HSQC NMR and the characteristic cross peak of Al−CH_2 with the broad ^{13}C resonance of C-Al (br s). Cyclohexane (10 µL, 0.093 mmol) was used as internal standard to quantify the yield. In case of overlap of signals of the product/by-product with the internal standard in the ^{1}H NMR spectra, 1,3,5-trimethyl- or 1,3,5-trimethoxy-benzene were used instead.

2.3. DBMPy functionalisation with $AlCl_3$ (1)

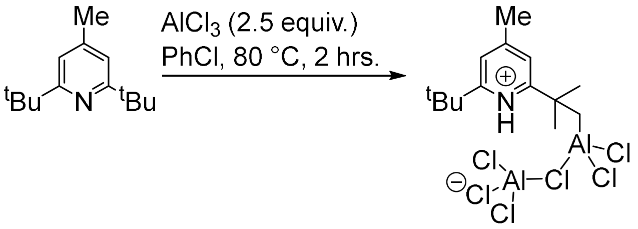

1

Following **General Procedure 1,** compound **1** was prepared using $AlCl_3$ (0.033 g, 0.25 mmol, 2.5 equiv.) at 80 °C for 2 hrs. The product was obtained in essentially quantitative yield (>95% yield vs internal standard).

^{1}H NMR (500 MHz, PhCl) δ 10.54 (br s, 1H), 7.28, 7.10, 2.22 (s, 3H), 1.45 (s, 6H), 1.19 (s, 9H), 1.17 (s, 2H). **^{13}C{^{1}H} NMR (126 MHz, PhCl)** δ 162.99, 162.18, 161.20, 122.92, 122.47, 38.82, 36.06, 30.14, 29.26 (br s), 28.37, 22.55. **^{27}Al NMR** (130 MHz, PhCl) δ not observed.

^{1}H NMR (500 MHz, $CDCl_3$) 10.81 (br s, 1H), 7.66 (m, 1H), 7.57 (m, 1H), 2.72 (s, 3H), 1.69 (s, 6H), 1.58 (s, 9H), 1.19 (s, 2H). **^{13}C{H} NMR (126 MHz, $CDCl_3$)** δ 163.63, 162.35, 161.56, 123.55, 123.03, 39.29, 36.75, 30.86, 29.47 (br s), 29.23, 23.41. **^{27}Al NMR (130 MHz, $CDCl_3$)** δ not observed.

2.4. DBMPy functionalisation with $AlBr_3$ (6)

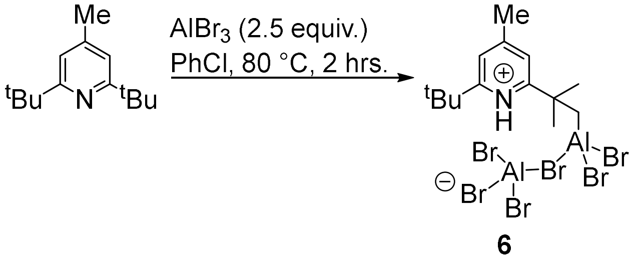

6

Compound **6** was prepared following **General Procedure 1** with $AlBr_3$ (0.067 g, 0.25 mmol, 2.5 equiv.) at 80 °C for 2 hrs. The product was obtained in essentially quantitative yield (98% yield vs internal standard).

^{1}H NMR (500 MHz, PhCl) δ 10.53 (br s, 1H), 7.32, 7.11, 2.22 (s, 3H), 1.50 (s, 6H), 1.41 (s, 2H),1.24 (s, 9H). **^{13}C{^{1}H} NMR (126 MHz, PhCl, 300 K)** δ 162.98, 162.09, 161.16, 123.21, 122.56, 39.36, 36.12, 31.89 (br s), 30.30, 28.58, 22.71.

¹H NMR (500 MHz, CDCl₃) δ 10.83 (br s, 1H), 7.64 (s, 1H), 7.53 (s, 1H), 2.70 (s, 3H), 1.66 (s, 6H), 1.56 (s, 9H), 1.19 (s, 2H). **^{13}C{^{1}H} NMR (126 MHz, $CDCl_3$, 300 K)** δ 164.02, 162.12, 161.53, 123.57, 122.84, 39.40, 36.75, 30.99, 29.25, 29.22, 23.39. **^{27}Al NMR (130 MHz, $CDCl_3$)** δ 101.82, 93.39.

2.5. DBMPy functionalisation with AlI_3 (7)

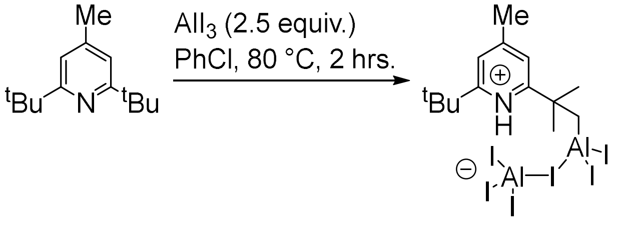

Compound **7** was prepared following **General Procedure 1** with AlI_3 (0.102 g, 0.25 mmol, 2.5 equiv.) at 80 °C for 2 hrs. The product was obtained in essentially quantitative conversion. The internal standard cyclohexane overlapped with the title compound resonances and other internal standards explored (e.g., nitromethane, dichloroethane) reacted with **7**. The yield (99% vs internal standard) could only be determined using nitromethane with spectra recorded within 10 minutes of addition of nitromethane. The resonances corresponding to C-aluminated product shifted significantly (possibly indicating $MeNO_2$-AlI_3 adduct formation) but integrated accurately.

^{1}H NMR (500 MHz, PhCl) δ 10.49 (br s, 1H), 7.42 (m, 1H), 2.24 (s, 3H), 1.82 (s, 2H), 1.55 (s, 6H), 1.23 (s, 9H). *Note: Not all Aryl-H resonances could be observed because of overlap with PhCl.* **^{13}C{^{1}H} NMR (126 MHz, PhCl, 300 K)** δ 162.44, 161.78, 160.90, 123.40, 122.49, 40.08, 35.97, 33.12 (br s), 30.14, 28.63, 22.66. **^{27}Al NMR (130 MHz, PhCl)** δ not observed.

^{1}H NMR (500 MHz, $CDCl_3$) δ 10.80 (br s, 1H), 7.62 (m, 1H), 7.53 (m, 1H), 2.69 (s, 3H), 1.64 (s, 6H), 1.55 (s, 9H), 1.07 (s, 2H). **^{13}C{^{1}H} NMR (126 MHz, $CDCl_3$, 300 K)** δ 163.87, 162.03, 161.33, 123.54, 122.89, 39.35, 36.69, 31.01, 29.56, 29.23, 23.38. **^{27}Al NMR (130 MHz, $CDCl_3$)** δ not observed.

2.6. DBMPy functionalisation with $GaCl_3$ (8)

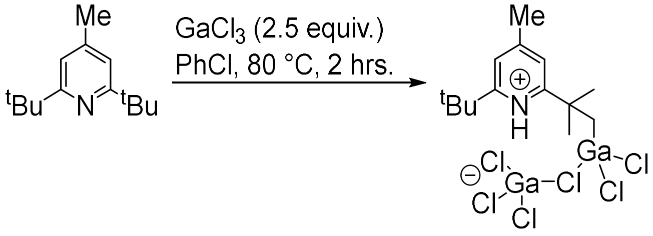

8

Compound **7** was prepared following **General Procedure 1** with $GaCl_3$ (0.044 g, 0.25 mmol, 2.5 equiv.) at 80 °C for 2 hrs. The product was obtained in essentially quantitative yield (96% yield vs internal standard).

After the title compound precipitated as an oil in chlorobenzene, crystals suitable for X-Ray diffraction were obtained on standing after 2 months from the oil (see section 14.1. Crystal structure of compound **8-GaCl3**).

^{1}H NMR (500 MHz, PhCl) δ 10.37 (br s, 1H), 7.35 (m, 1H), 7.25 (m, 1H), 2.32 (s, 3H), 1.90 (s, 2H), 1.47 (s, 6H), 1.21 (s, 9H). **^{13}C{^{1}H} NMR** (126 MHz, PhCl, 300 K) δ 163.42, 162.32, 159.59, 123.79, 123.47, 38.92, 38.22 (br s), 36.36, 29.09, 28.42, 22.87.

^{1}H NMR (500 MHz, $CDCl_3$) δ 10.74 (br s, 1H), 7.74 (s, 1H), 7.61 (s, 1H), 2.75 (s, 3H), 2.08 (s, 2H), 1.76 (s, 6H), 1.58 (s, 9H). **^{13}C{^{1}H} NMR** (126 MHz, $CDCl_3$, 300 K) δ 163.12, 162.27, 161.09, 124.28, 123.84, 39.61, 36.98, 29.95, 29.32, 29.28, 23.58.

3. NMR Spectra of Metalated DBMPy

NMR Spectra of (**1**):

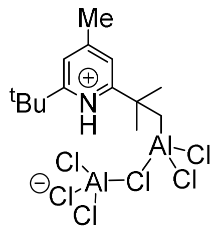

1

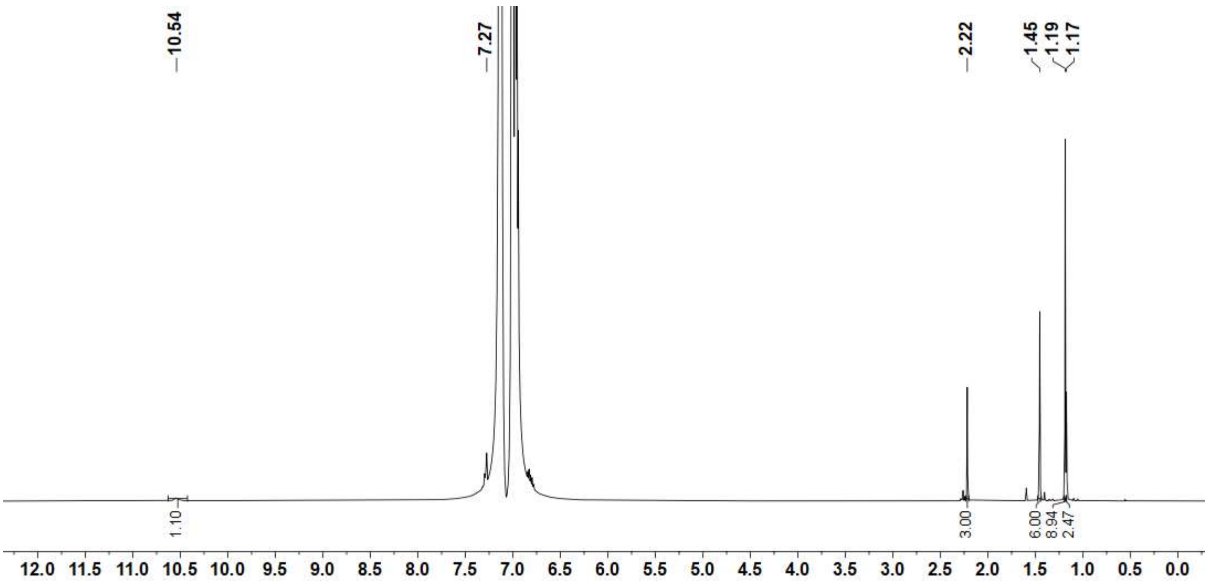

Figure S1: ^{1}H NMR spectrum of (**1**) in PhCl. *Note: The small impurity resonances are due to protonated 2,6-^{t}Bu-4-methyl-pyridine.*

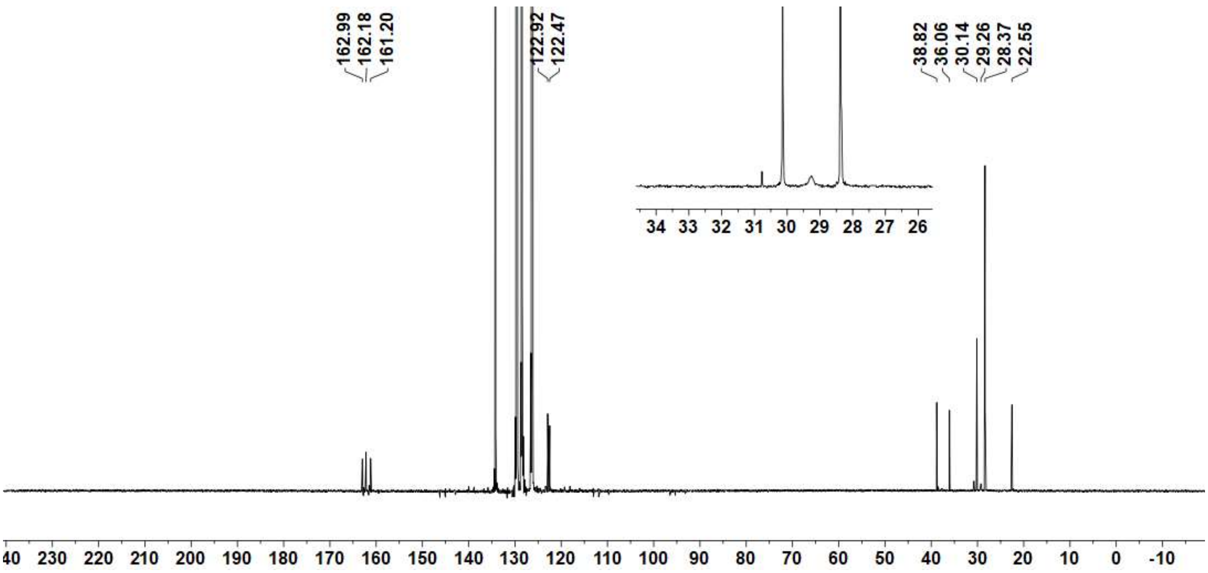

Figure S2: $^{13}C\{^{1}H\}$ NMR spectrum of (**1**) in PhCl.

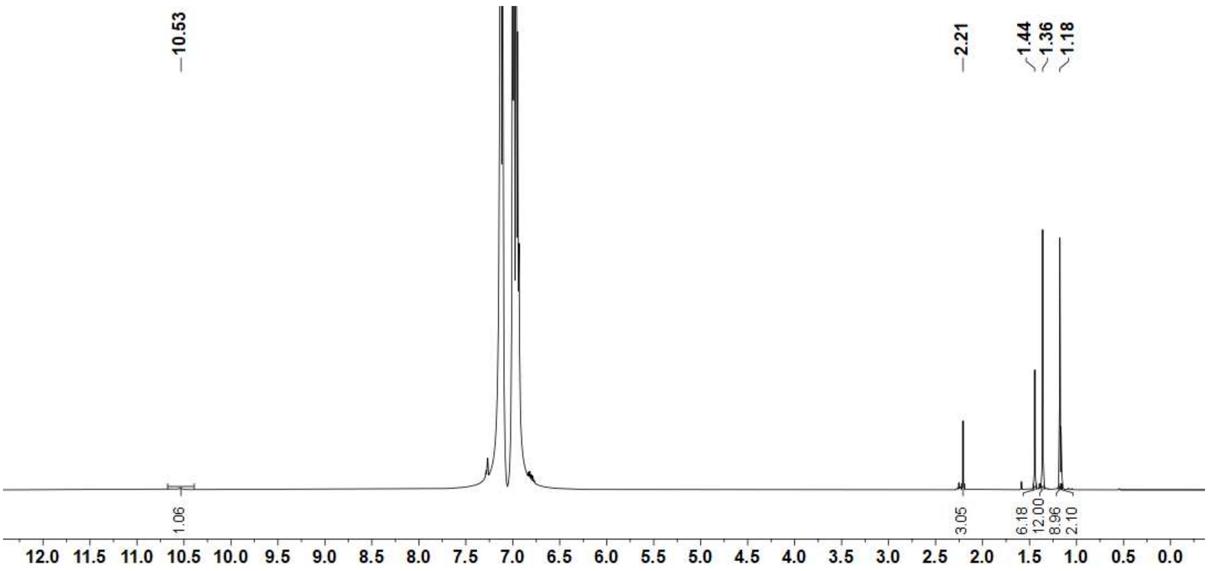

Figure S3: ^{1}H NMR spectrum of (**1**) in PhCl with internal standard (cyclohexane). *Note: small impurity resonances at 2.30 ppm and 1.68 ppm correspond to protonated 2,6-^{t}Bu-4-methyl-pyridine.*

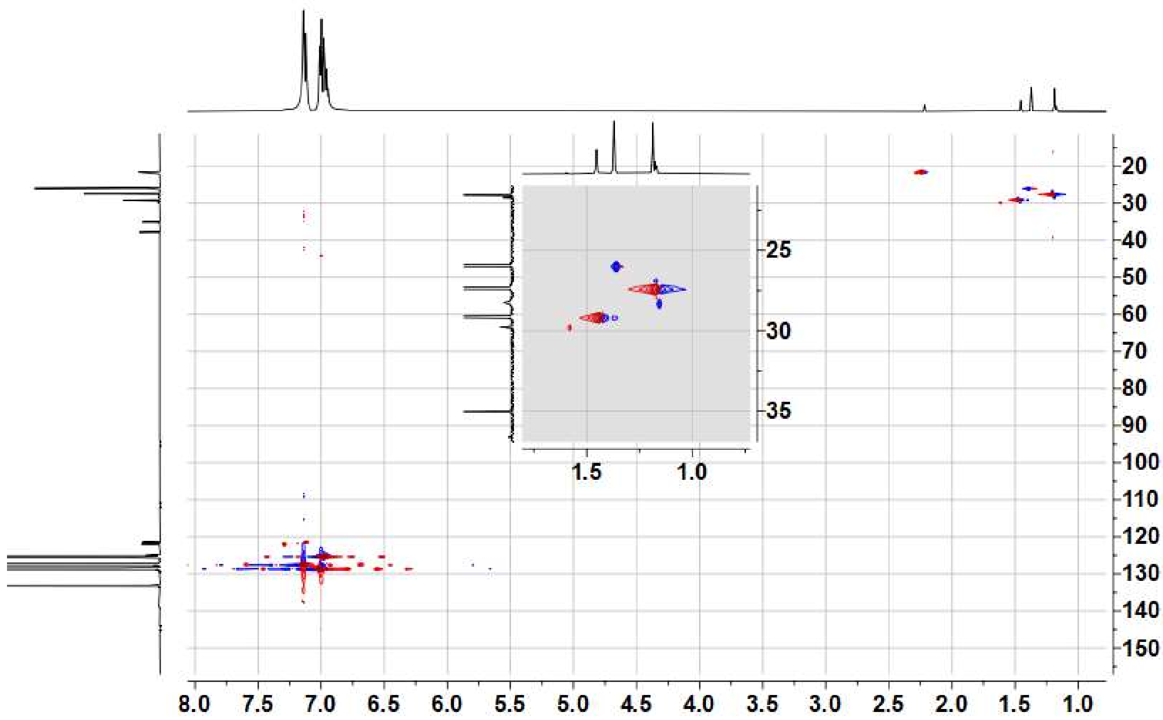

Figure S4: $^{1}H-^{13}C\{^{1}H\}$ HSQC NMR spectrum of (**1**) in PhCl with internal standard (cyclohexane).

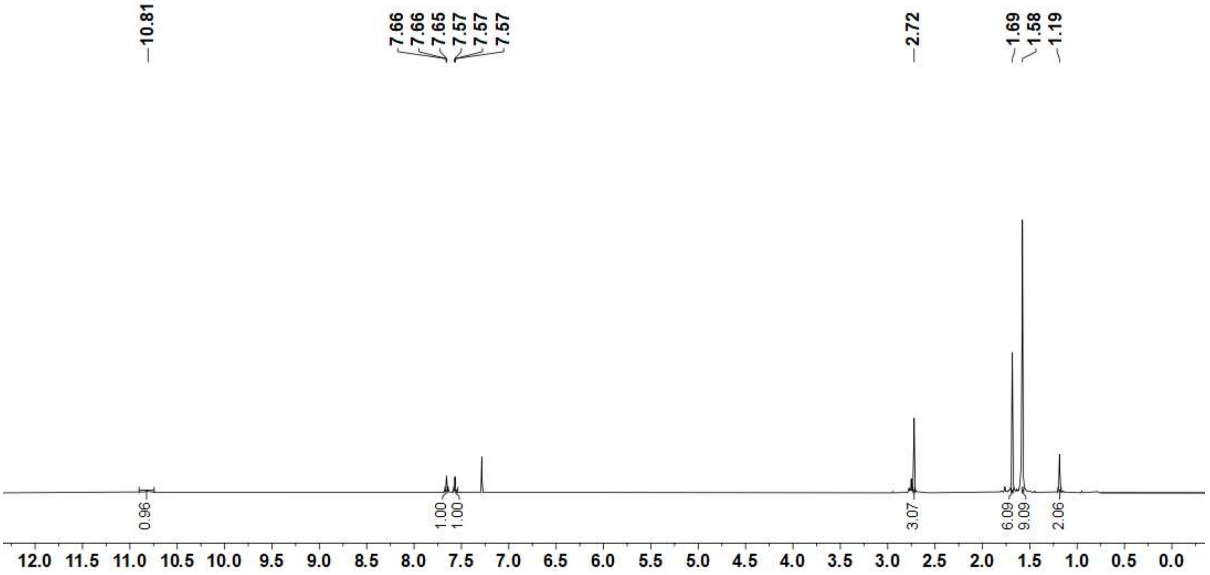

Figure S5: ^{1}H NMR spectrum of (**1**) in $CDCl_3$.

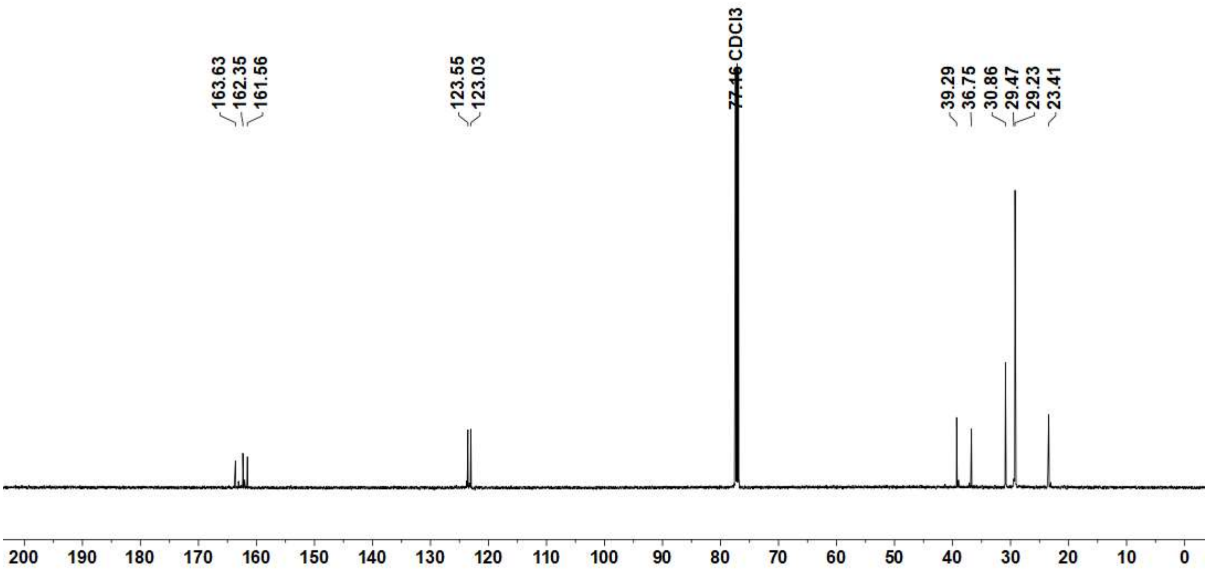

Figure S6: $^{13}C\{^{1}H\}$ NMR spectrum of (**1**) in $CDCl_3$.

NMR Spectra of (**6**):

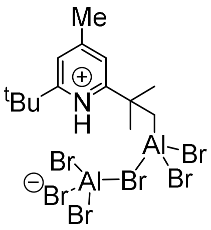

6

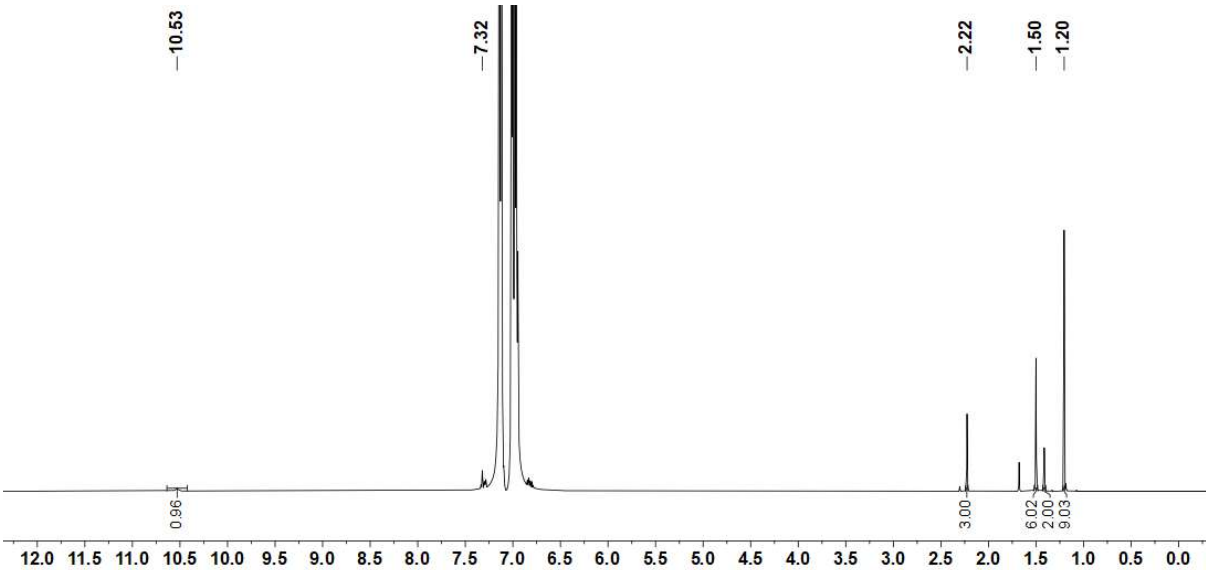

Figure S7: ^{1}H NMR spectrum of (**6**) in PhCl. *Note: small impurity resonances at 2.30 ppm and 1.68 ppm correspond to protonated 2,6-^{t}Bu-4-methyl-pyridine.*

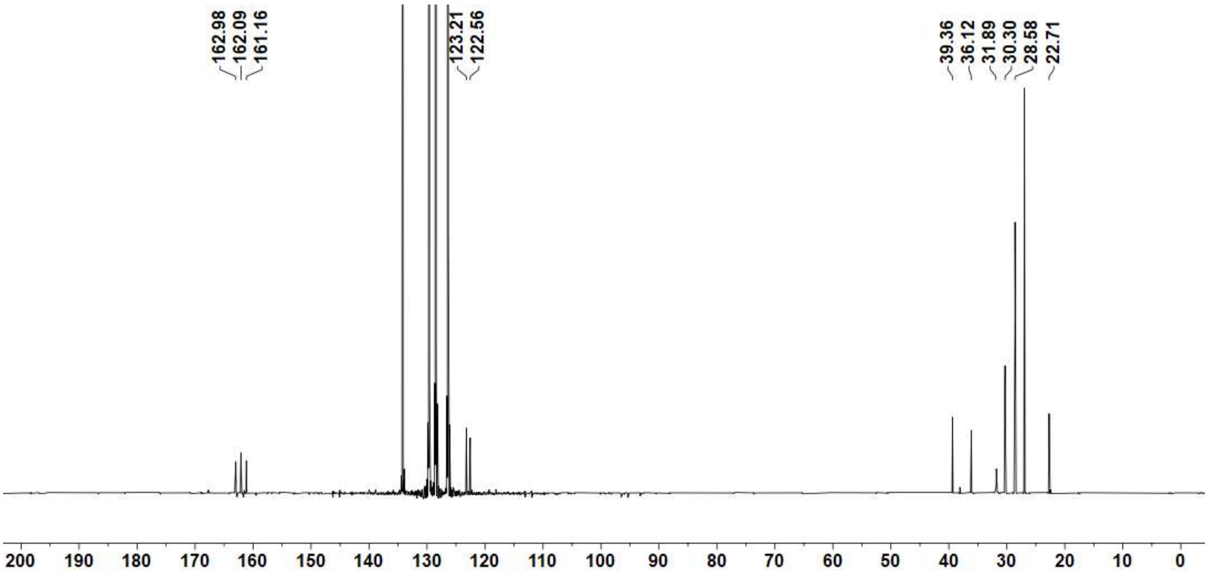

Figure S8: $^{13}C\{^{1}H\}$ NMR spectrum of (**6**) in PhCl with internal standard (cyclohexane).

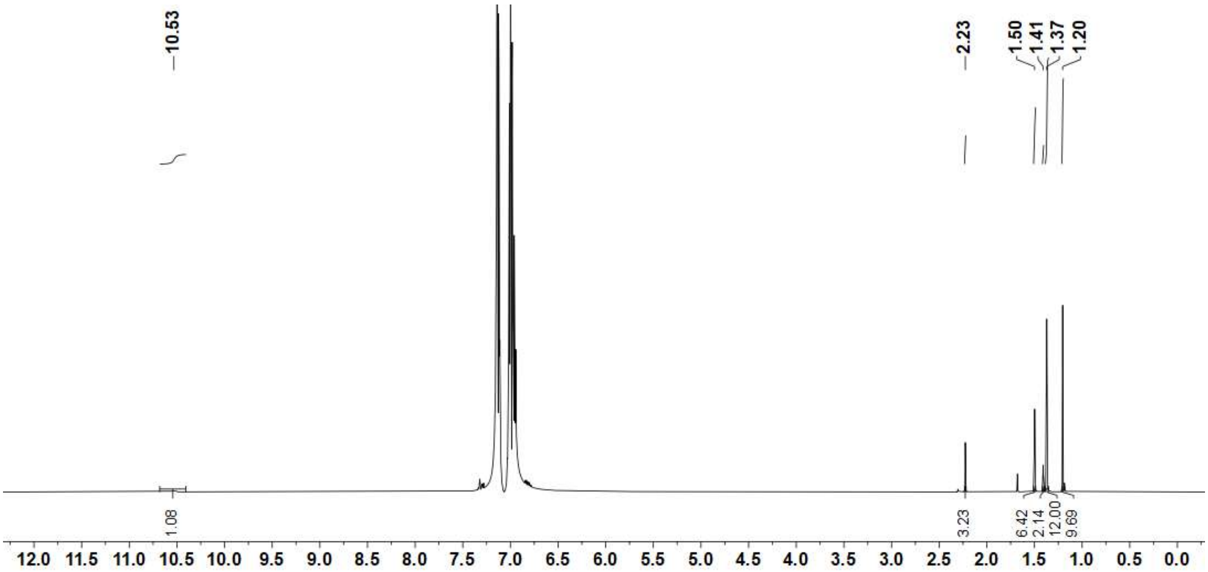

Figure S9: ^{1}H NMR spectrum of (**6**) in PhCl with internal standard (cyclohexane). *Note: small impurity resonances at 2.30 ppm and 1.68 ppm correspond to protonated 2,6-^{t}Bu-4-methyl-pyridine.*

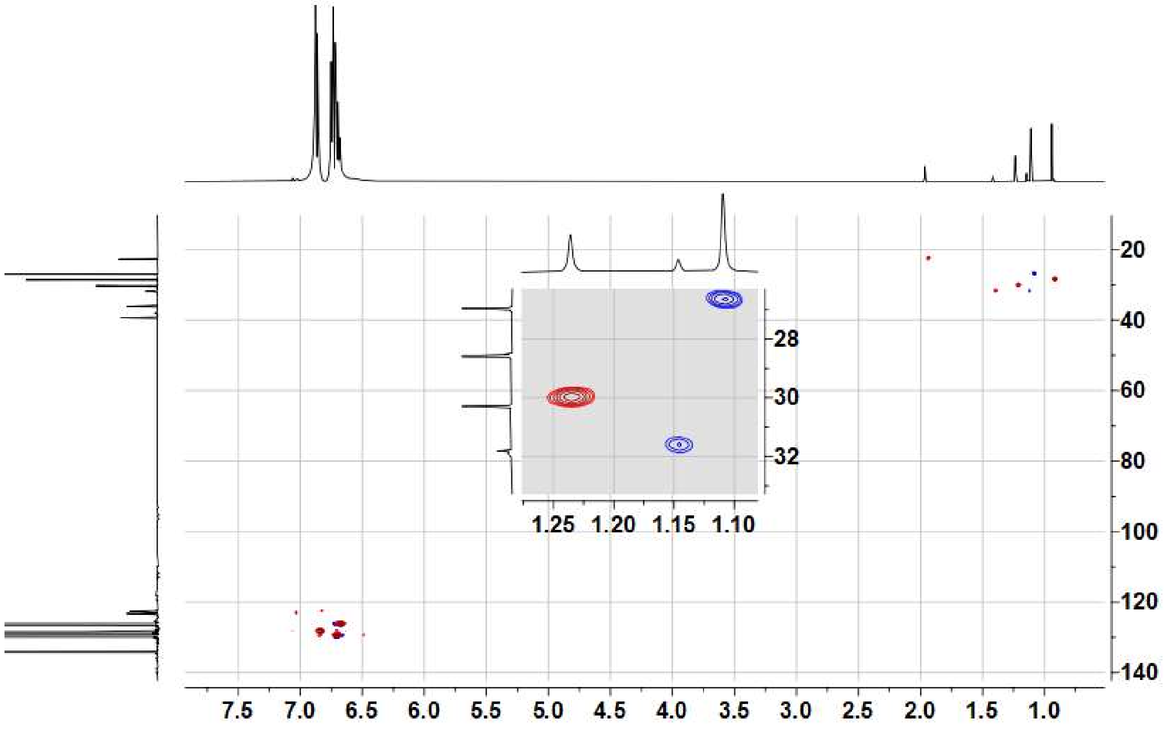

Figure S10: $^{1}H-^{13}C\{^{1}H\}$ HSQC NMR spectrum of (**6**) in PhCl with internal standard (cyclohexane).

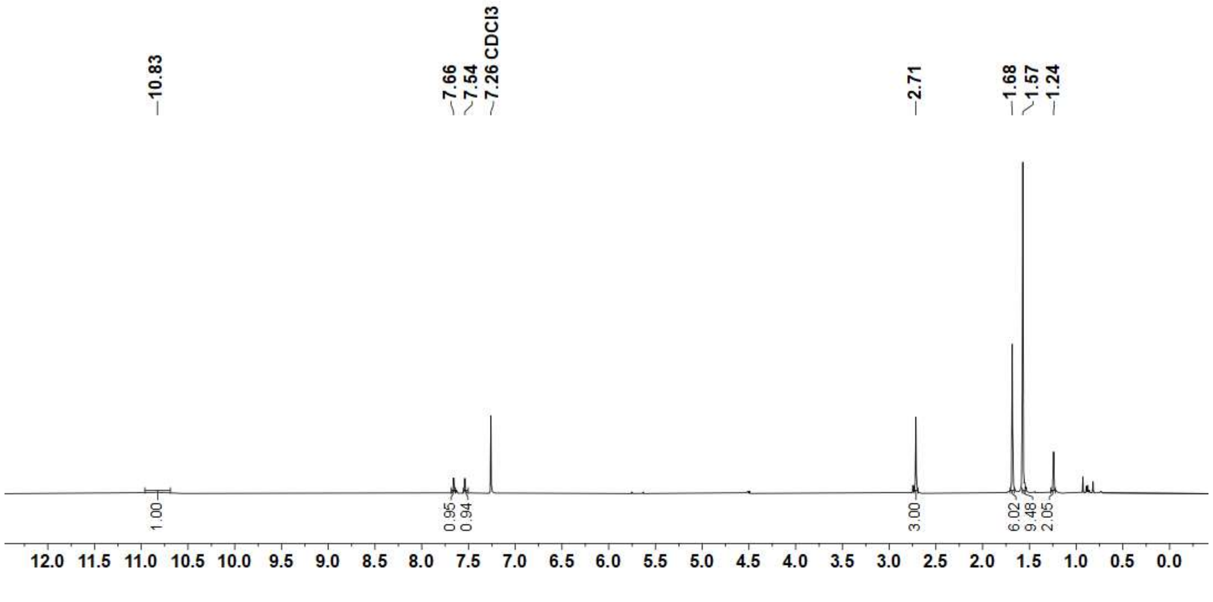

Figure S11: ^{1}H NMR spectrum of (**6**) in $CDCl_3$.

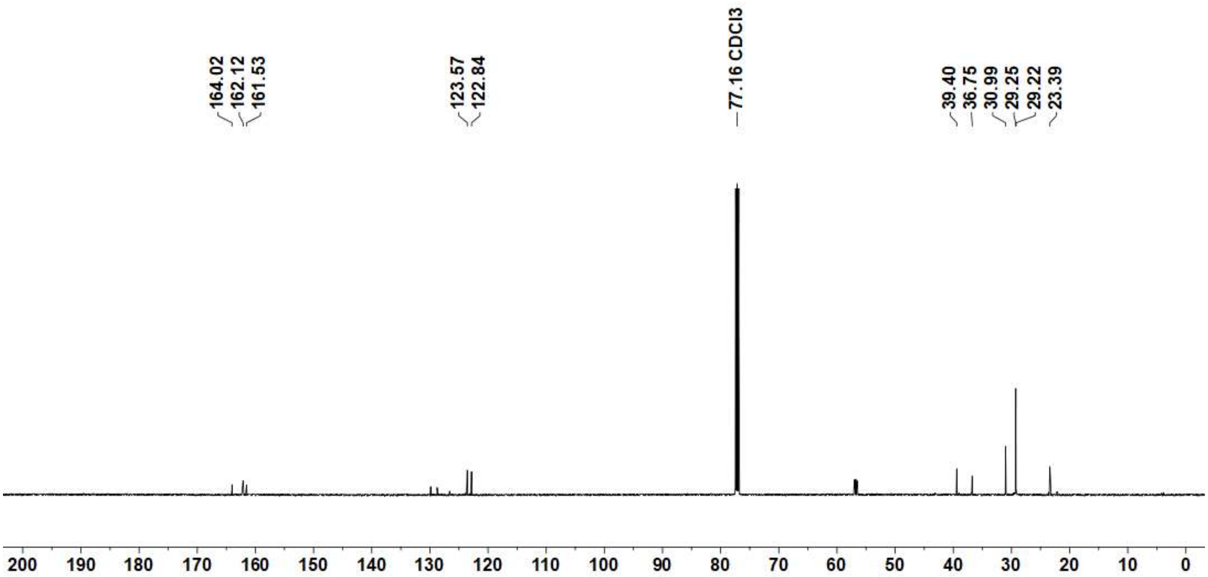

Figure S12: $^{13}C\{^{1}H\}$ NMR spectrum of (**6**) in $CDCl_3$. *Note: small impurity resonances at 134.35, 129.86, 128.73 and 126.59 ppm correspond to chlorobenzene. Resonance at 56 ppm corresponds to DCM.*

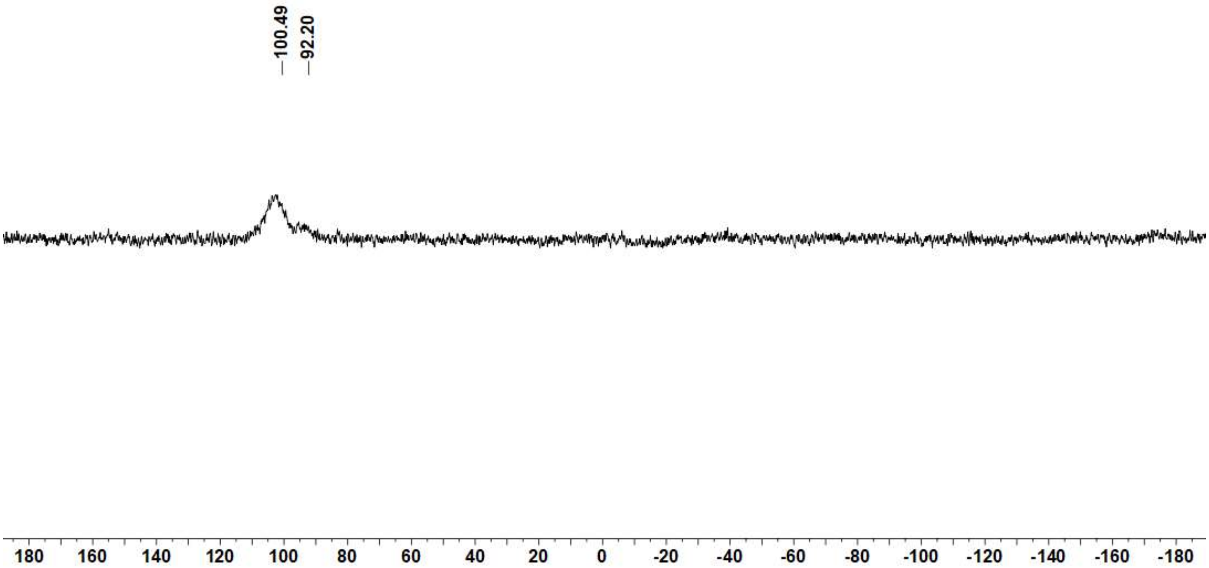

Figure S13: ^{27}Al NMR spectrum of (**6**) in $CDCl_3$.

NMR Spectra of (**7**):

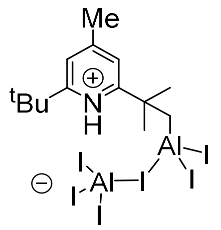

7

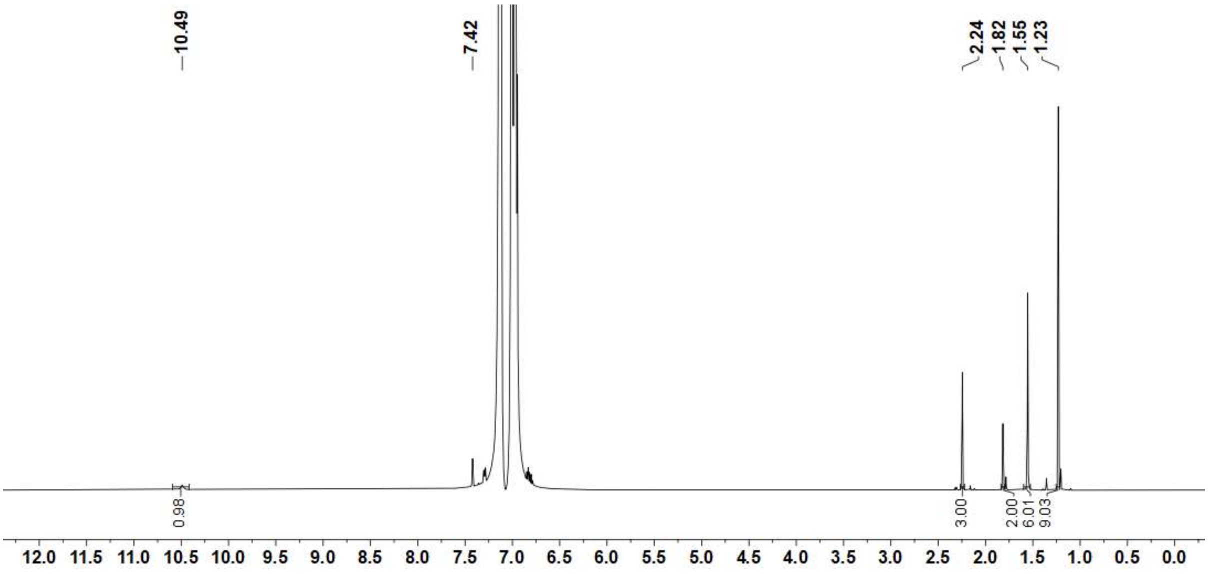

Figure S14: ^{1}H NMR spectrum of (**7**) in PhCl.

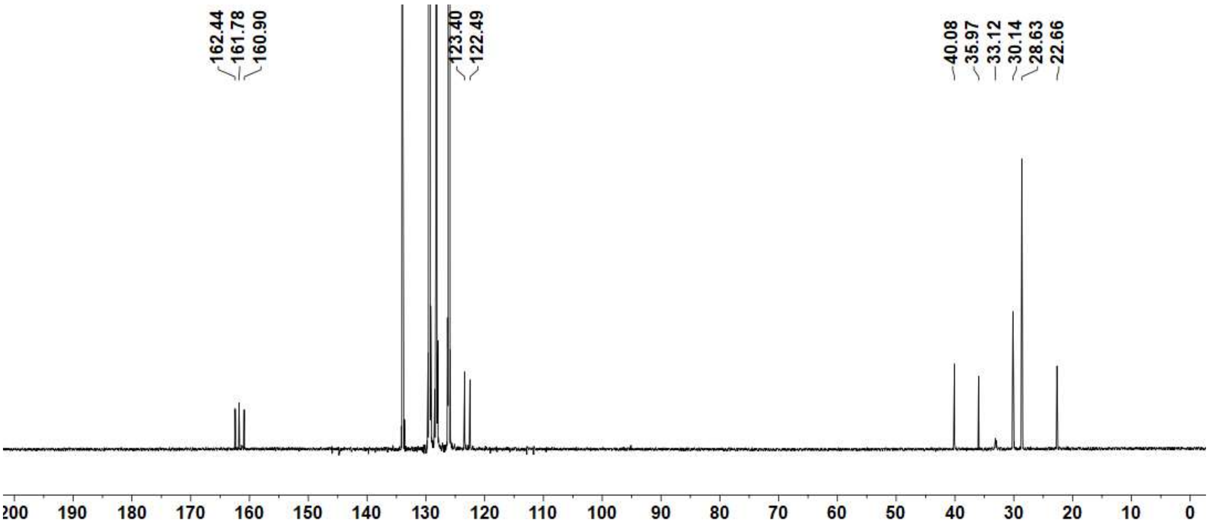

Figure S15: $^{13}C\{^{1}H\}$ NMR spectrum of (**7**) in PhCl.

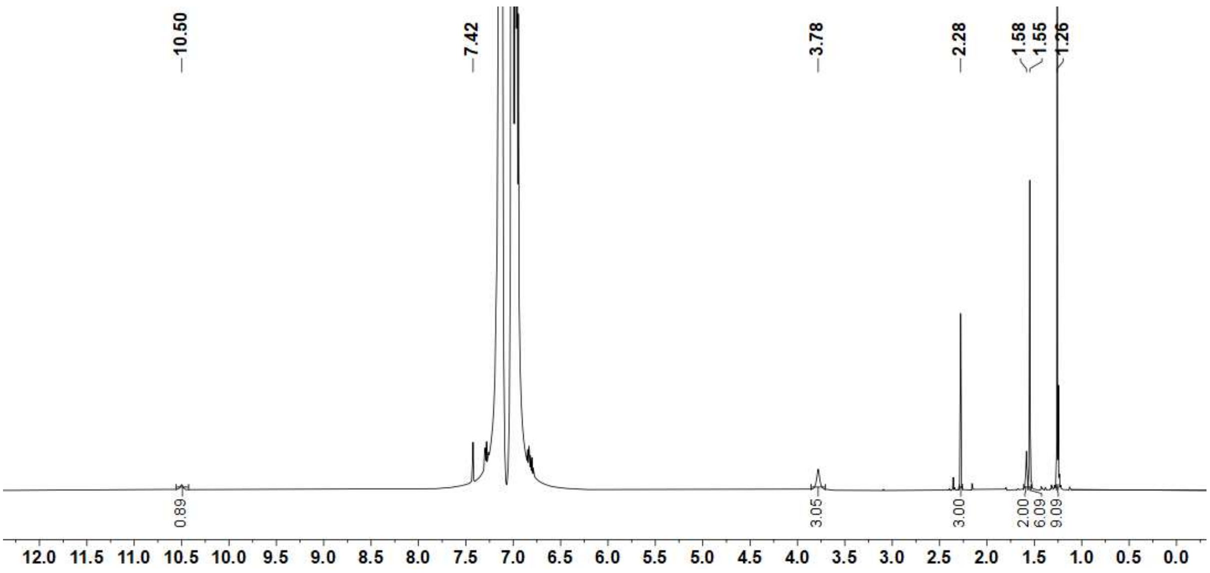

Figure S16: ^{1}H NMR spectrum of (**7**) in PhCl with internal standard (nitromethane). *Note: addition of nitromethane resulted in a shift of the Al−CH_2 resonance (to 1.58 ppm).*

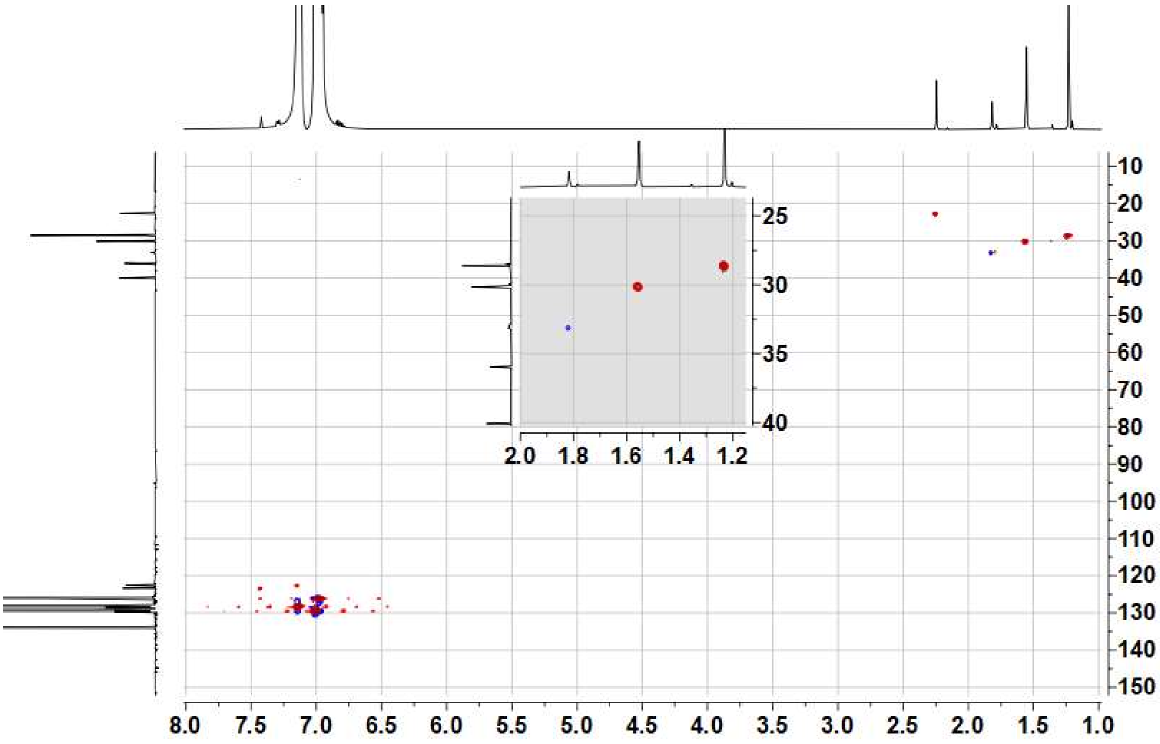

Figure S17: ^{1}H−^{13}C{^{1}H} HSQC NMR spectrum of (**7**) in PhCl.

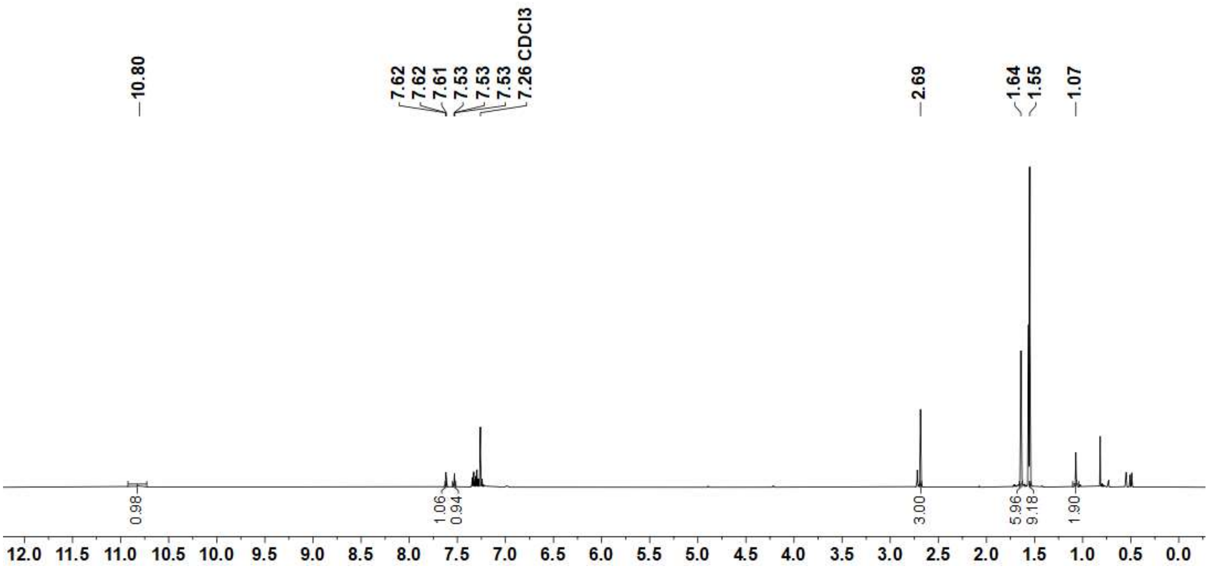

Figure S18: ^{1}H NMR spectrum of (**7**) in $CDCl_3$. *Note: impurity resonances at 7.30 ppm correspond to chlorobenzene and at 10.59 (br s), 2.72, 1.56 ppm correspond to protonated 2,6-^{t}Bu-4-methyl-pyridine.*

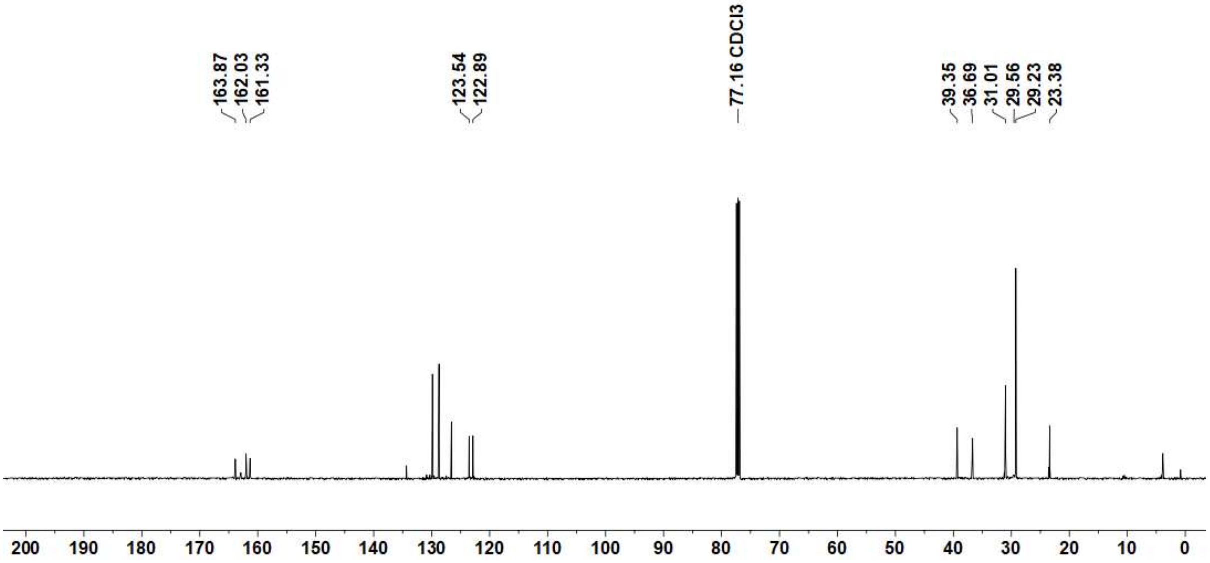

Figure S19: $^{13}C\{^{1}H\}$ NMR spectrum of (**7**) in $CDCl_3$. *Note: impurity resonances at 134.35, 129.86, 128.73 and 126.59 ppm correspond to chlorobenzene.*

NMR Spectra of (**8**):

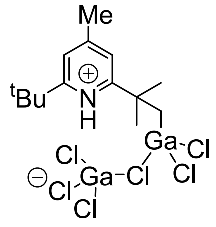

8

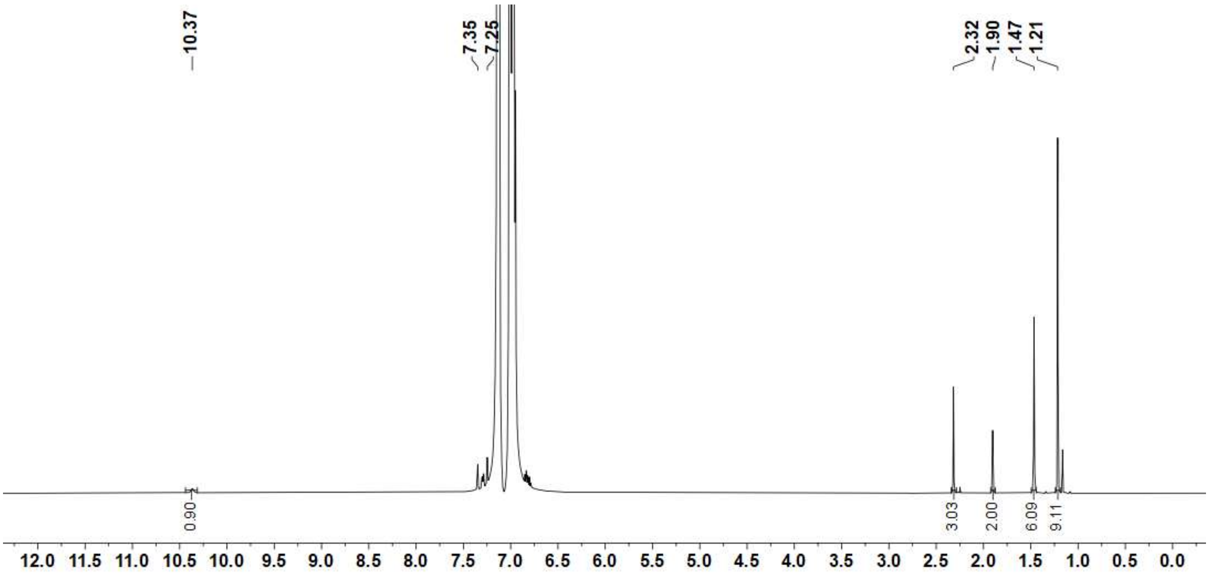

Figure S20: ^{1}H NMR spectrum of (**8**) in PhCl. *Note: resonances at 2.25 ppm and 1.16 ppm correspond to unreacted starting material (2,6-^{t}Bu-4-methyl-pyridine).*

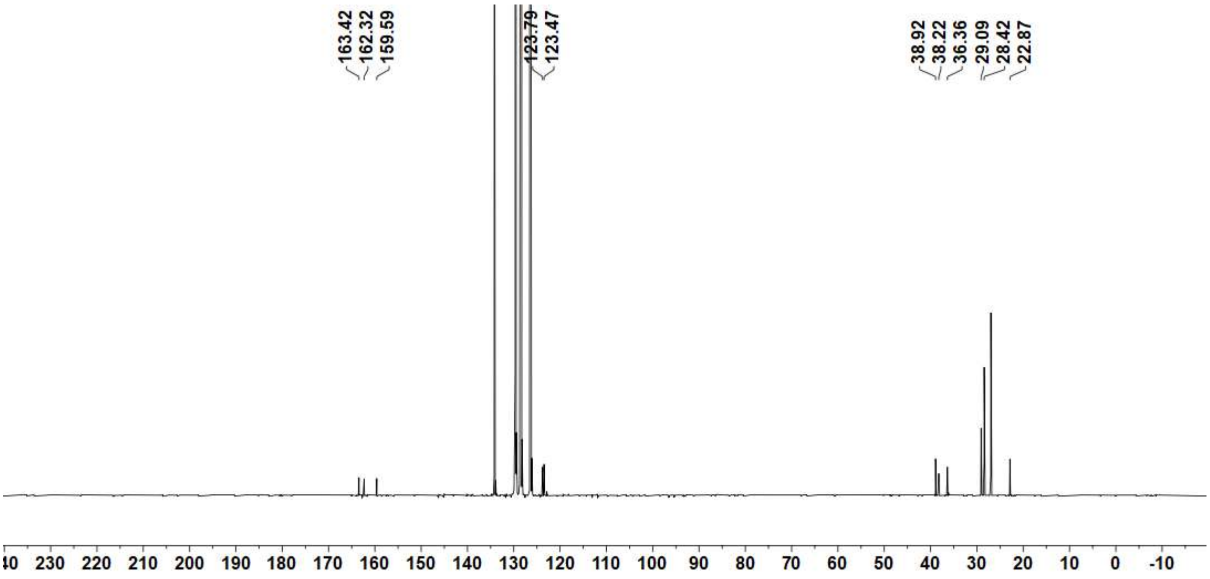

Figure S21: $^{13}C\{^{1}H\}$ NMR spectrum of (**8**) in PhCl with internal standard (cyclohexane).

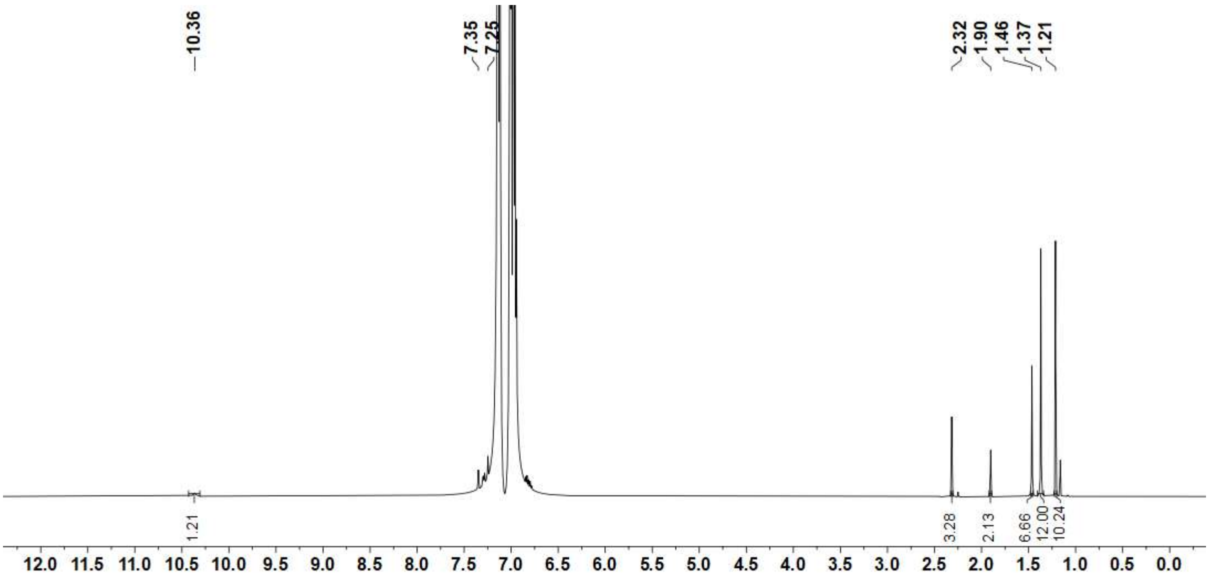

Figure S22: ^{1}H NMR spectrum of (**8**) in PhCl with internal standard (cyclohexane). *Note: resonance at 2.25 ppm and 1.16 ppm correspond to unreacted starting material (2,6-^{t}Bu-4-methyl-pyridine).*

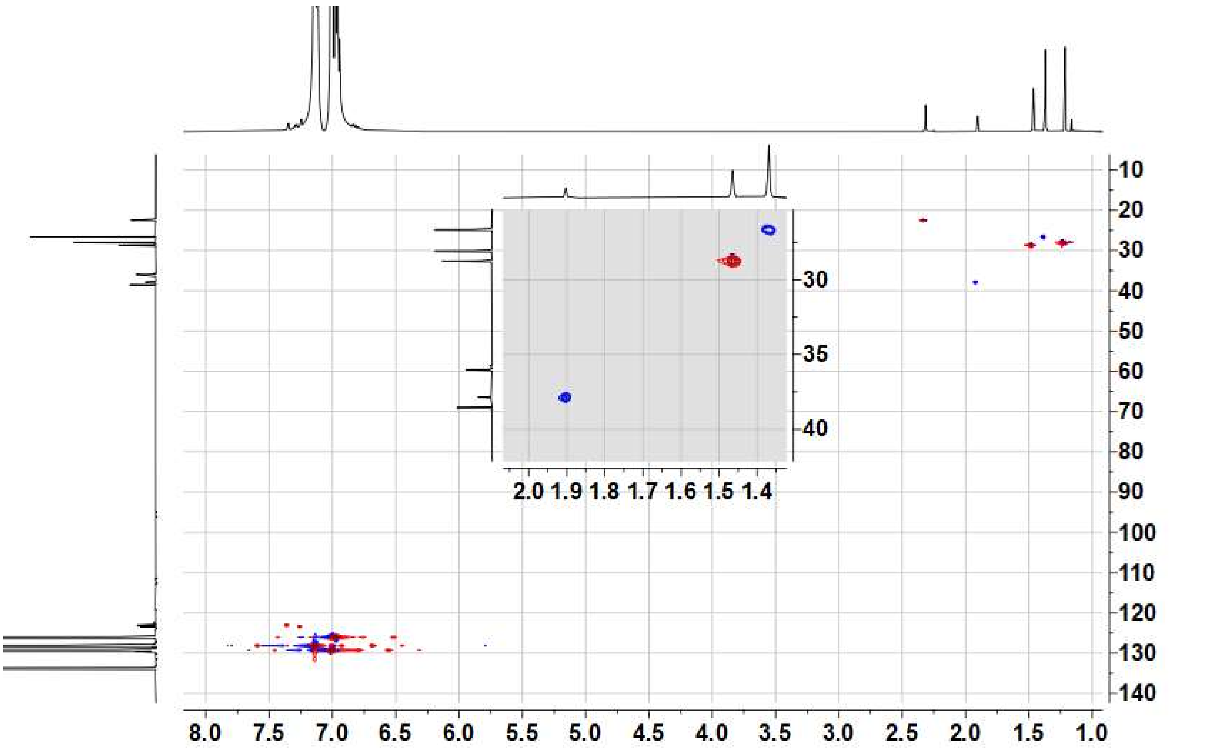

Figure S23: $^{1}H-^{13}C\{^{1}H\}$ HSQC NMR spectrum of (**8**) in PhCl with internal standard (cyclohexane).

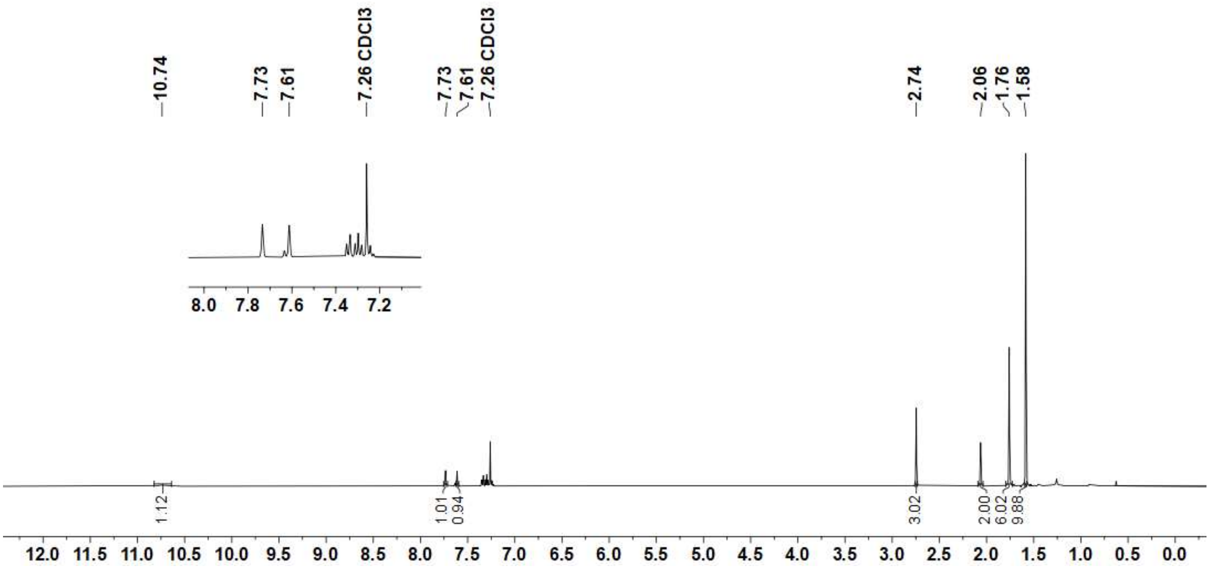

Figure S24: ^{1}H NMR spectrum of (**8**) in $CDCl_3$. *Note: impurity resonances at ca. 7.30 ppm correspond to chlorobenzene.*

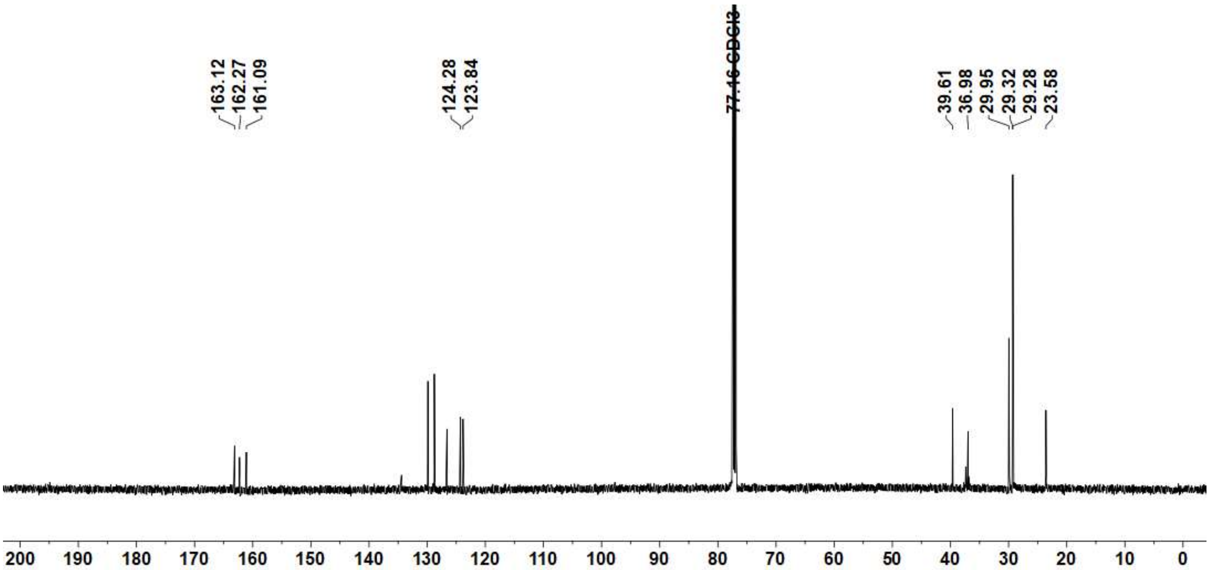

Figure S25: $^{13}C\{^1H\}$ NMR spectrum of (**8**) in $CDCl_3$. *Note: impurity resonances at 134.35, 129.86, 128.73 and 126.59 ppm correspond to chlorobenzene.*

4. Synthesis and Activation of Pyrazaboles

For further functionalisation of DBMPy pyrazabole and its derivatives were synthesised.

4.1. Synthesis of pyrazabole

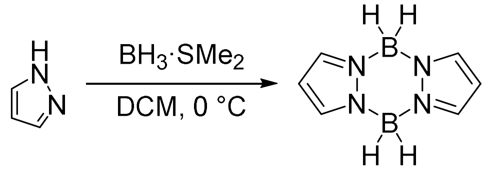

Neat $BH_3{\cdot}SMe_2$ (2.0 ml, 21.1 mmol, 1 equiv.) was slowly added to a solution of pyrazole (1.435 g, 21.1 mmol, 1 equiv.) in DCM (10 ml) at 0 °C. After the initial gas evolution stopped the reaction was heated for 18 hrs at 35 °C in an open system under inert atmosphere. All volatiles were removed *in vacuo* and the remaining white solid was sublimed (120 °C, $4x10^{-2}$ mbar) to yield the product as a white powder in 64% yield (1.08 g, 10.45 mmol). Analytical data are in accordance with literature values.[1]

^{1}H NMR (400 MHz, $CDCl_3$) δ 7.62 (d, *J* = 2.3 Hz, 2H), 6.31 (t, *J* = 2.4 Hz, 1H), 3.77 (s, 1H), 3.46 (s, 1H), 3.22 (s, 0H); **^{11}B NMR (128 MHz, $CDCl_3$)** δ − 8.43 (t, *J* = 111.2 Hz); **^{11}B{^{1}H} NMR (128 MHz, $CDCl_3$)** δ − 8.45 (s)

4.2 Synthesis of dichloropyrazabole

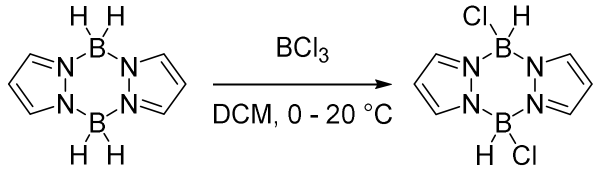

Pyrazabole (1 g, 6.24 mmol, 1.5 equiv.) was suspended in DCM (10 mL). The flask was attached to a bubbler filled with methanol (*Note: this is to quench produced diborane*). The reaction flask was cooled to 0 °C and BCl_3 (4.25 mL (1M in DCM), 4.25 mmol, 1 equiv.) added dropwise. The reaction was left to warm to room temperature overnight. Leftover solvent was filtered off and the remaining crude dried. No further purification was necessary and the title compound was recovered as a white powder in 64% yield (0.89 g, 3.99 mmol).

^{1}H NMR (400 MHz, $CDCl_3$) δ 7.62 (d, *J* = 2.3 Hz, 2H), 6.31 (t, *J* = 2.4 Hz, 1H), 3.77 (s, 1H), 3.46 (s, 1H), 3.22 (s, 0H); **^{11}B NMR (128 MHz, $CDCl_3$)** δ -8.43 (t, *J* = 111.2 Hz); **^{11}B{^{1}H} NMR (128 MHz, $CDCl_3$)** δ - 8.45; Analytical data are in accordance with literature values. [2]

5. NMR Spectra of Synthesised Pyrazabole species

NMR spectra of dichloropyrazabole:

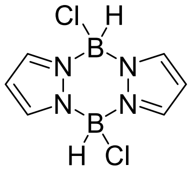

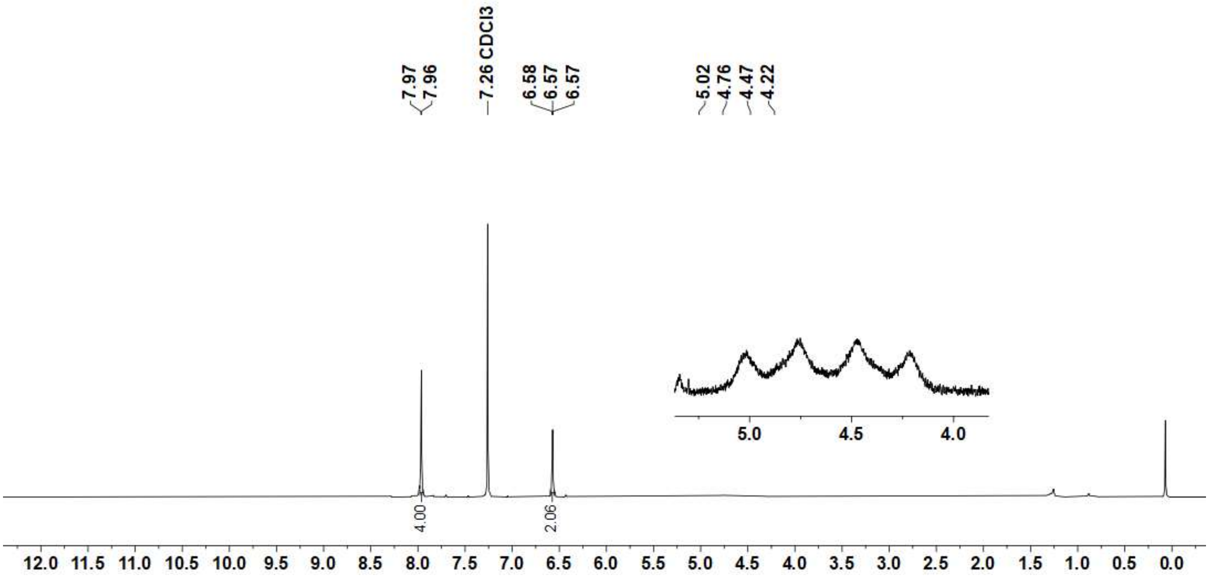

Figure S26: ^{1}H NMR spectrum of dichloropyrazabole in $CDCl_3$ with an included close up of the broad B−H signals.

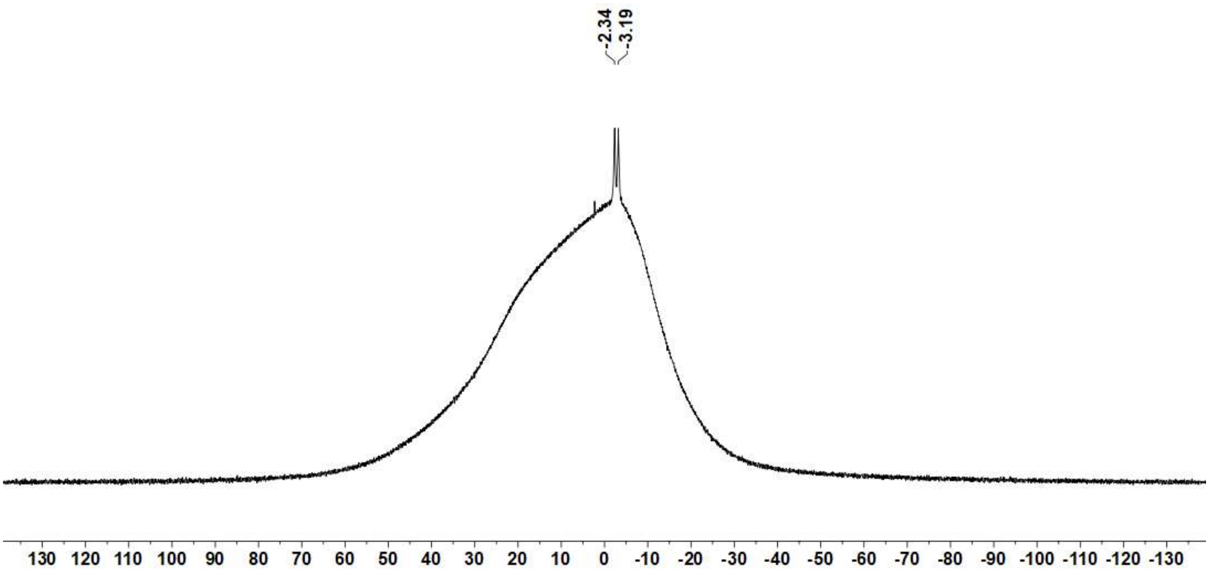

Figure S27: ^{11}B NMR spectrum of dichloropyrazabole in $CDCl_3$.

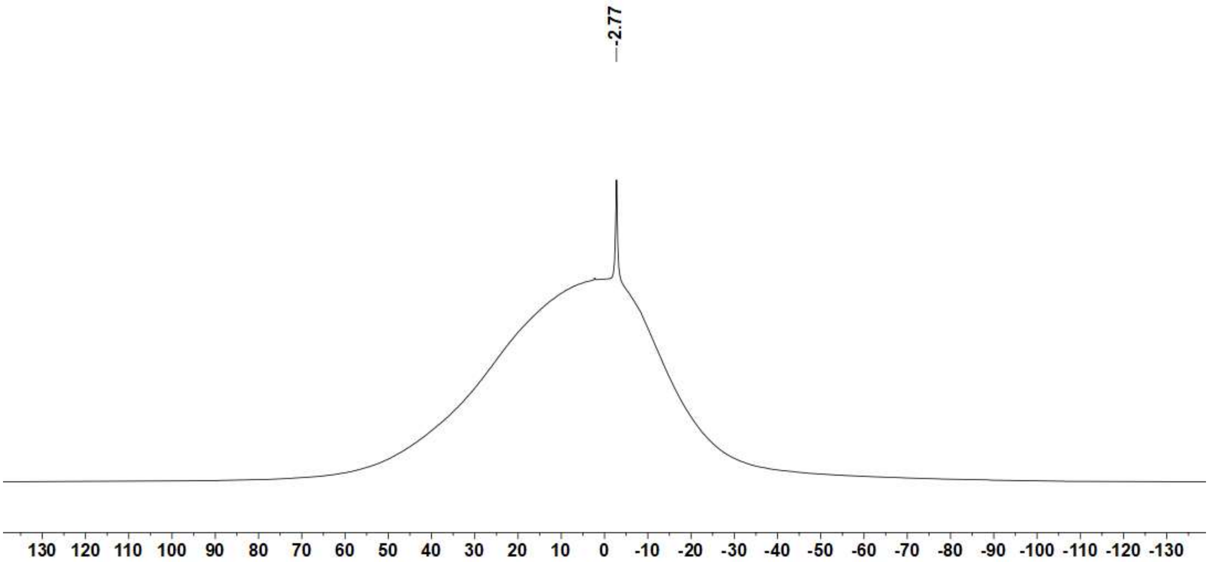

Figure S28: $^{11}B\{^1H\}$ NMR spectrum of dichloropyrazabole in $CDCl_3$.

6. Functionalisation of metalated DBMPy

6.1. General Procedure 2 – Transmetallation using a Pyrazabole Derivative

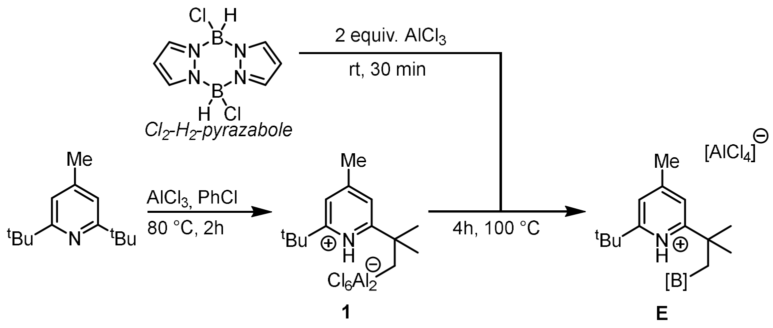

Dichloropyrazabole (0.068 g, 0.3 mmol, 1 equiv.) was added to $AlCl_3$ (0.078 g, 0.6 mmol, 2 equiv.) and stirred at room temperature for 30 minutes until everything dissolved in chlorobenzene (2 mL). DBMPy (0.063 g, 0.3 mmol, 1 equiv.) and $AlCl_3$ (0.099 g, 0.75 mmol, 0.25 equiv.) were suspended in chlorobenzene (3 mL) and heated to 80 °C in a sealed J. Young's Tap ampule tube for 2 hrs. The reaction mixture of $AlCl_3$ and dichloropyrazabole was transferred to the metalated species and the mixture heated for 4h at 100 °C.

6.2. Synthesis of the C(sp^3)−H borylated, pinacol protected DBMPy (2)

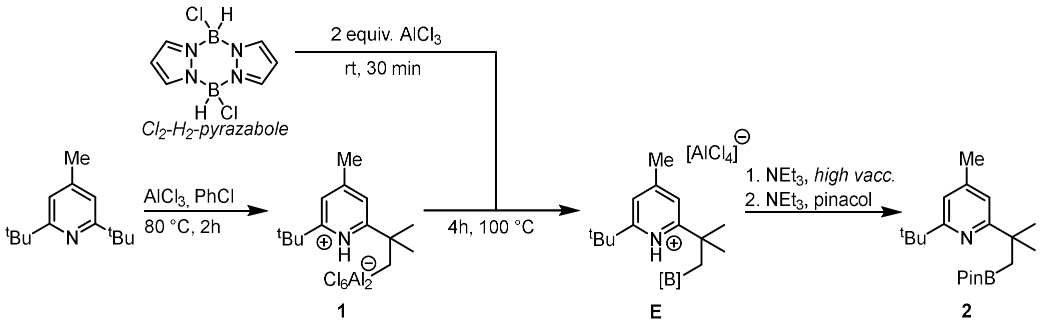

Compound **1** is prepared according to **General Procedure 1** with DBMPy (0.063 g, 0.3 mmol, 1 equiv.) and $AlCl_3$ (0.099 g, 0.75 mmol, 2.5 equiv.) in 3 mL chlorobenzene. Dichloropyrazabole (0.068 g, 0.3 mmol, 1 equiv.) was added to $AlCl_3$ (0.078 g, 0.6 mmol, 2 equiv.) in a seperate J. Young's Tap ampule and stirred at room temperature for 30 minutes until everything dissolved in chlorobenzene (2 mL). This reaction mixture was then transferred to the aluminated species (**1**) and the mixture heated for 4 h at 100 °C. After returning to room temperature, NEt_3 (0.2 mL) was added and the mixture stirred for 5 minutes. The ampule then was heated at 60 °C overnight under vacuum (ca 1 x 10^{-2} mbar). The residue was redissolved

in chlorobenzene (3 mL). 15 equiv. of NEt_3 (0.62 mL, 4.5 mmol 4.5 equiv.) and pinacol (0.532 g, 4.5 mmol, 4.5 equiv.) were added and the reaction heated for 4 h at 60 °C. The product was extracted with pentane, washed with K_2CO_3 (aq.) and dried over $MgSO_4$. An NMR spectrum of the crude after rotary evaporation gave a yield of 58% (vs internal standard: trimethylbenzene (0.1 mmol)). The borylated product can be isolated via flash column chromatography (100% PET – 100 % ETA), however only small amounts of **2** was recovered as a colourless oil due to its sensitivity to protodeboronation.

^{1}H NMR (400 MHz, $CDCl_3$) δ 6.95 (m, 1H), 6.89 (m, 1H), 2.30 (s, 3H), 1.44 (s, 6H), 1.37 (s, 2H), 1.35 (s, 9H), 1.15 (s, 12H); **^{11}B NMR (128 MHz, $CDCl_3$)** δ 33.5 (s); **^{11}B{^{1}H} NMR (128 MHz, $CDCl_3$)** δ 33.5 (s); **^{13}C{^{1}H} NMR** (126 MHz, $CDCl_3$) δ 168.06, 167.30, 146.22, 116.53, 116.09, 82.59, 39.11, 37.51, 30.34, 30.14, 26.56 (br s), 24.86, 21.55.

HRMS (ESI$^+$) *m/z* calcd for $C_{20}H_{35}BNO_2$: 332.2755 $[M+H]^+$, found 332.2765.

6.3. Synthesis of the C(sp^3)−H iodinated DBMPy (3)

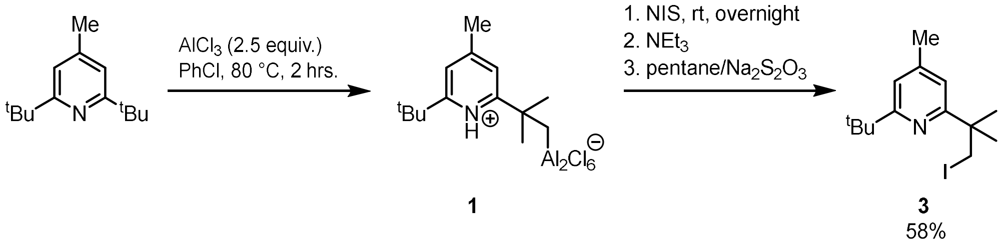

Compound **1** (0.3 mmol) is prepared according to **General Procedure 1** (2.5 equiv. $AlCl_3$, 0.25 mmol, 33 mg). NIS (2 equiv, 0.6 mmol, 132 mg) then was added and the reaction stirred at room temperature overnight. The reaction mixture was extracted with pentane and washed with $Na_2S_2O_3$ (aq.) and an internal yield determined after this partial work up vs an internal standard (0.204 mmol, 68%). The crude product was purified via flash column chromatography (100% PET). The title compound **3** was recovered as a colourless oil (58 mg, 0.174 mmol, 58%).

^{1}H NMR (500 MHz, $CDCl_3$) δ 6.96 (m, 1H), 6.88 (m, 1H), 3.69 (s, 2H), 2.33 (m, 3H), 1.47 (s, 6H), 1.34 (s, 9H); **^{13}C{^{1}H} NMR (126 MHz, $CDCl_3$)** δ 167.78, 162.86, 146.79, 116.99, 116.93, 40.81, 37.28, 30.01, 27.59, 23.93, 21.43.

Mass Spectrum: HRMS (ESI+) m/z calculated for $C_{14}H_{22}IN$: 332.0870; found: 332.0864.

6.4. Synthesis of the C(sp^3)−H brominated DBMPy (4)

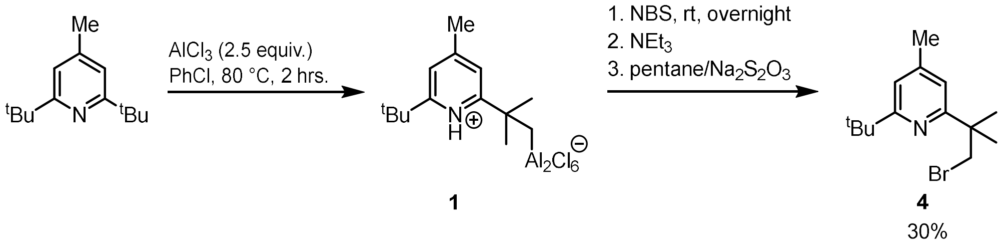

Compound **1** (0.3 mmol) was prepared according to **General Procedure 1** (1.2 equiv. $AlCl_3$, 0.25 mmol, 17 mg). NBS (2 equiv, 0.6 mmol, 132 mg) then was added and the reaction stirred at room temperature overnight. The reaction mixture was extracted with pentane and washed with $Na_2S_2O_3$ (aq.) and the internal yield determined after this partial work up vs an internal standard (0.126 mmol, 42%). The crude was purified via flash column chromatography (100% PET). The title compound **4** was isolated as a colourless oil (58 mg, 0.174 mmol, 30%).

^{1}H NMR (500 MHz, CDCl$_3$, 300 K) δ 6.96 (m, 1H), 6.91 (m, 1H), 3.83 (s, 2H), 2.33 (m, 3H), 1.45 (s, 6H), 1.33 (s, 9H); **^{13}C{^{1}H} NMR (126 MHz, CDCl$_3$)** δ 167.78, 162.86, 146.79, 116.99, 116.93, 40.81, 37.28, 30.01, 27.59, 23.93, 21.43.

Mass Spectrum: HRMS (EI+) m/z calculated for $C_{14}H_{22}BrN$: 283.09301; found: 283.09316

6.5. Synthesis of the C(sp^3)−H functionalised, oxidised DBMPy (5)

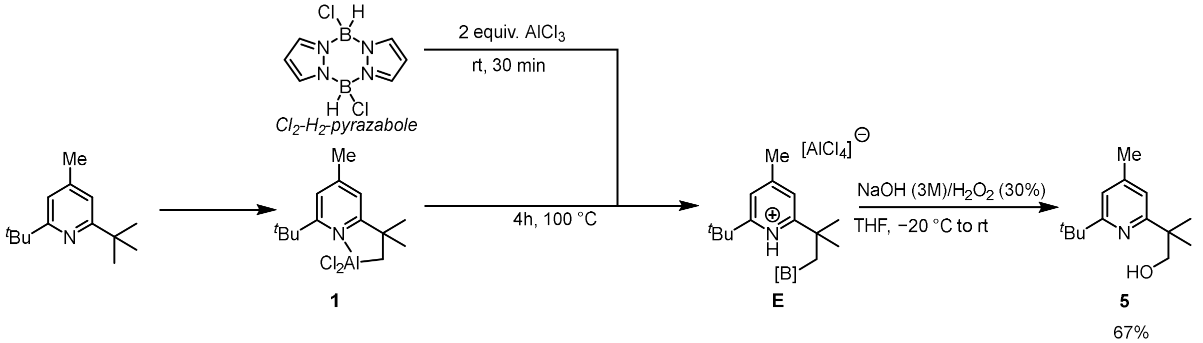

The mixture containing species **E** was prepared according to **General Procedure 2**. THF (3 ml) was added and the mixture cooled to −20 °C using an ice/NaCl bath. NaOH (3M, 5 mL) and H_2O_2 (2.5 mL) were added and the reaction was warmed to room temperature and stirred overnight. The crude was extracted with diethyl ether, washed with K_2CO_3 (aq.), dried over $MgSO_4$, filtered and the combined organic phases were concentrated *in vacuo*. The alcohol product was isolated via flash column chromatography (PET/ETA). The product was isolated as a colourless oil (29 mg, 0.129 mmol, 67%).

^{1}H NMR (500 MHz, $CDCl_3$) δ 7.00 (s, 1H), 6.90 (s, 1H), 6.07 (br s, 1H, *OH*), 3.71 (s, 2H), 2.32 (m, 3H), 1.33 (s, 9H), 1.29 (s, 6H). **$^{13}C\{^{1}H\}$ NMR (126 MHz, $CDCl_3$)** δ 167.28, 167.04, 148.29, 118.06, 117.85, 72.48, 40.52, 37.26, 30.32, 25.87, 21.68.

HRMS (ESI^+) *m/z* calcd for $C_{14}H_{24}NO$: 222.1852 $[M+H]^+$, found 222.1849.

7. NMR Spectra of functionalised DBMPy

NMR Spectra of (**2**):

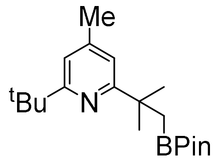

2

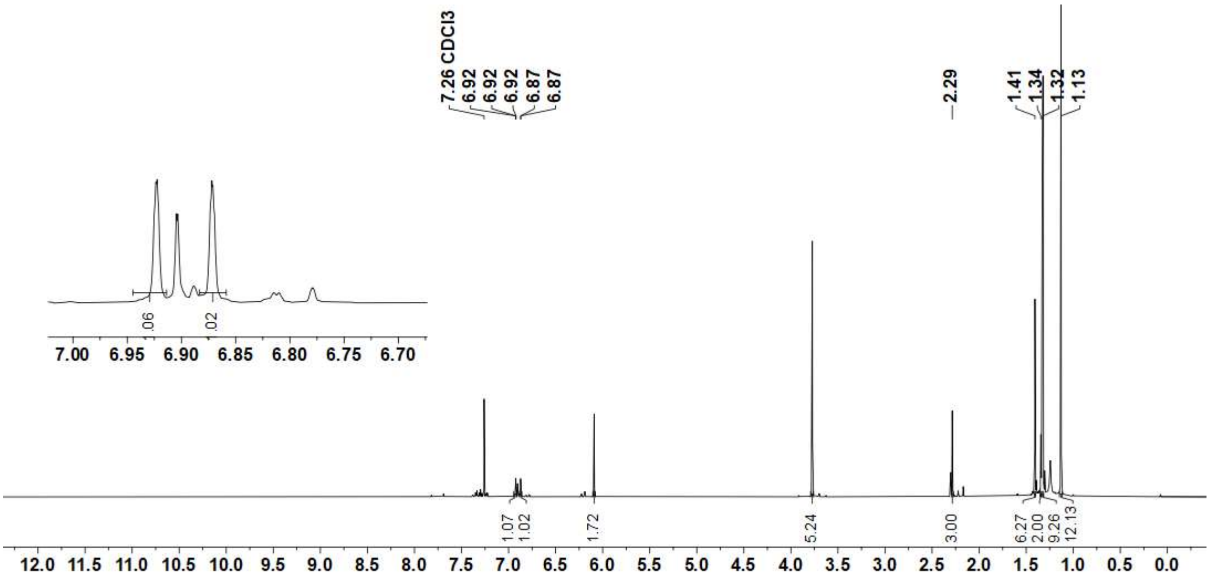

Figure S29: ^{1}H NMR spectrum of crude (**2**) in $CDCl_3$ with internal standard (trimethoxybenzene). *Note: impurity resonances at ca. 7.30 ppm are due to trace chlorobenzene and those at 7.50-7.70 ppm and 6.15-6.30 ppm correspond to remaining pyrazole derivatives.*

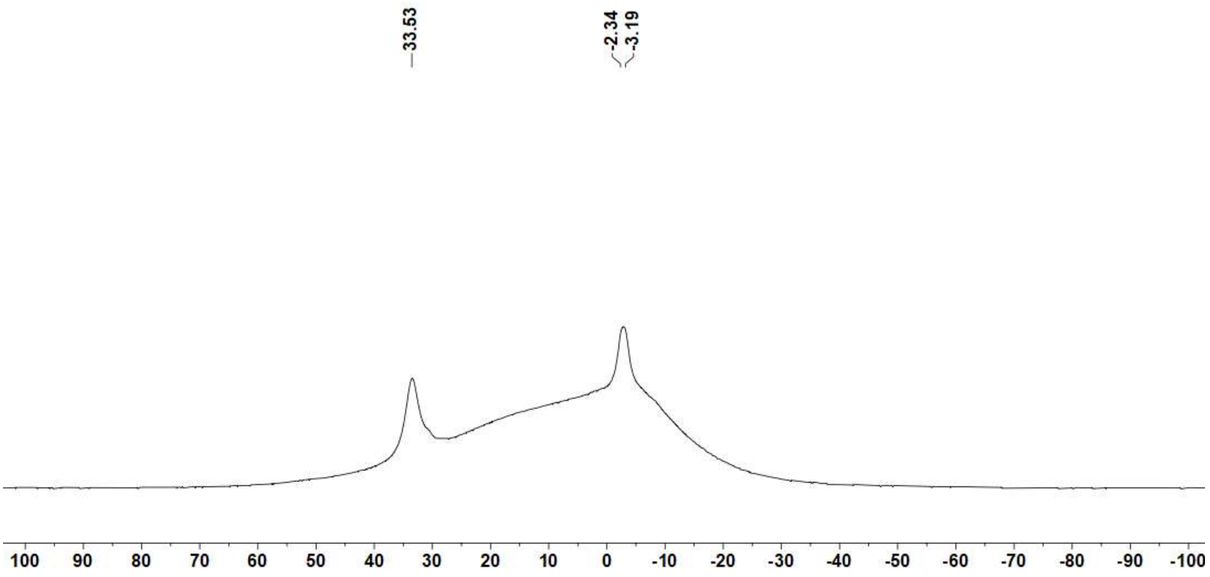

Figure S30: ^{11}B NMR spectrum of crude (**2**) in $CDCl_3$. *Note: impurity resonances at −2.34 and −3.19 ppm correspond to pyrazabole derivatives.*

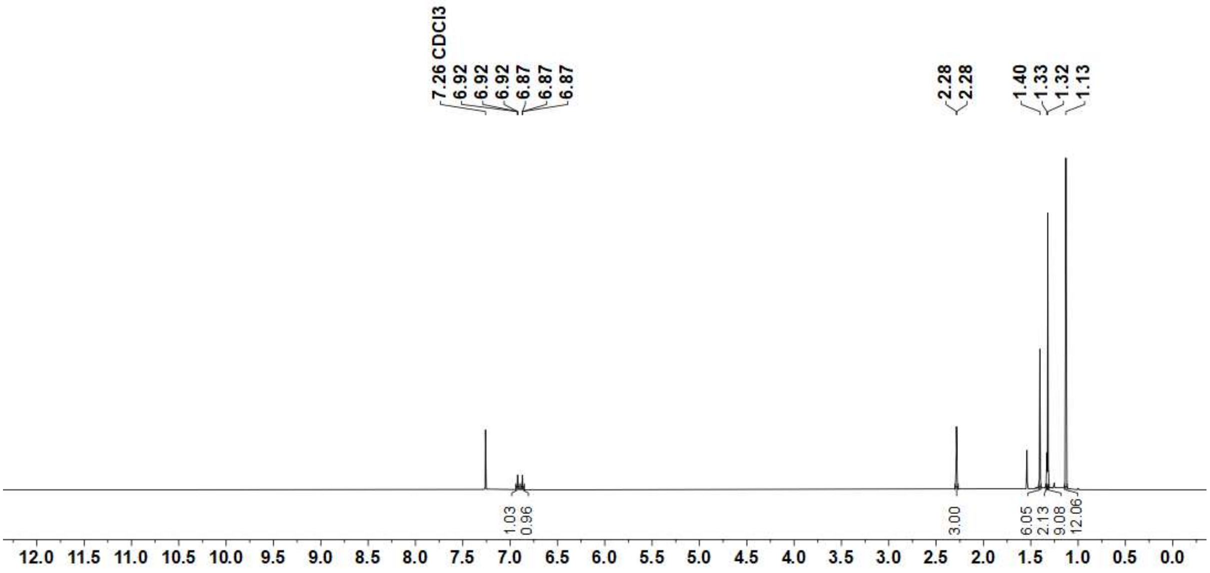

Figure S31: ^{1}H NMR spectrum of (**2**) in $CDCl_3$ after isolation via flash column chromatography.

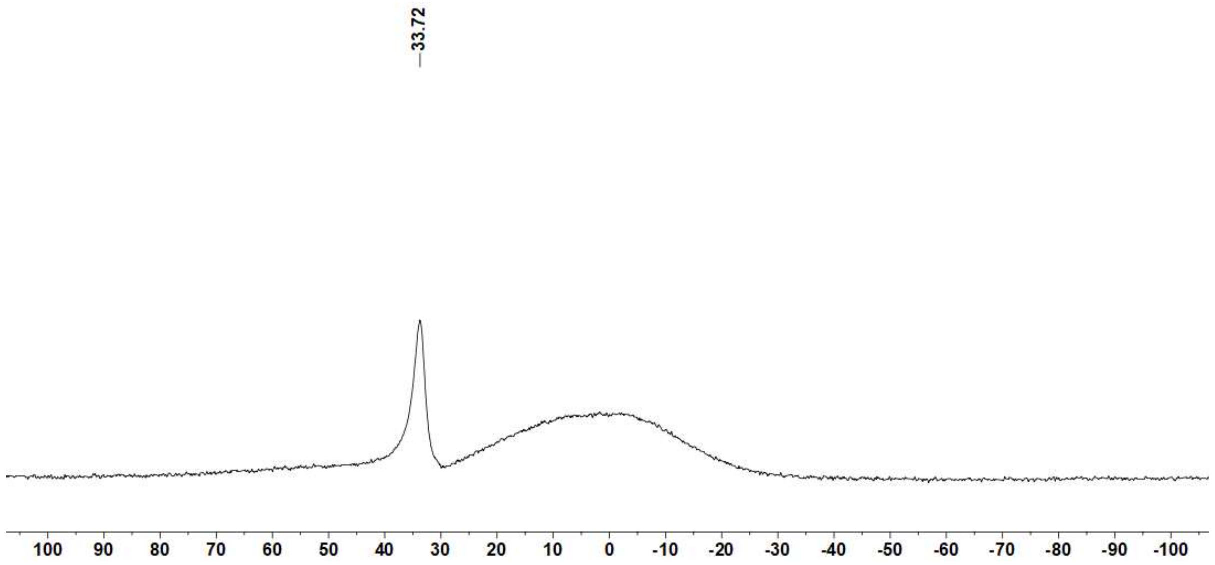

Figure S32: ^{11}B NMR spectrum of (**2**) in $CDCl_3$ after isolation via flash column chromatography.

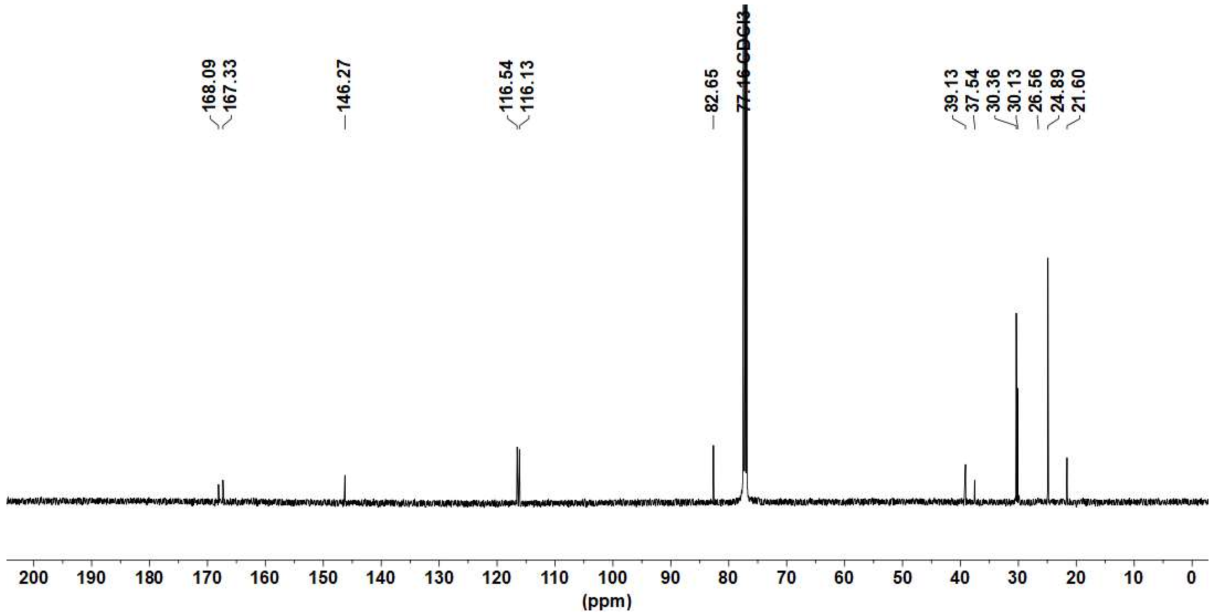

Figure S33: $^{13}C\{^{1}H\}$ NMR spectrum of (**2**) in $CDCl_3$ after isolation via flash column chromatography.

NMR Spectra of (**3**):

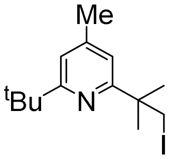

3

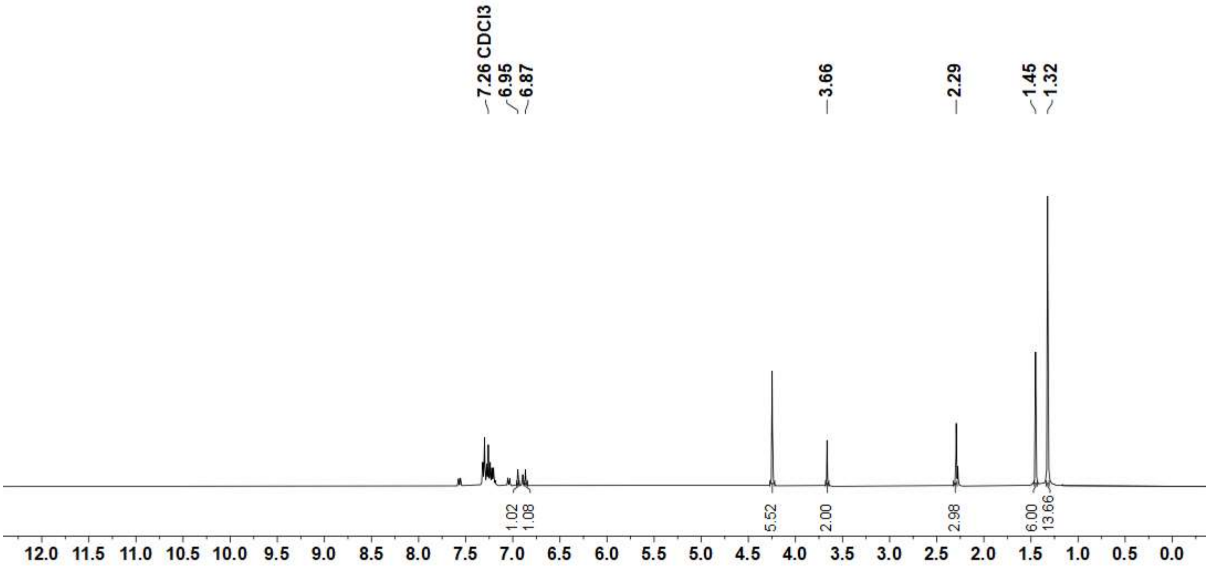

Figure S34: ^{1}H NMR spectrum of crude of (**3**) with internal standard (nitromethane) in $CDCl_3$. *Note: Impurity resonances at ca. 7.30 ppm correspond to chlorobenzene.*

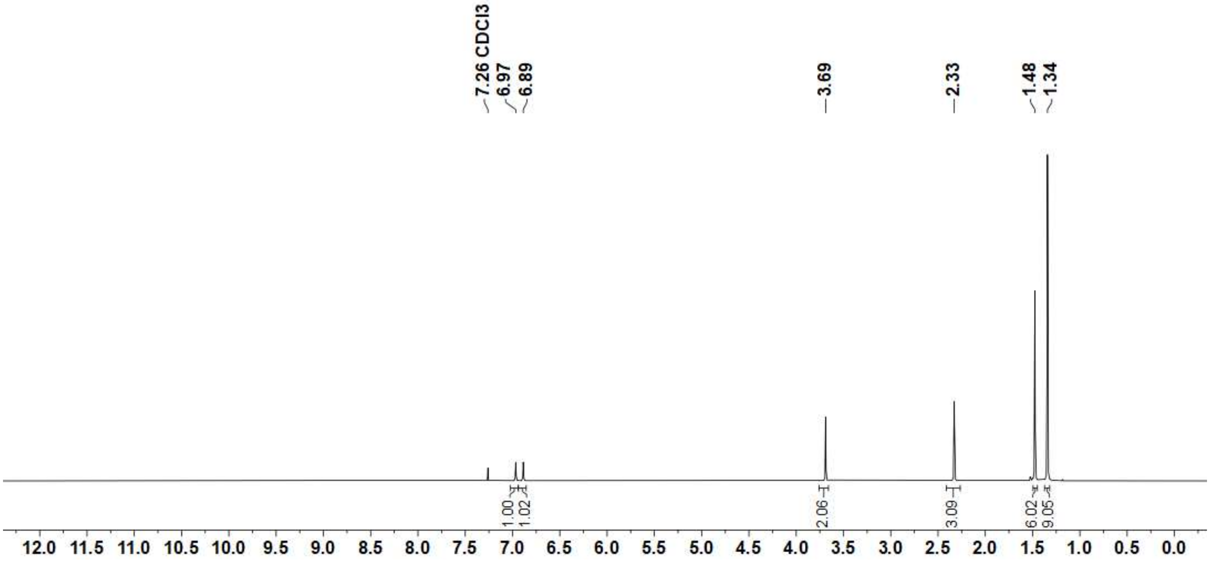

Figure S35: ^{1}H NMR spectrum of isolated (**3**) in $CDCl_3$ after flash column chromatography.

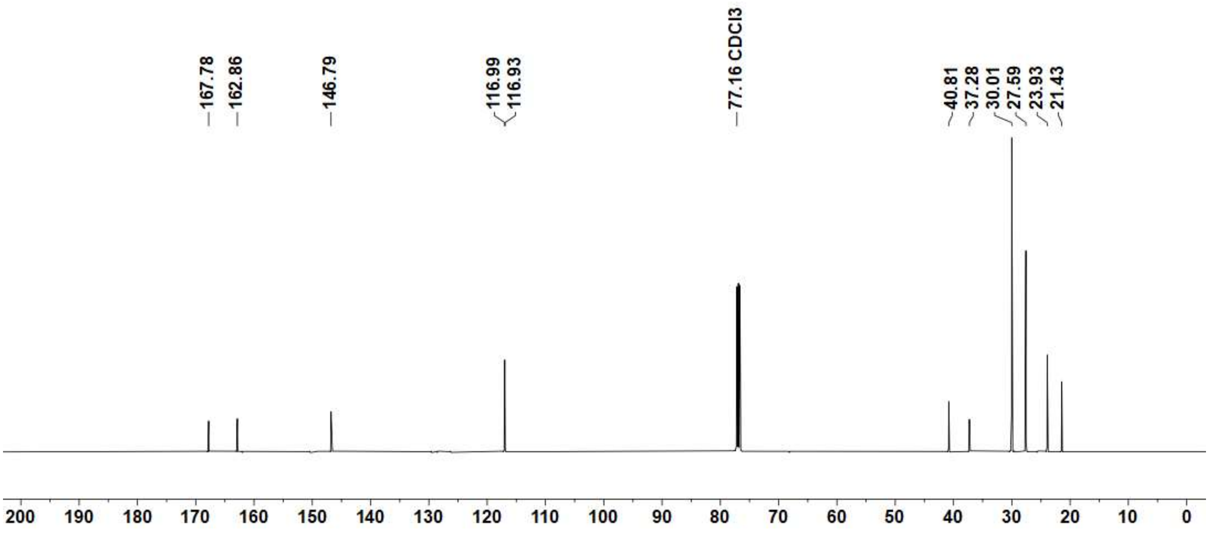

Figure S36: $^{13}C\{^1H\}$ NMR spectrum of isolated (**3**) in $CDCl_3$ after flash column chromatography.

<u>NMR Spectra of (**4**):</u>

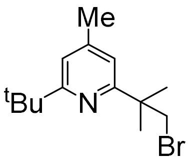

4

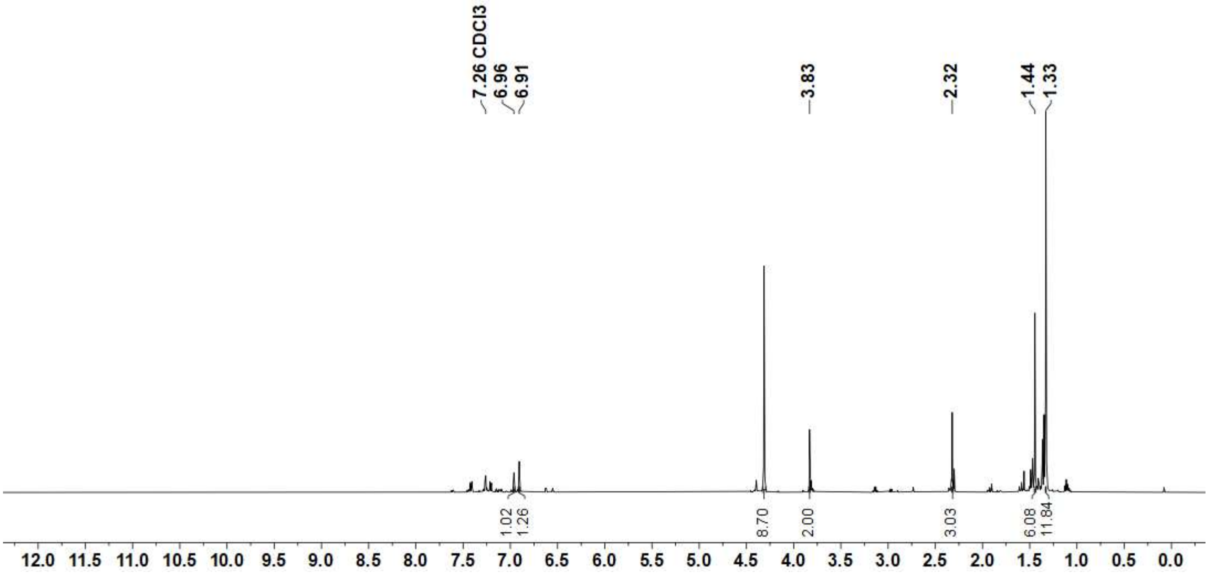

Figure S37: ^1H NMR spectrum of crude (**4**) with internal standard (nitromethane) in $CDCl_3$.

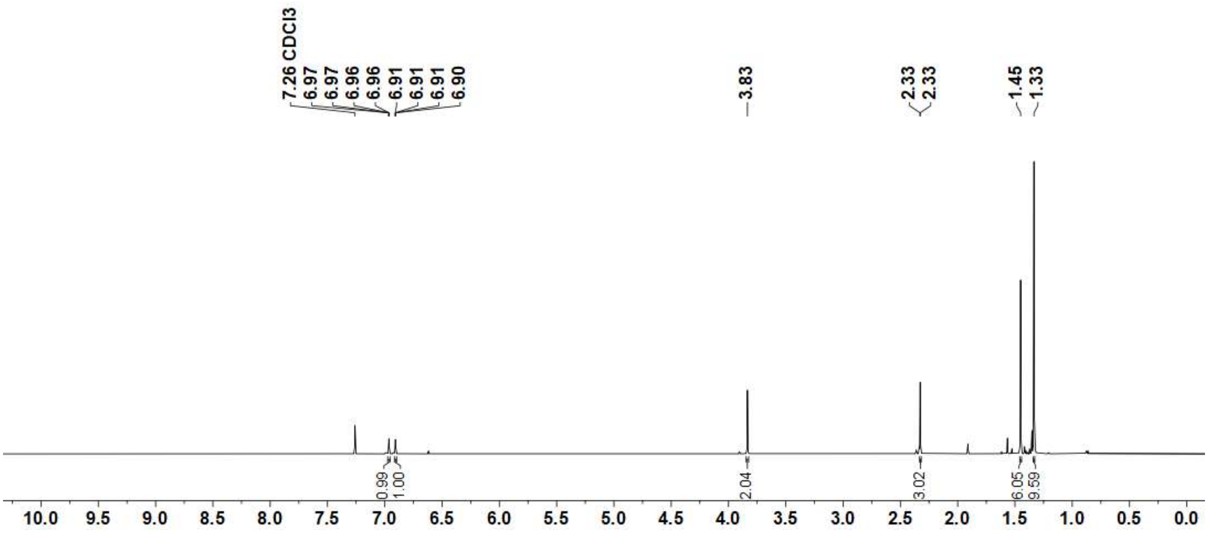

Figure S38: ^{1}H NMR spectrum of (**4**) in $CDCl_3$ after isolation via flash column chromatography.

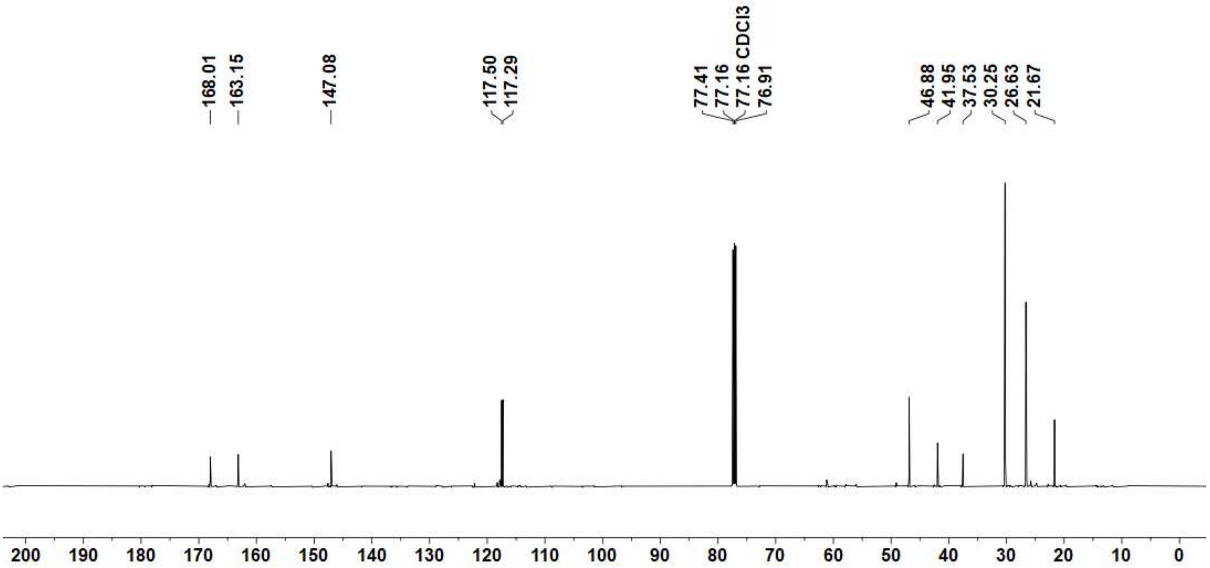

Figure S39: $^{13}C\{^{1}H\}$ NMR spectrum of (**4**) in $CDCl_3$ after isolation via flash column chromatography.

NMR Spectra of (**5**):

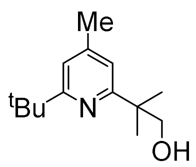

5

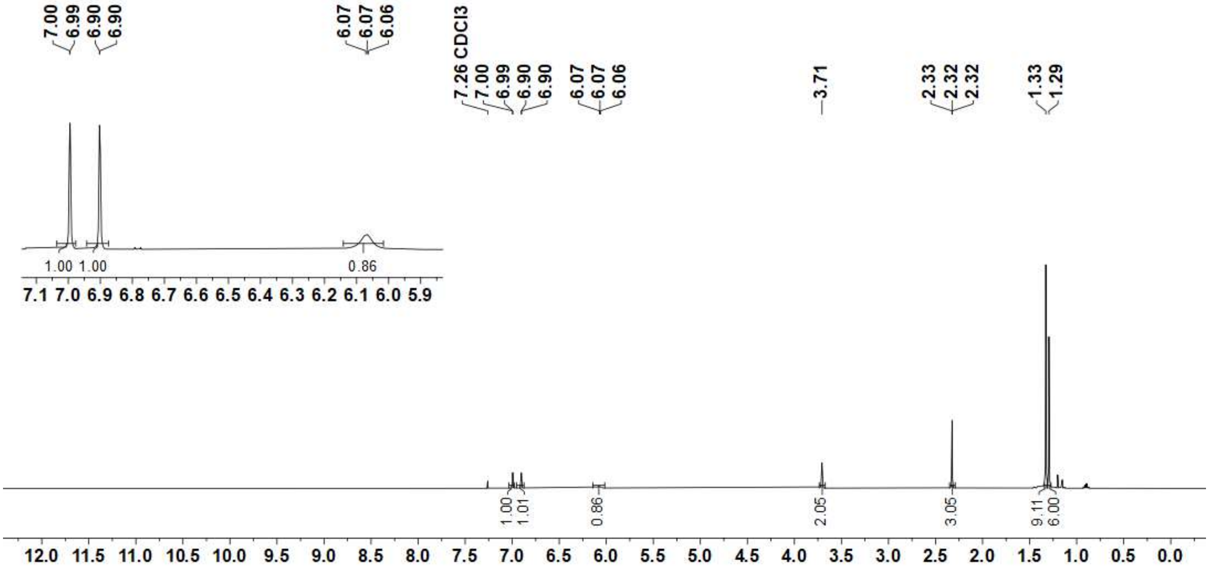

Figure S40: ^{1}H NMR spectrum of (**5**) in $CDCl_3$.

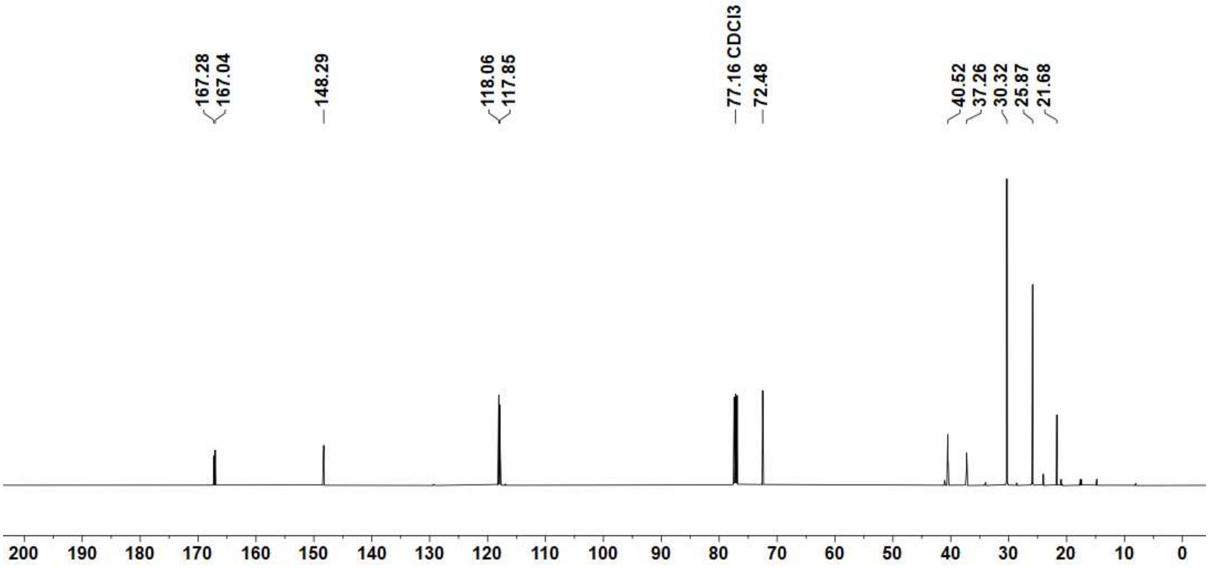

Figure S41: $^{13}C\{^{1}H\}$ NMR spectrum of (**5**) in $CDCl_3$.

8. Alumination of other substrates

Note, to enable in-situ characterisation of a number of aluminated products the corresponding protonated species [(Base)H][$AlCl_4$] were synthesised.

Synthesis of protonated bases: The respective substrate (1 equiv.) was added to a Young's Tap NMR tube with (1.2 equiv.) $AlCl_3$ and then PhCl was added (0.5 mL). HCl (1 M in diethylether, 1 equiv.) was added and the reaction sealed and rotated for 5 minutes. All solvents then were removed *in vacuo*, before the residue was redissolved in PhCl/$CDCl_3$, remaining solids removed by filtration and NMR spectra recorded in the respective solvent.

8.1. Synthesis of the C(sp^3)−H aluminated and iodinated (9)

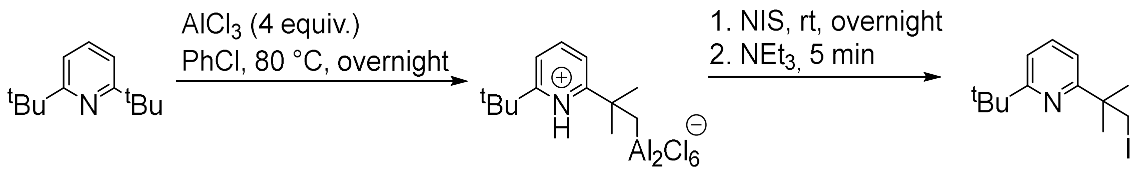

9 77%   **3-p-H** 38%

Compound **9** is prepared according to **General Procedure 1** with $AlCl_3$ (0.053 g, 0.4 mmol, 4.0 equiv.) at 80 °C overnight. The metalated product is obtained in 77% yield vs internal standard (0.1 mmol).

^{1}H NMR (500 MHz, PhCl) δ 10.85 (br s, 1H), 7.75, 1.44 (s, 6H), 1.17 (s, 9H), 1.15 (s, 2H). **^{13}C{^{1}H} NMR (126 MHz, PhCl)** δ 163.74, 161.67, 146.69, 136.63, 120.97, 38.50, 35.68, 28.36 (bs), 27.64, 27.61. (*Note: Some aryl−H resonances in the ^{1}H NMR spectrum are covered by chlorobenzene and multiplicities or integrations can not be determined).* **^{27}Al NMR** (130 MHz, PhCl) δ not observed.

^{1}H NMR (500 MHz, $CDCl_3$) δ 8.56 (t, J = 8.1 Hz, 1H), 8.45 (t, J = 8.1 Hz, 1H), 7.90 (dd, J = 8.1, 2.0 Hz, 1H), 1.60 (s, 9H), 1.60 (s, 6H), 1.38 (s, 2H). **^{27}Al NMR** (130 MHz, PhCl) δ not observed.

Compound **9** was further reacted with NIS (0.044 g, 0.20 mmol, 2 equiv.) at room temperature overnight. NEt_3 (0.5 mL) was added to the reaction and stirred for 5 minutes. The iodinated

product is extracted with PET (3x5 mL) and washed with $Na_2S_2O_3$ (aq.) (3x5 mL). The product is purified via flash column chromatography (100% PET) and obtained as a colourless oil (38%, 12 mg).

^{1}H NMR (500 MHz, $CDCl_3$) δ 7.54 (t, *J* = 7.8 Hz, 1H), 7.14 (dd, *J* = 7.9, 0.7 Hz, 1H), 7.06 (dd, *J* = 7.8, 0.7 Hz, 1H), 3.68 (s, 2H), 1.49 (s, 6H), 1.35 (s, 9H). **^{13}C{^{1}H} NMR (126 MHz, $CDCl_3$)** δ 168.22, 163.25, 136.43, 116.31, 116.19, 41.25, 37.74, 30.25, 27.80, 23.98.

HRMS (ESI$^+$) *m/z* calcd for $C_{13}H_{21}IN$: 318.07132 $[M+H]^+$, found 318.0705.

8.2. Synthesis of the C(sp^3)−H aluminated (10)

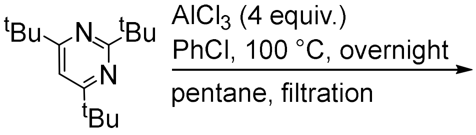

10

Compound **10** was prepared following **General Procedure 1** with $AlCl_3$ (0.053 g, 0.4 mmol, 4 equiv.) at 100 °C overnight followed by 5h at 120 °C. The product was obtained in 33% yield vs internal standard. Approximately 80% of solvent was removed *in vacuo* and 1 mL of pentane was added. The title compound is soluble in pentane and was filtered off. **10** was characterised in $CDCl_3$.

^{1}H NMR (500 MHz, PhCl) δ 7.39 (s), 1.41 (s, 6H), 1.39 (s, 9H), 1.23 (s, 9H), 0.91 (s, 2H). **^{13}C{^{1}H} NMR (126 MHz, PhCl)** δ 185.64, 181.88, 178.20, 112.68, 43.44, 38.92, 38.72, 33.14, 30.39, 28.49, 21.26 (br s). *(Note: Some aryl−H resonances in the ^{1}H NMR spectrum are covered by chlorobenzene and multiplicities or integrations can not be determined).*

^{1}H NMR (500 MHz, $CDCl_3$) δ 7.47 (s, 1H), 1.65 (s, 9H), 1.50 (s, 6H), 1.40 (s, 9H), 0.60 (s, 2H). **^{13}C{^{1}H} NMR (126 MHz, $CDCl_3$)** δ 184.21, 182.21, 178.49, 112.44, 43.95, 39.54, 39.25, 33.90, 31.37, 29.37, 22.25 (br s, not detected in ^{13}C{^{1}H} NMR (but confirmed by ^{1}H-^{13}C{^{1}H} HSQC)).

The crude aluminated **10** in PhCl was functionalised *in situ* by addition of NIS (2 equiv., 0.2 mmol, 44 mg) and stirring overnight at room temperature. NEt_3 (0.2 mL) was added and the organic phase was extracted using pentane (2x20 mL), washed with $Na_2S_2O_3$ (aq.) (3x20 mL), dried over $NaSO_4$, filtrated and concentrated *in vacuo*. The crude was purified with silica flash column chromatography (100% PET) to give the iodinated product in 28% yield (0.011 g).

^{1}H NMR (500 MHz, $CDCl_3$)) δ 7.07 (s, 1H), 3.99 (s, 2H), 1.45 (s, 6H), 1.32 (s, 18H).**^{13}C{^{1}H} NMR (126 MHz, $CDCl_3$)** δ 177.13, 171.50, 108.15, 55.48, 44.67, 37.83, 29.66, 25.50.

HRMS (ESI$^+$) *m/z* calcd for $C_{16}H_{28}IN$: 375.1292 [M+H]$^+$, found 375.1294.

8.5. Synthesis of the C(sp^3)−H aluminated and iodinated (11)

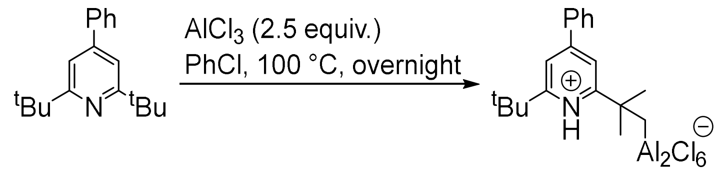

Note as received 2,6-*t*Bu-4-phenylpyridine was stored in DCM over mol sieves (3Å) overnight to remove any water. The substrate was then dried *in vacuo* and used directly in the reaction. Compound **11** was prepared according to **General Procedure 1** with $AlCl_3$ (0.033 g, 0.25 mmol, 2.5 equiv) at 100 °C overnight. The product is obtained in 84% yield (vs internal standard). The product was not separated from the protonated starting material impurity.

^{1}H NMR (500 MHz, PhCl) δ 10.68 (br s, 1H), 7.71 (m, 1H), 1.53 (s, 6H), 1.26 (s, 9H), 1.25 (s, 2H). **^{13}C{H} NMR (126 MHz, PhCl)** δ 164.24, 162.11, 159.82, 132.34, 128.19, 119.47, 119.00, 39.24, 36.42, 30.28, 29.53, 26.98. *(Note: Some aryl−H resonances in the ^{1}H and ^{13}C{H} NMR spectrum are covered by chlorobenzene).*

^{1}H NMR (500 MHz, $CDCl_3$) δ 10.95 (s, 1H), 7.94 (t, *J* = 1.6 Hz, 1H), 7.91 (d, *J* = 1.8 Hz, 1H), 7.83 (t, *J* = 1.7 Hz, 1H), 7.77 – 7.74 (m, 3H), 7.66 – 7.63 (m, 3H), 7.62 (s, 1H), 1.72 (s, 6H), 1.63 (s, 9H), 1.19 (s, 2H). *Note: Aromatic peaks of the title compound overlap with the protonated byproduct (alkyl group: 1.64).*

Compound **11** was further reacted with NIS (0.044 g, 0.20 mmol, 2 equiv.) at room temperature overnight. NEt_3 (0.5 mL) was added to the reaction and stirred for 5 minutes. The iodinated product is extracted with PET (3x5 mL) and washed with $Na_2S_2O_3$ (aq.) (3x5 mL). The product is purified via flash column chromatography (100% PET) and obtained as a colourless oil (31%).

^{1}H NMR (500 MHz, $CDCl_3$) δ 7.60 (d, *J* = 7.0 Hz, 2H), 7.47 – 7.42 (m, 2H), 7.40 (d, *J* = 7.1 Hz, 1H), 7.32 (d, *J* = 1.3 Hz, 1H), 7.24 – 7.23 (m, 1H), 3.70 (s, 2H), 1.52 (s, 6H), 1.38 (s, 9H).

^{13}C{^{1}H} NMR (126 MHz, $CDCl_3$) δ ^{13}C NMR (151 MHz, $CDCl_3$) δ 168.75, 163.83, 149.11, 140.09, 129.06, 128.62, 127.42, 114.79, 114.78, 41.37, 37.87, 30.33, 27.90, 23.93.

HRMS (ESI$^+$) *m/z* calcd for $C_{19}H_{24}IN$: 394.1026 [M+H]$^+$, found 394.1025.

8.3. Synthesis of the C(sp^3)−H aluminated (12)

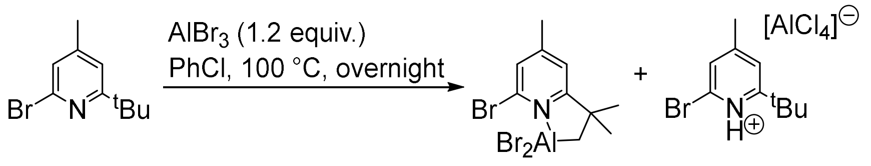

12

Note, 2-Br-4-methyl-6-*t*Bupyridine was stored in DCM over mol sieves (3 Å) overnight and then the substrate was then dried *in vacuo* and stored in the glovebox. Compound **12** was prepared according to **General Procedure 1** with $AlBr_3$ (0.017 g) with overnight heating at 100 °C. The product is obtained in 44% internal yield (vs internal standard, theoretical maximum = 50%). Approximately 80% of solvent was removed *in vacuo* and 1 mL of pentane was added. The protonated base by-product precipitates as a white solid. The title compound is soluble in pentane and was filtered off. The aluminated species was characterised again in $CDCl_3$.

^{1}H NMR (500 MHz, PhCl) δ 7.05, 1.91 (s, 3H), 1.19 (s, 6H), 0.96 (s, 2H). **^{13}C{^{1}H} NMR (126 MHz, PhCl)** δ 176.49, 171.74, 159.82, 138.19, 122.61, 42.33, 33.70, 24.73 (br s), 21.72. *(Note: Some aryl−H resonances in the ^{1}H NMR spectrum are covered by chlorobenzene and multiplicities or integrations can not be determined).* **^{27}Al NMR (130 MHz, PhCl)** δ not observed.

^{1}H NMR (500 MHz, $CDCl_3$) δ 7.49 (s, 1H), 7.44 (s, 1H), 2.66 (s, 3H), 1.57 (s, 6H), 0.73 (s, 2H). **^{13}C NMR (126 MHz, $CDCl_3$)** δ 176.84, 157.51, 138.49, 130.66, 121.96, 41.72, 33.78, 21.72, br s (Al−$\underline{C}H_2$) not observed.

8.1. Synthesis of the C(sp^3)−H aluminated (14)

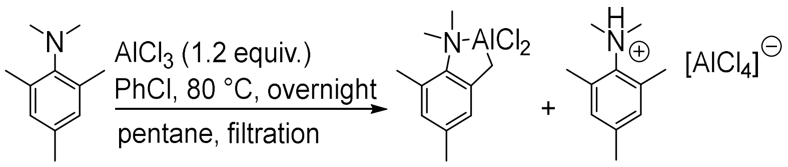

14

Compound **14** was prepared following **General Procedure 1** with $AlCl_3$ (0.017 g, 0.12 mmol, 1.2 equiv.) at 80 °C overnight. The product was obtained in 46% yield vs internal standard (max. theoretical yield is 50%). Note, this reaction gives the same outcome and yield when

left stirring without heating for 60 hrs. Approximately 80% of solvent was removed *in vacuo* and 1 mL of pentane was then added. The protonated by-product precipitates as a white solid. The title compound is soluble in pentane and was separated by filtration.

^{1}H NMR (500 MHz, PhCl) δ 6.74, 6.45, 2.62 (s, 6H), 2.02 (s, 3H), 2.00 (s, 3H), 1.58 (s, 2H). **^{13}C{^{1}H} (126 MHz, PhCl)** δ 141.14, 138.23, 138.18, 135.01, 132.38, 131.72, 45.78, 20.53, 20.28, 12.30 (br s). **^{27}Al NMR (130 MHz, PhCl)** δ not observed.

^{1}H NMR (500 MHz, $CDCl_3$) δ 7.02 (s, 1H), 6.76 (s, 1H), 3.09 (s, 6H), 2.48 (s, 3H), 2.25 (s, 3H), 1.56 (s, 2H). **^{13}C NMR (126 MHz, $CDCl_3$)** δ 141.89, 138.87, 138.26, 137.11, 132.05, 131.94, 46.84, 21.36, 19.12, 15.10. **^{27}Al NMR (130 MHz, $CDCl_3$)** δ not observed.

9. NMR Spectra of other aluminated Substrates

NMR Spectra of (**9**):

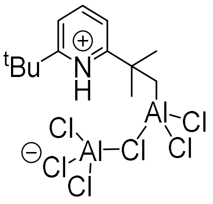

9

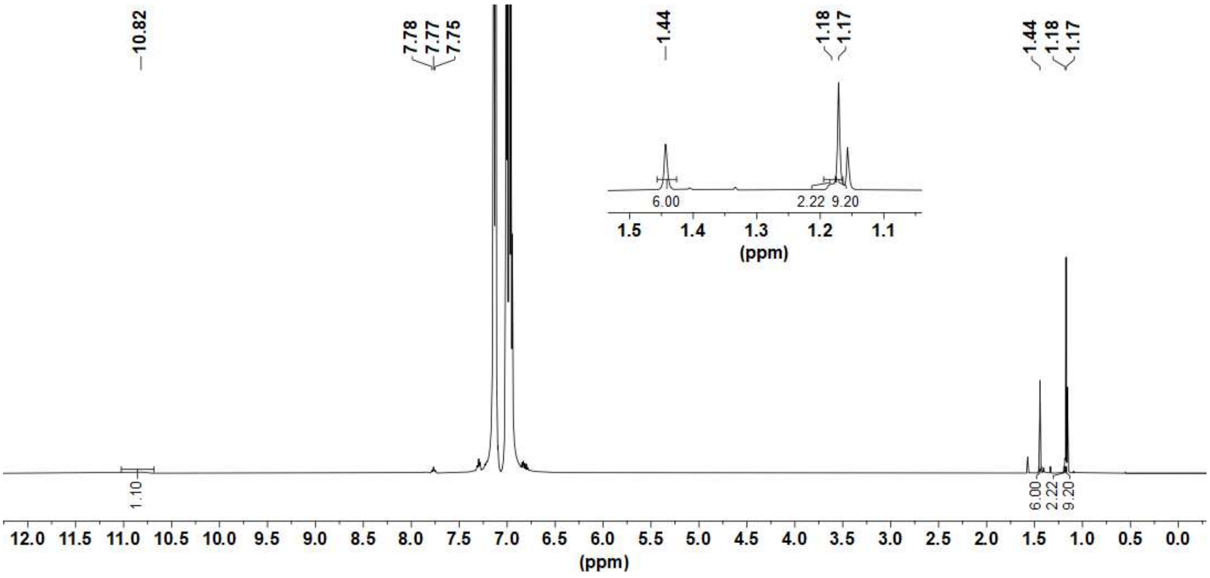

Figure S42: ^{1}H NMR spectrum of (**9**) in PhCl. *Note: resonance at 1.15 ppm is due to protonated 2,6-di-tert-butyl-pyridine* (see **Figure S50**).

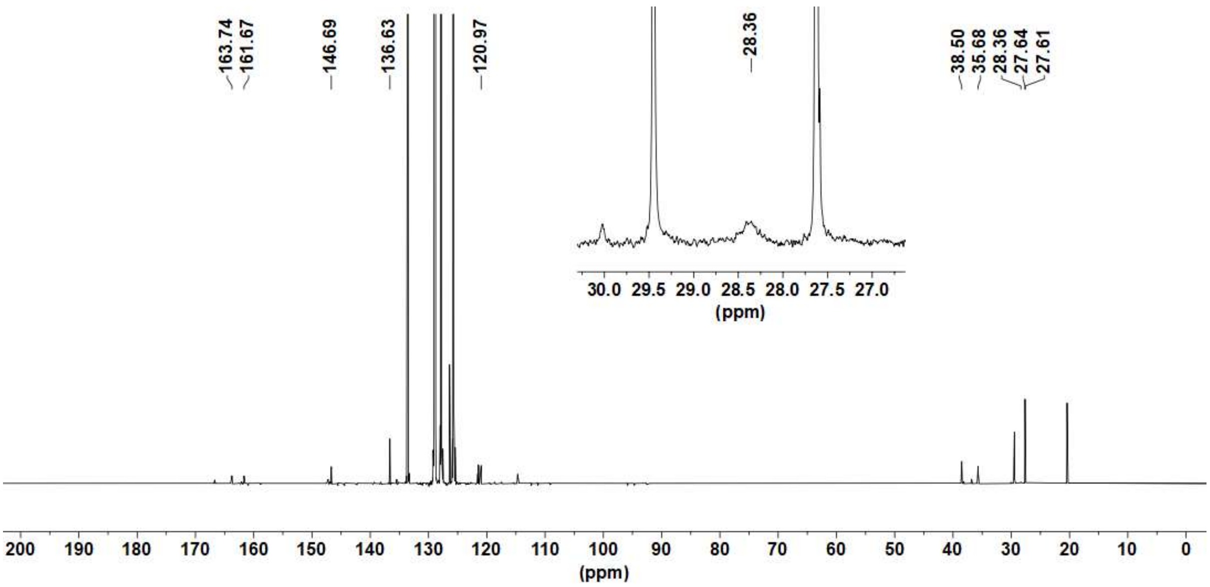

Figure S43: $^{13}C\{^1H\}$ NMR spectrum of **(9)** in PhCl. *Note: Other resonances are due to free 2,6-di-tert-butyl-pyridine. Every peak that is picked was assigned via ^1H-$^{13}C\{^1H\}$ HSQC NMR.*

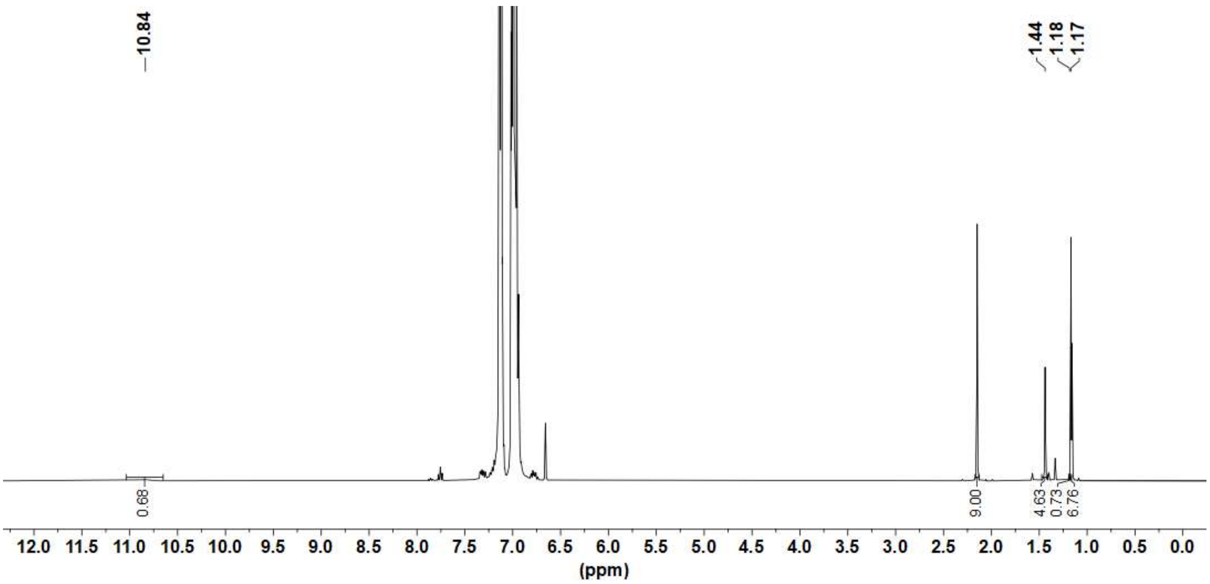

Figure S44: ^1H NMR spectrum of (**9**) in PhCl with internal standard (mesitylene). *Note: Other resonances are due to unreacted starting material and protonated 2,6-di-tert-butyl-pyridine (see* **Figure S50**).

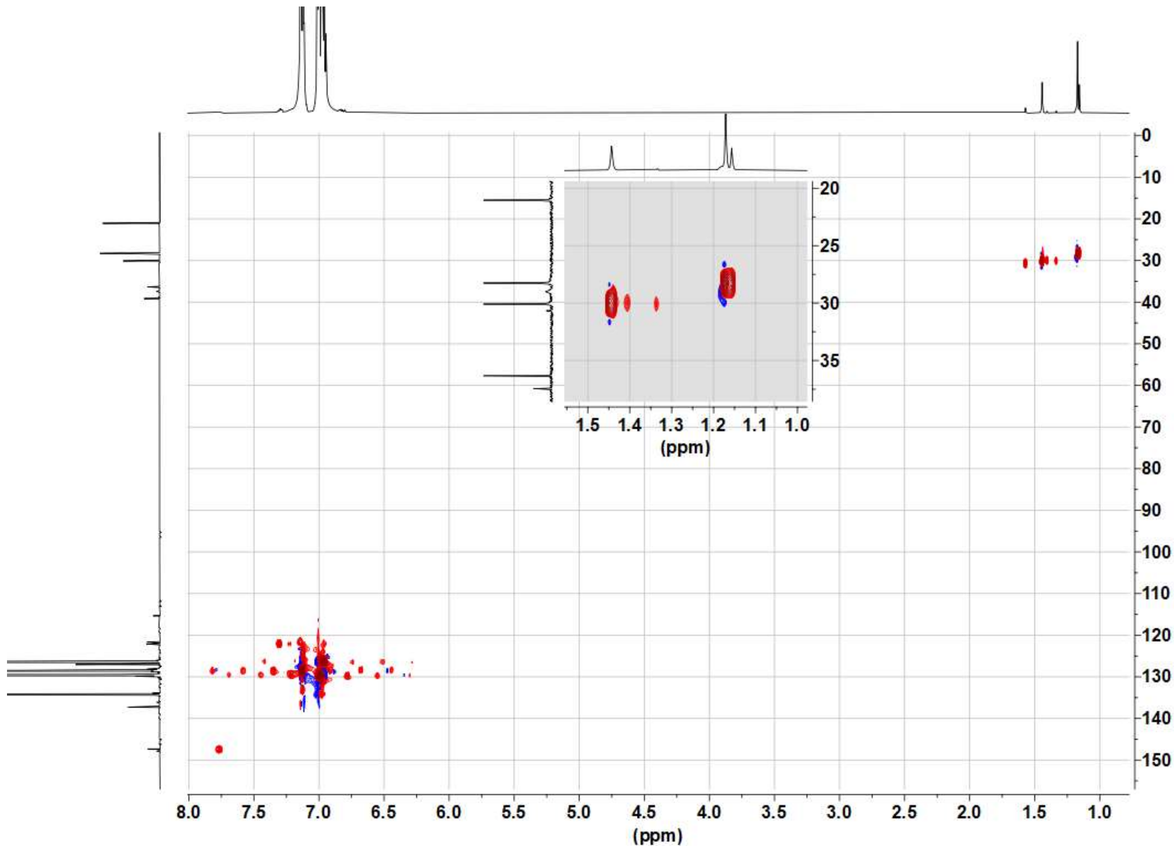

Figure S45: $^{1}H-^{13}C\{^{1}H\}$ HSQC NMR spectrum of (**9**) in PhCl. *Note: Other resonances are due to protonated 2,6-di-tert-butyl-pyridine (see* **Figure S50***).*

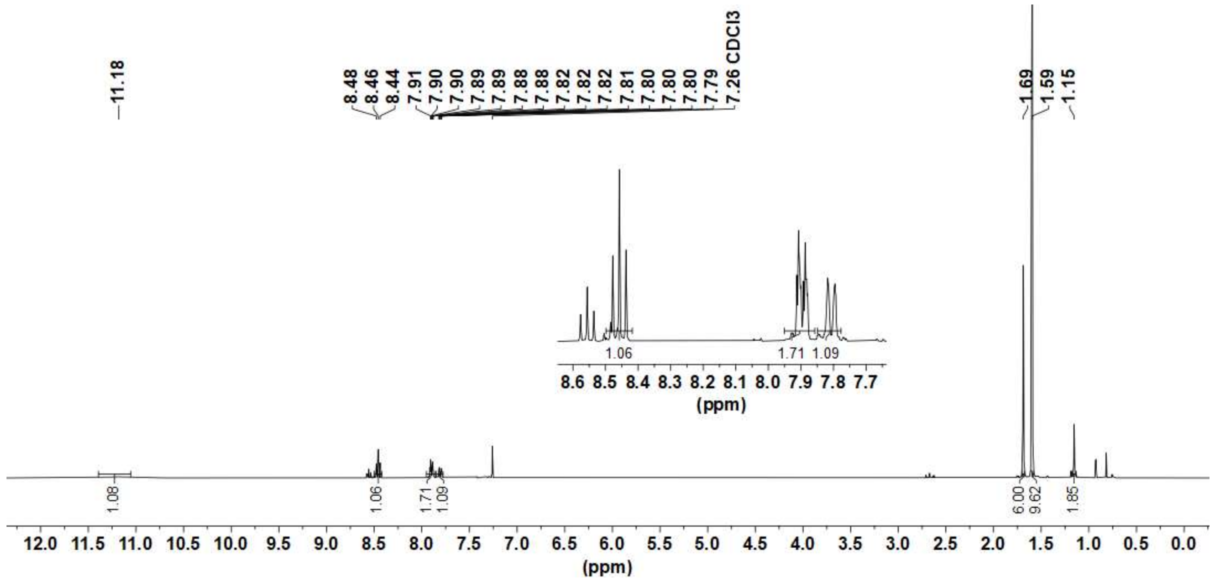

Figure S46: ^{1}H NMR spectrum of (**9**) in $CDCl_3$. *Note: Other resonances are due to protonated 2,6-di-tert-butyl-pyridine partially overlapping with the aryl−H resonances of the product (see* **Figure S51***).*

NMR Spectra of **(3-*p*-H):**

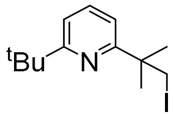

3-*p*-H

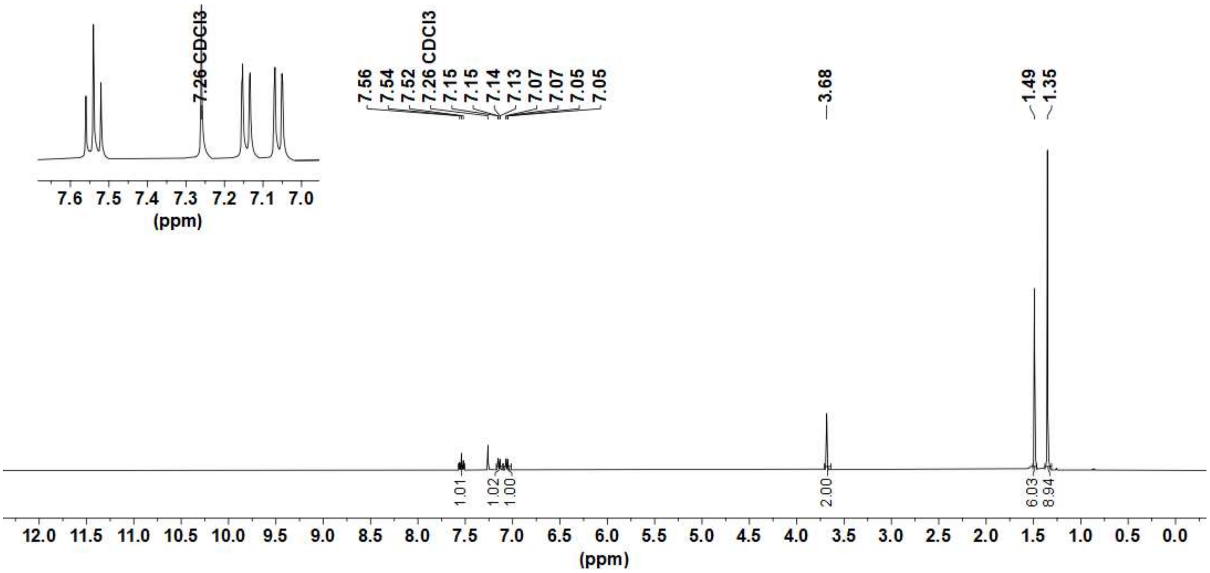

Figure S47: ^{1}H NMR spectrum of (**3-*p*-H**) in $CDCl_3$. *Note: Other resonances at ca. 7.30 ppm are due to chlorobenzene and at 1.53 ppm to water.*

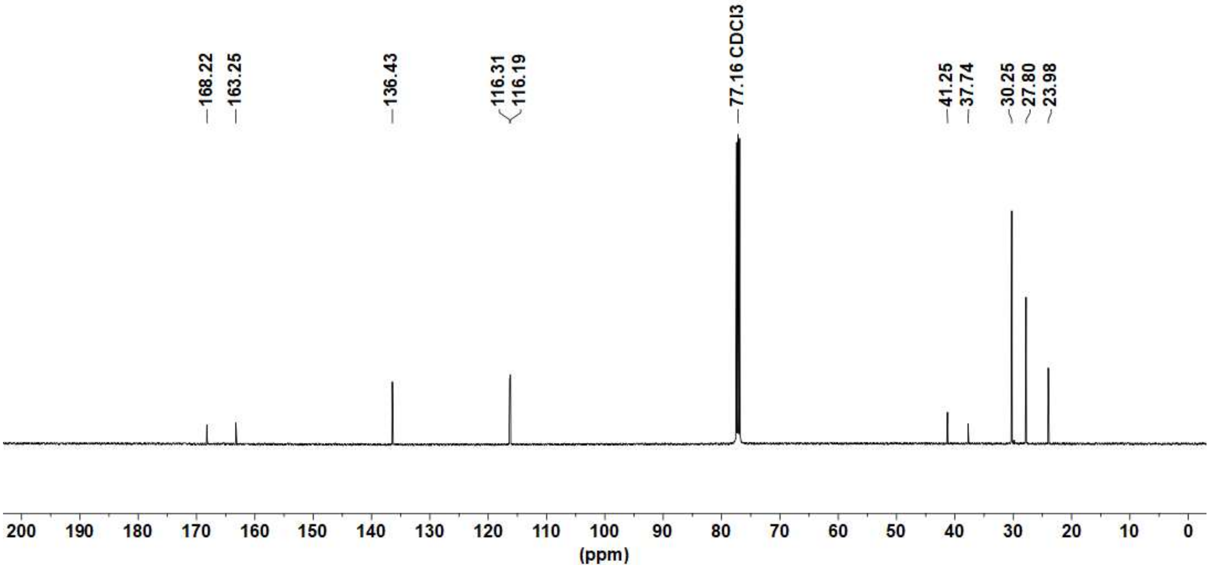

Figure S48: $^{13}C\{^{1}H\}$ NMR spectrum of (**3-*p*-H**) in $CDCl_3$.

NMR of 2,6-di-tert-butyl-pyridine:

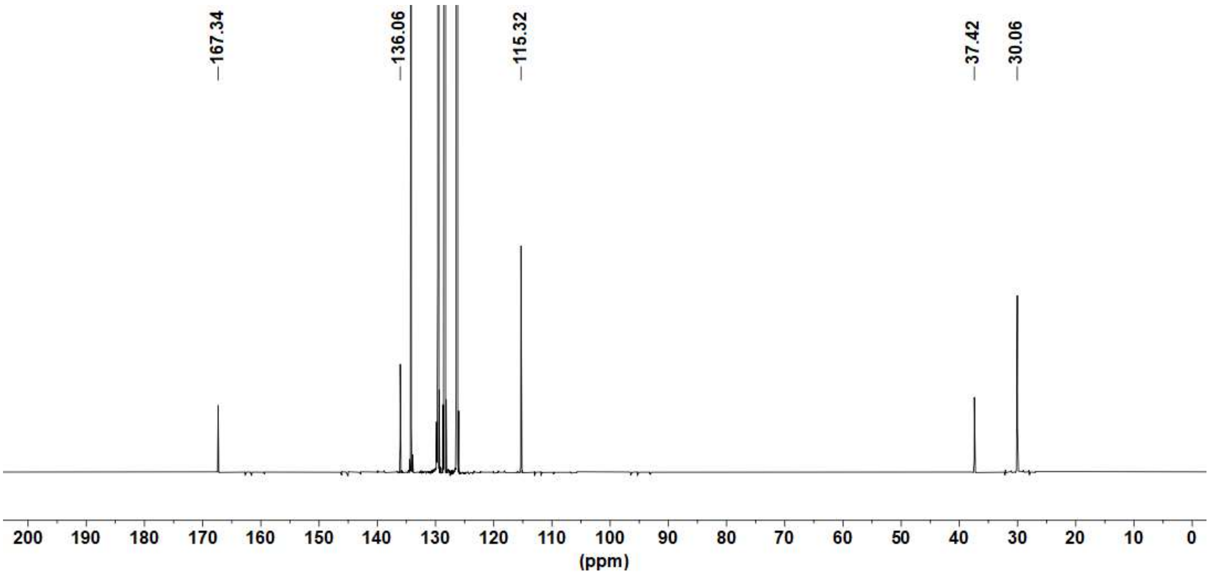

Figure S49: $^{13}C\{^{1}H\}$ NMR spectrum of protonated *2,6-di-tert-butyl-pyridine* in PhCl. *Note: impurity resonances at 3.99 and 1.06 ppm are due to diethylether.*

NMR of protonated 2,6-di-tert-butyl-pyridine[$AlCl_4$] salt:

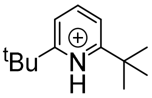

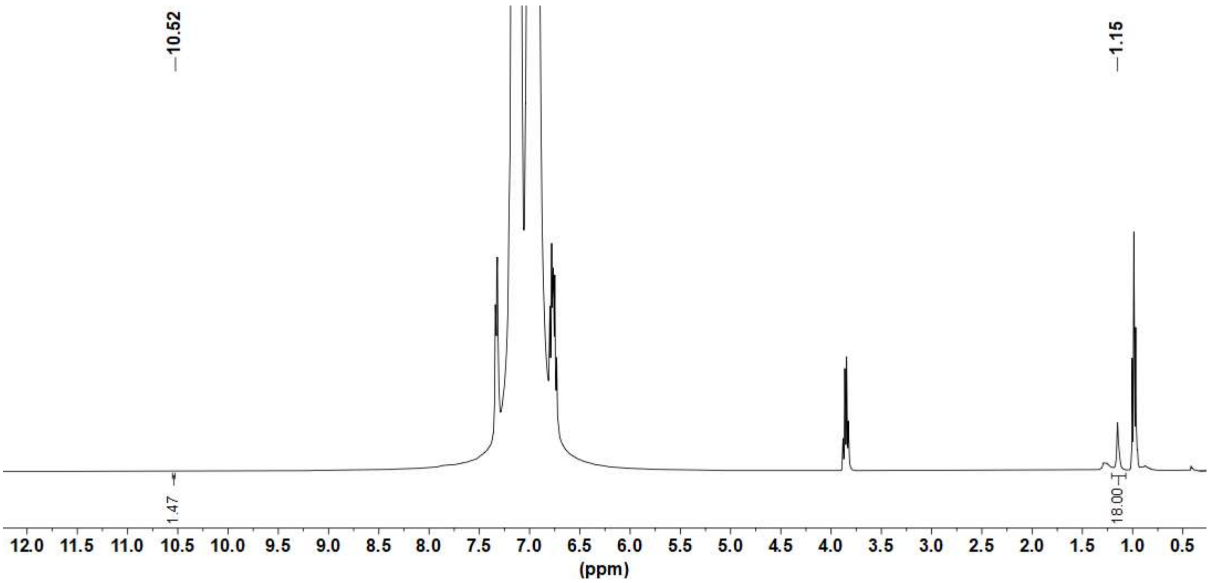

Figure S50: ^{1}H NMR spectrum of protonated *2,6-di-tert-butyl-pyridine* in PhCl. *Note: impurity resonances at 3.99 and 1.06 ppm are due to diethylether.*

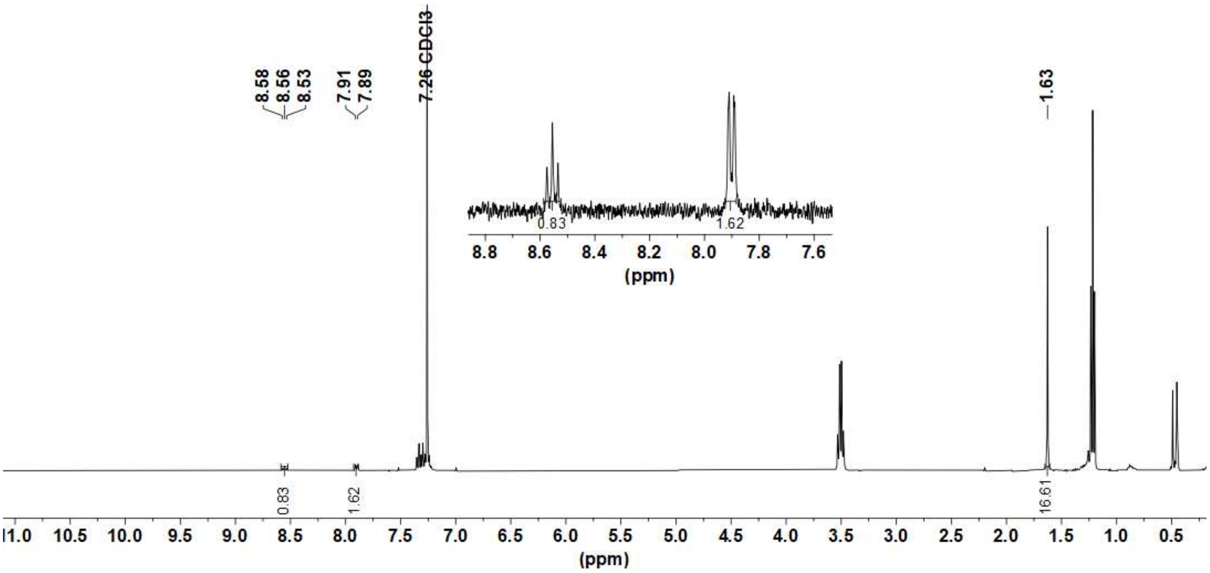

Figure S51: ^{1}H NMR spectrum of protonated *2,6-di-tert-butyl-pyridine* in $CDCl_3$. *Note: impurity resonances at 3.49 and 1.15 ppm are due to diethylether.*

NMR Spectra of (**10**):

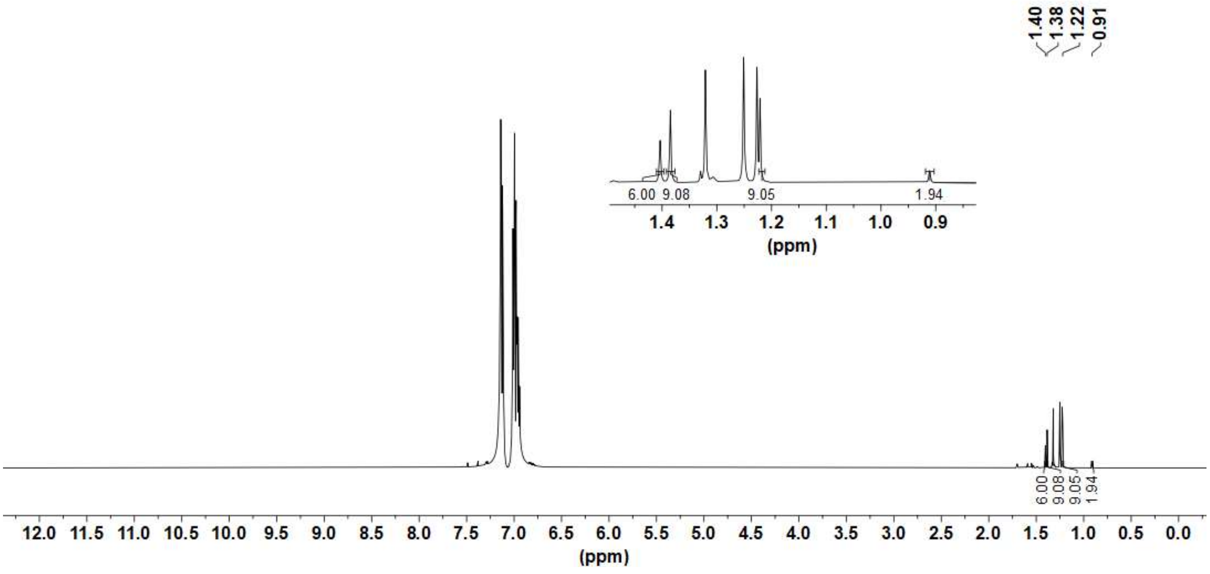

Figure S52: ^{1}H NMR spectrum of (**10**) in PhCl. *Note: other resonances are due to protonated 2,4,6-tri-^{t}Bu-pyrimidine (see* **Figure S59***).*

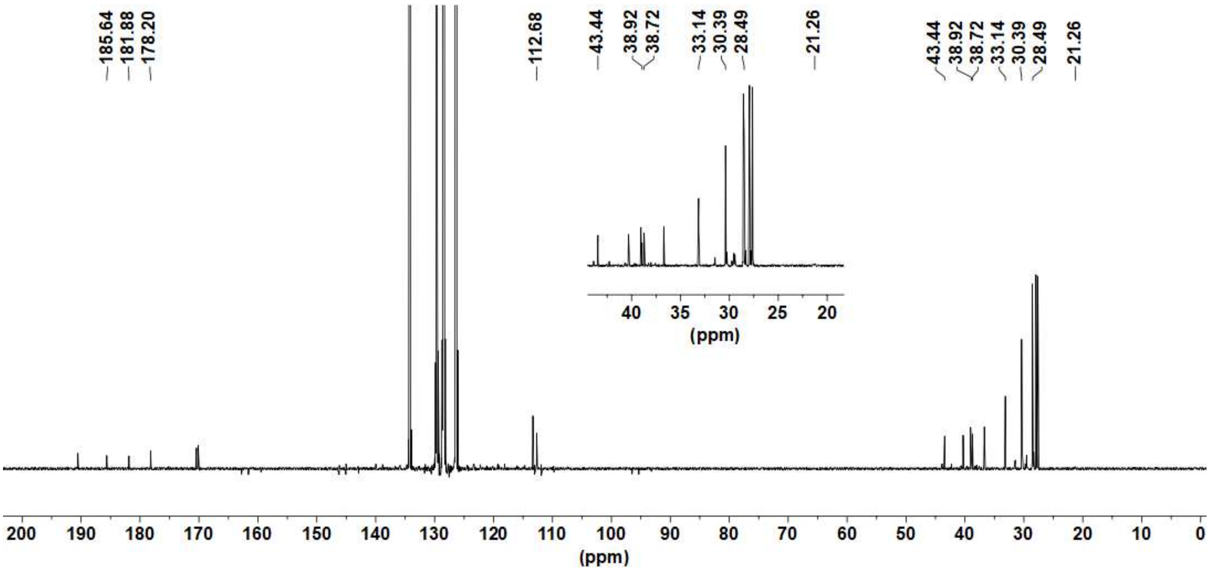

Figure S53: $^{13}C\{^{1}H\}$ NMR spectrum of (**10**) in PhCl. *Note: other resonances are due to protonated 2,4,6-tri-^{t}Bu-pyrimidine (see* **Figure S60***).*

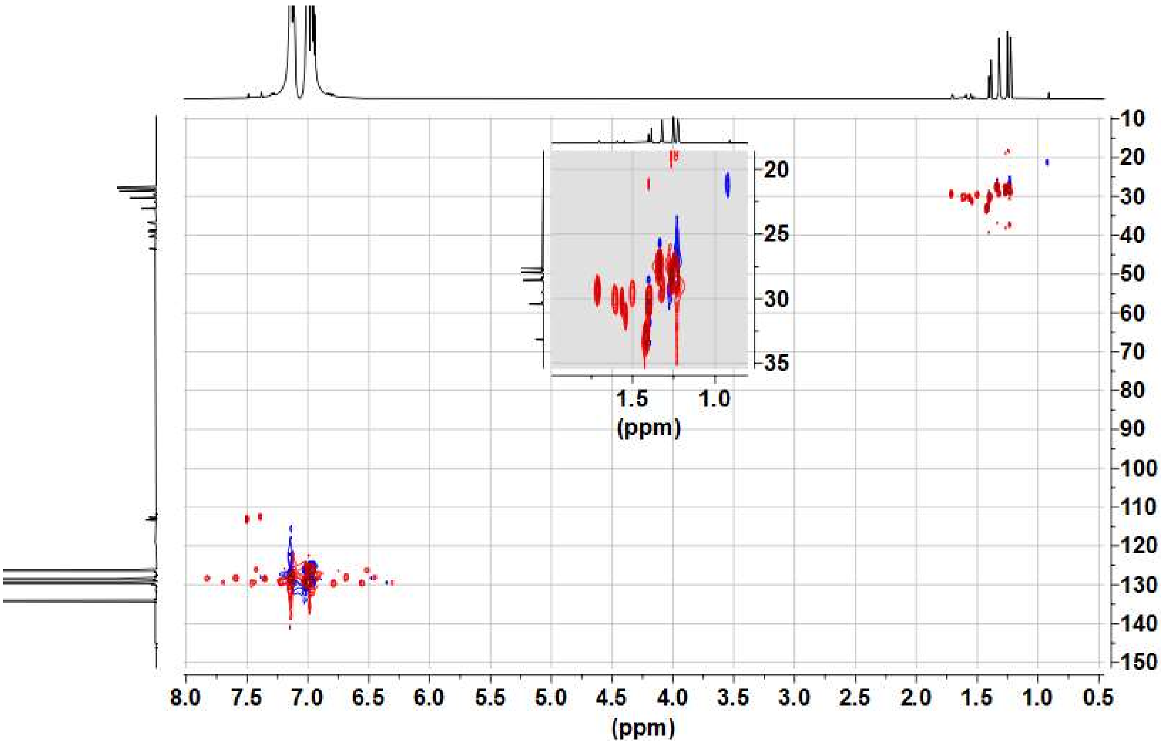

Figure S54: $^{1}H-^{13}C\{^{1}H\}$ HSQC NMR spectrum of (**10**) in PhCl. *Note: other resonances are due to protonated 2,4,6-tri-^{t}Bu-pyrimidine (see* **Figure S59** *and* **Figure S60***).*

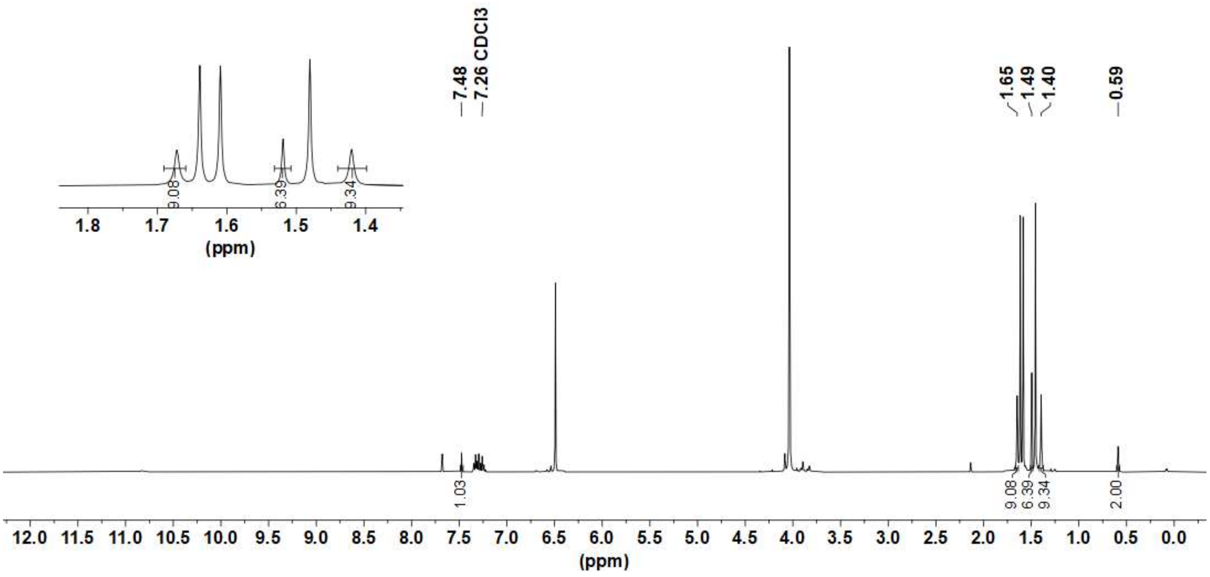

Figure S55: ^{1}H NMR spectrum of (**10**) in $CDCl_3$. *Note: other resonances are due to protonated 2,4,6-tri-^{t}Bu-pyrimidine (see* **Figure S61***) and internal standard (trimethoxybenzene).*

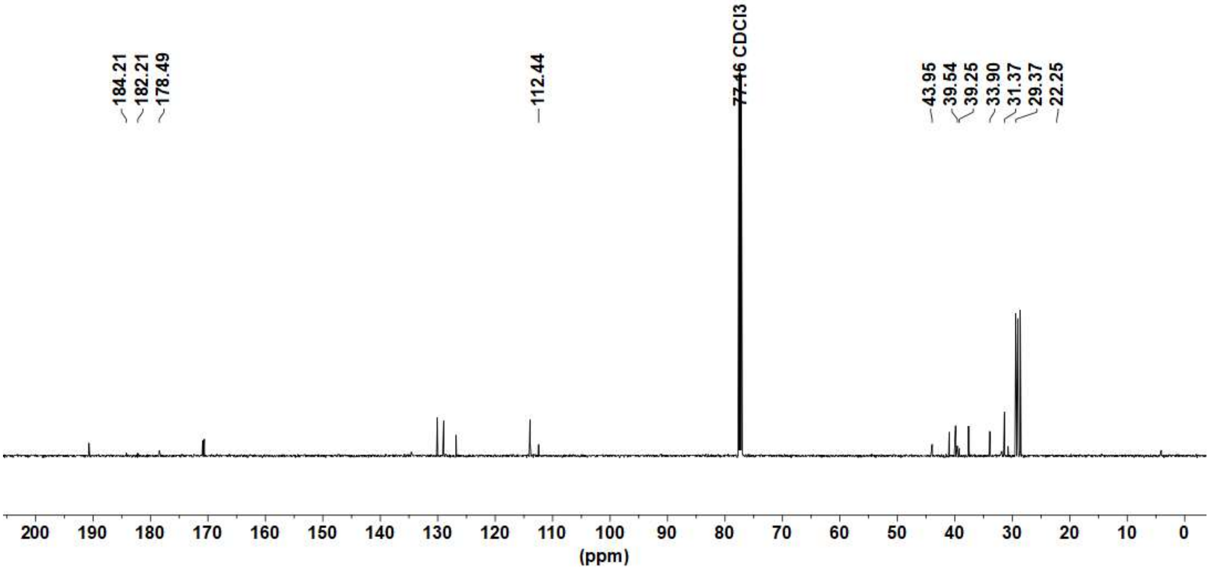

Figure S56: $^{13}C\{^{1}H\}$ NMR spectrum of (**10**) in $CDCl_3$. *Note: other resonances are due to protonated 2,4,6-tri-^{t}Bu-pyrimidine (see* **Figure S62***). Al−CH_2 signal was confirmed by ^{1}H-$^{13}C\{^{1}H\}$ HSQC. Impurity resonances at 134.35 ppm, 129.87 ppm, 128.76 ppm and 126.59 ppm correspond to chlorobenzene.*

NMR Spectra of iodinated 2,4,6-tri-^{t}Bu-pyrimidine:

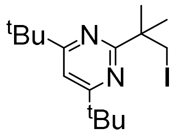

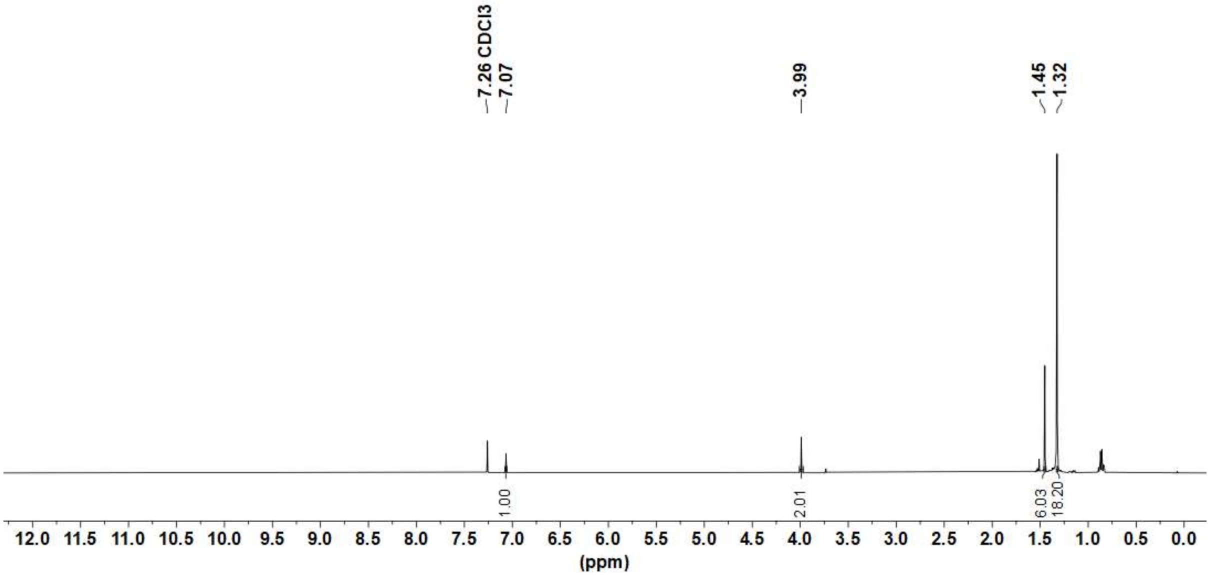

Figure S57: ^{1}H NMR spectrum of iodinated 2,4,6-tri-^{t}Bu-pyrimidine in $CDCl_3$.

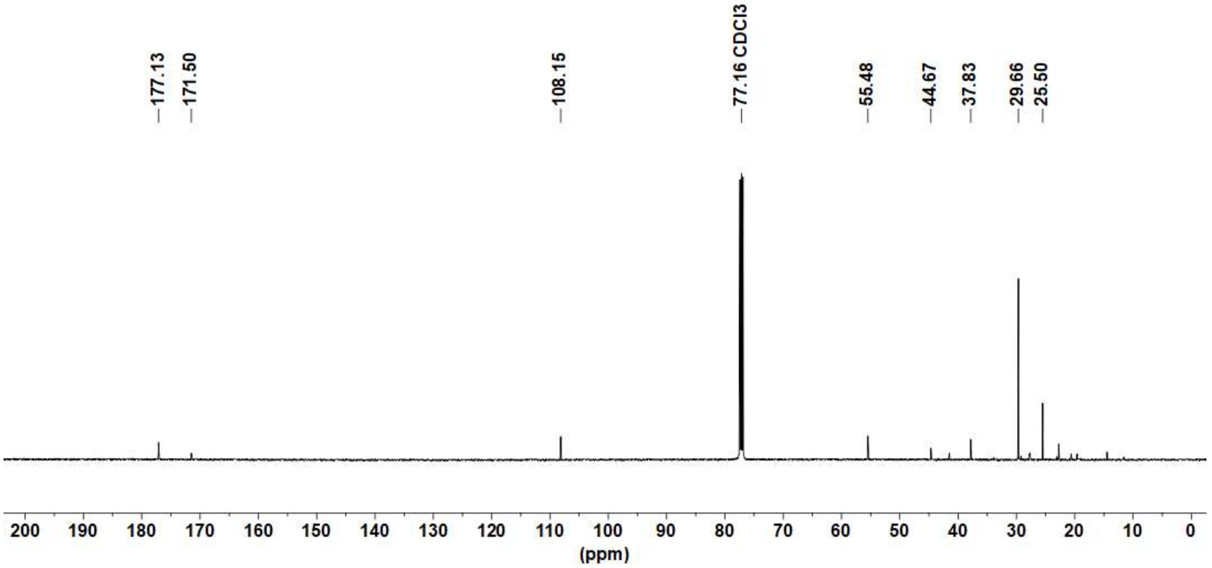

Figure S58: $^{13}C\{^{1}H\}$ NMR spectrum of iodinated 2,4,6-tri-^{t}Bu-pyrimidine in $CDCl_3$.

NMR Spectra of protonated 2,4,6-tributypyrimidine[$AlCl_4$] salt:

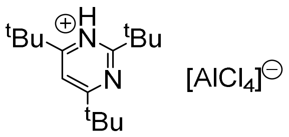

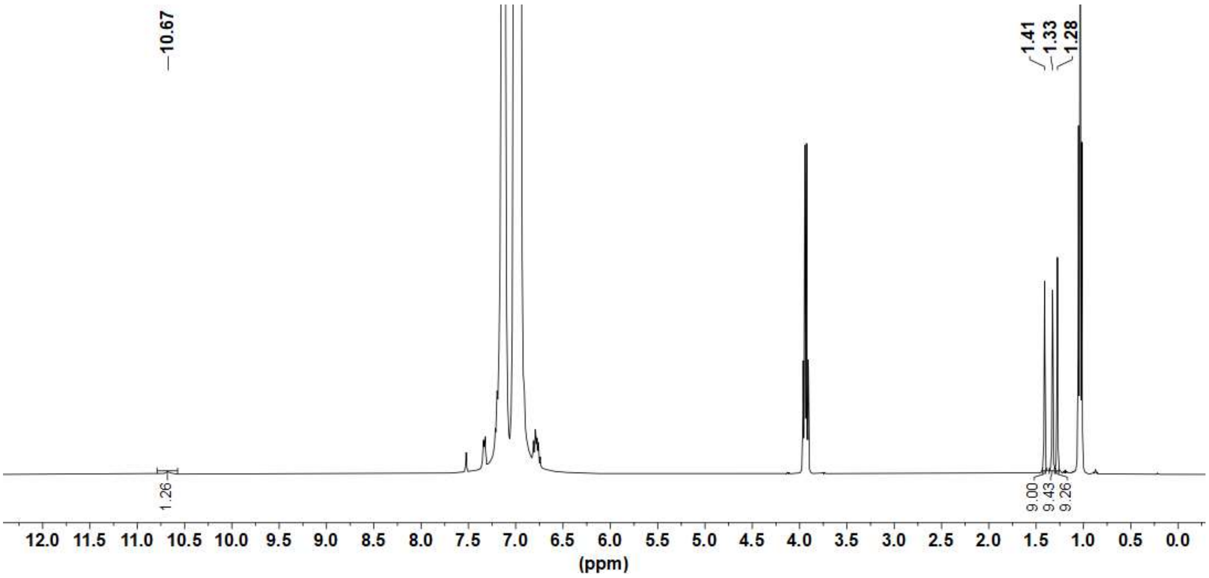

Figure S59: ^{1}H NMR spectrum of protonated 2,4,6-tri-^{t}Bu-pyrimidine in PhCl. *Note: impurity resonances at 3.99 and 1.06 ppm are due to diethylether.*

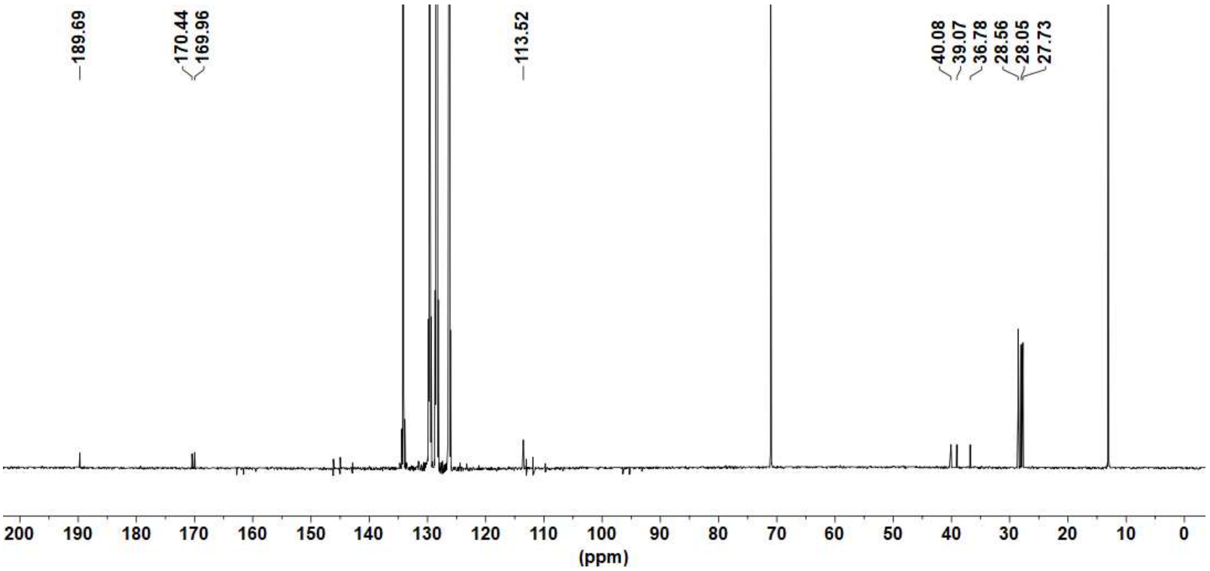

Figure S60: ^{13}C{^{1}H} NMR spectrum of protonated 2,4,6-tri-^{t}Bu-pyrimidine in PhCl. *Note: impurity resonances at 71.28 and 13.16 ppm are due to diethylether.*

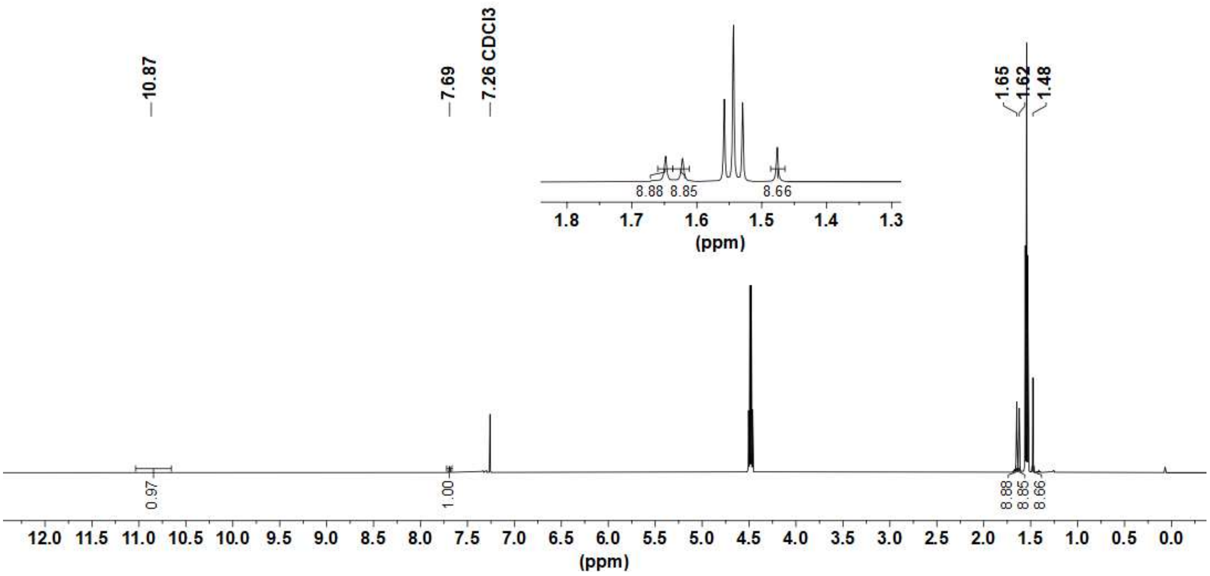

Figure S61: ^{1}H NMR spectrum of protonated 2,4,6-tri-^{t}Bu-pyrimidine in $CDCl_3$. *Note: impurity resonances at 4.45 and 1.55 ppm are due to diethylether.*

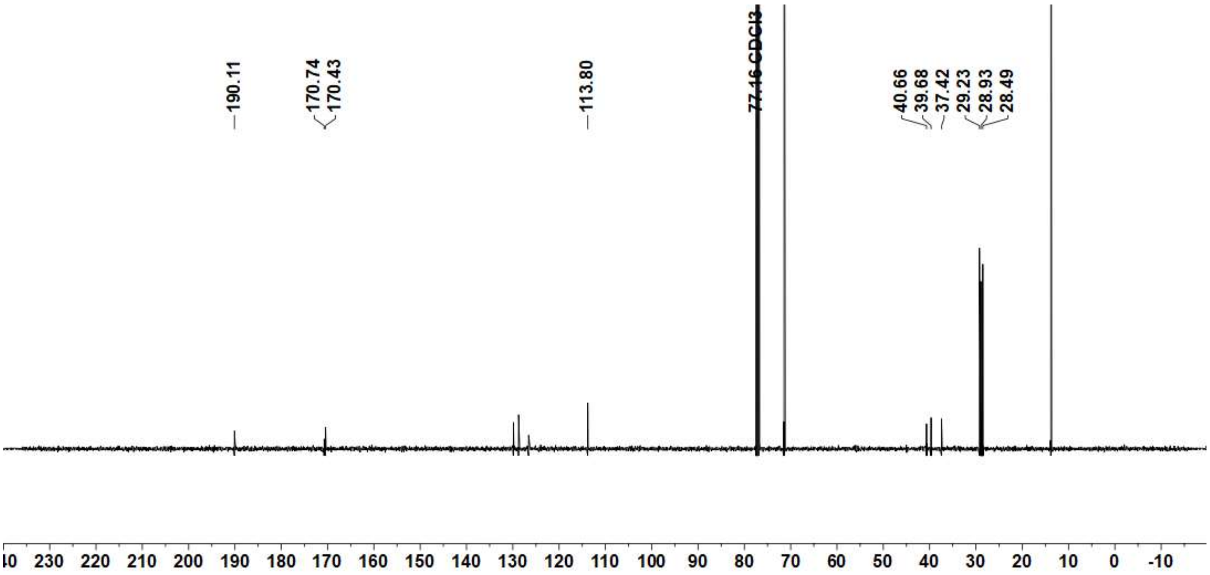

Figure S62: $^{13}C\{^{1}H\}$ NMR spectrum of protonated 2,4,6-tri-^{t}Bu-pyrimidine in $CDCl_3$. *Note: impurities at 71.28 and 13.16 ppm are due to diethylether. Impurity resonances at 134.35 ppm, 129.87 ppm, 128.76 ppm and 126.59 ppm correspond to chlorobenzene.*

NMR Spectra of (**11**):

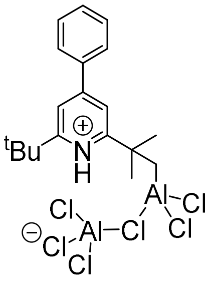

11

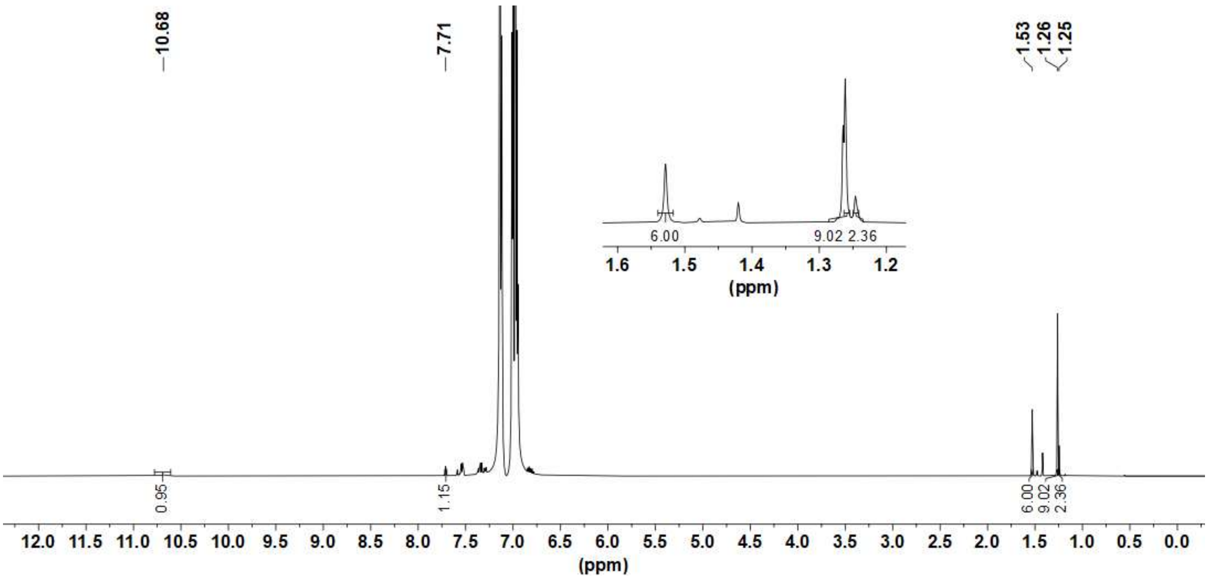

Figure S63: ^{1}H NMR spectrum of (**11**) in PhCl. *Note: other resonances are due to protonated 2,6-di-tert-butyl-4-phenyl-pyridine and starting material (see* **Figure S68***).*

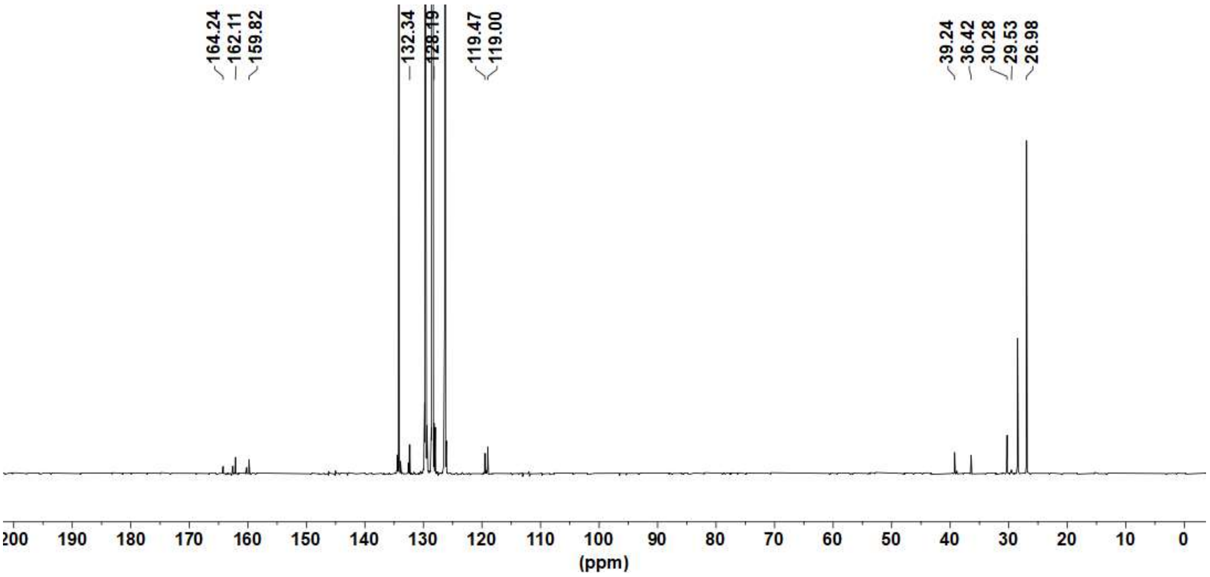

Figure S64: $^{13}C\{^1H\}$ NMR spectrum of (**11**) in PhCl. *Note: other resonances are due to protonated 2,6-di-tert-butyl-4-phenyl-pyridine and starting material. Some Aryl−H resonances are covered by chlorobenzene.*

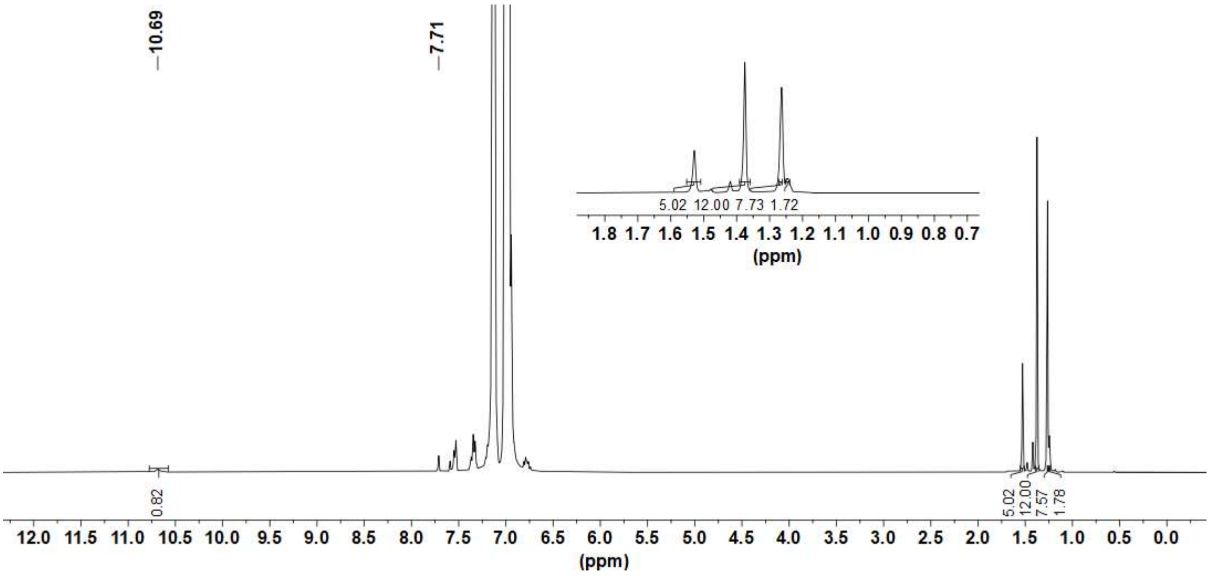

Figure S65: ^{1}H NMR spectrum of (**11**) in PhCl with internal standard (cyclohexane). *Note: other resonances are due to protonated 2,6-di-tert-butyl-4-phenyl-pyridine and starting material (see* **Figure S68***).*

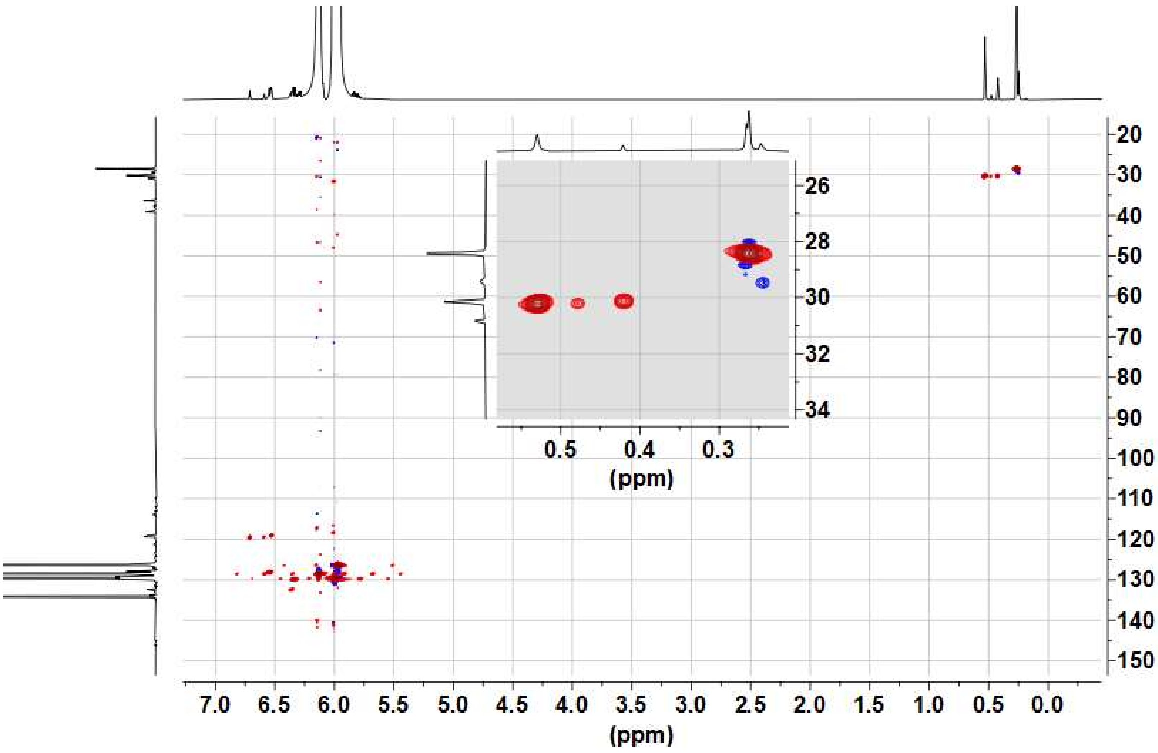

Figure S66: ^{1}H−^{13}C{^{1}H} HSQC NMR spectrum of (**11**) in PhCl. *Note: other resonances are due to protonated 2,6-di-tert-butyl-4-phenyl-pyridine.*

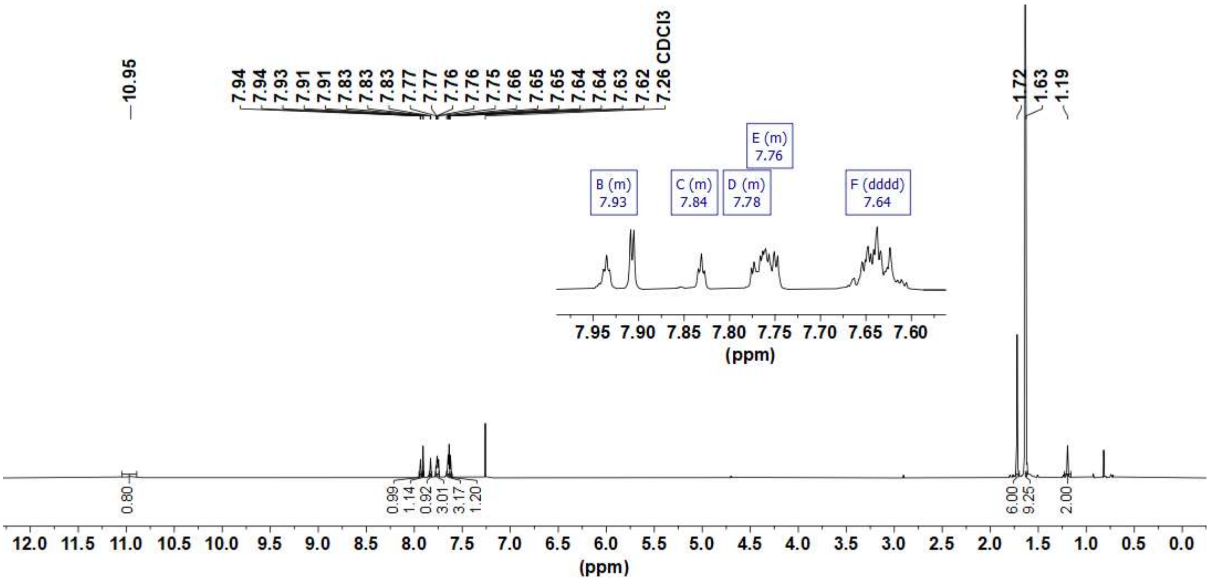

Figure S67: ^{1}H NMR spectrum of (**11**) in $CDCl_3$. *Note: other resonances are due to protonated 2,6-di-tert-butyl-4-phenyl-pyridine.*

NMR Spectra of 2,6-tertbutyl-4-phenylpyridine:

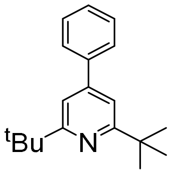

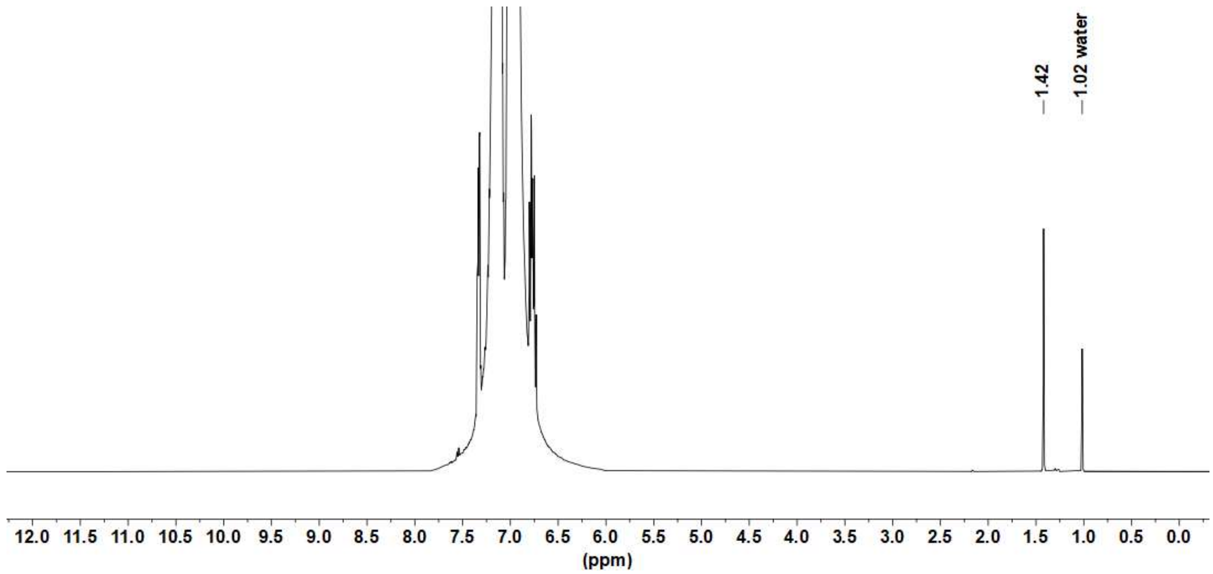

Figure S68: ^{1}H NMR spectrum of (**11**) in PhCl. *Note: Impurity at 1.02 ppm is due to water.*

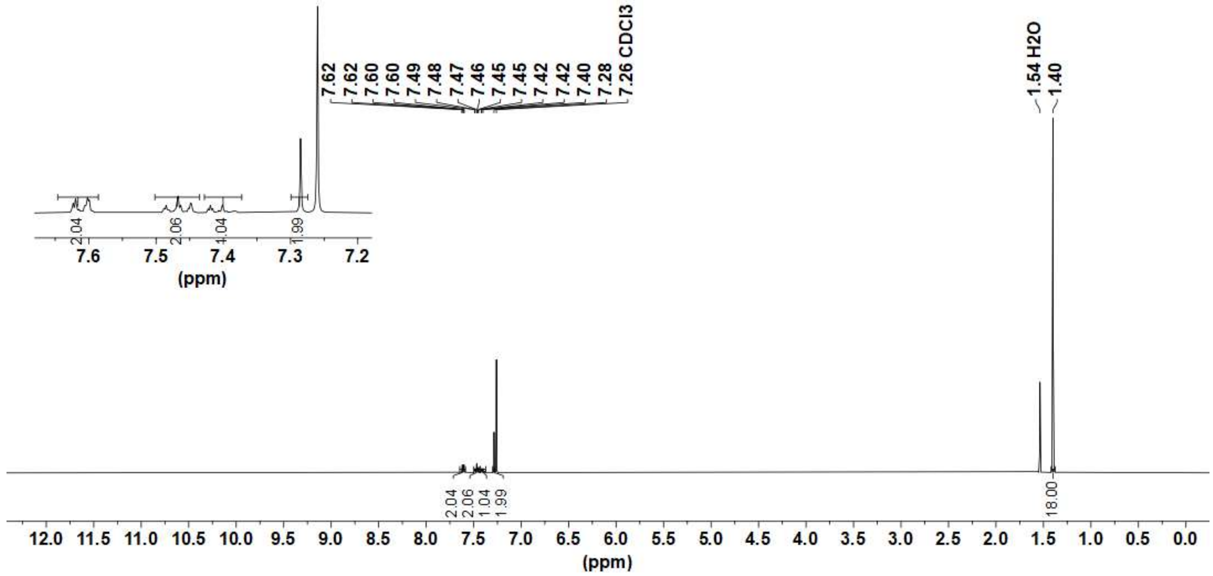

Figure S69: ^{1}H NMR spectrum of starting material 2,6-di-tert-butyl-4-phenyl-pyridine in $CDCl_3$.

NMR Spectra of iodonated 2,6-di-tertbutyl-4-phenylpyridine:

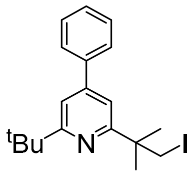

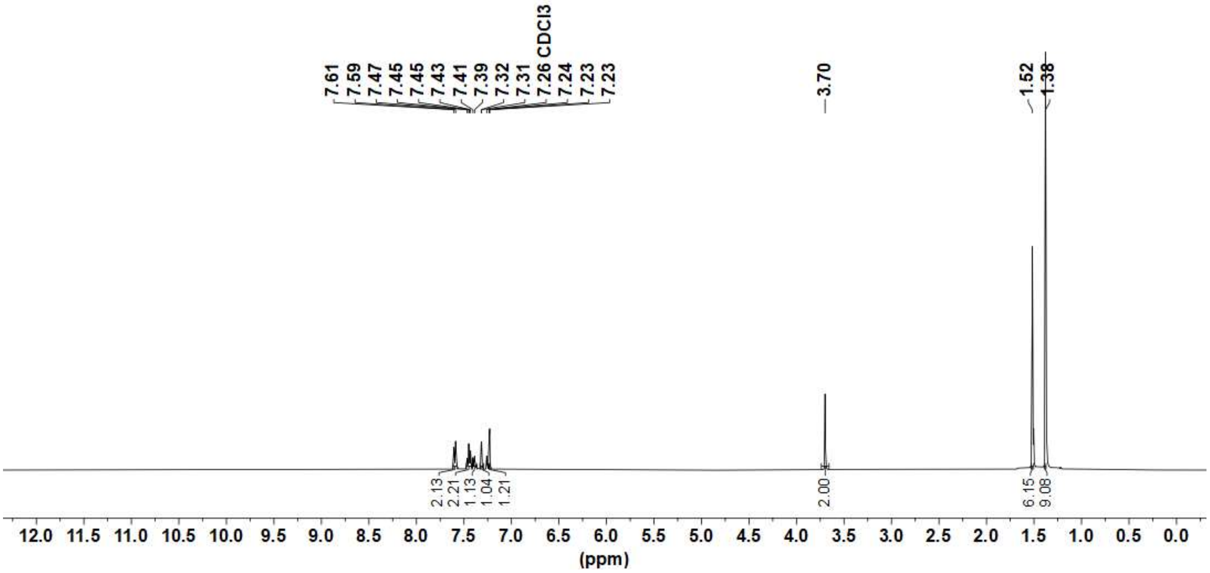

Figure S70: ^{1}H NMR spectrum of iodinated *2,6-di-tert-butyl-4-phenyl-pyridine* in $CDCl_3$.

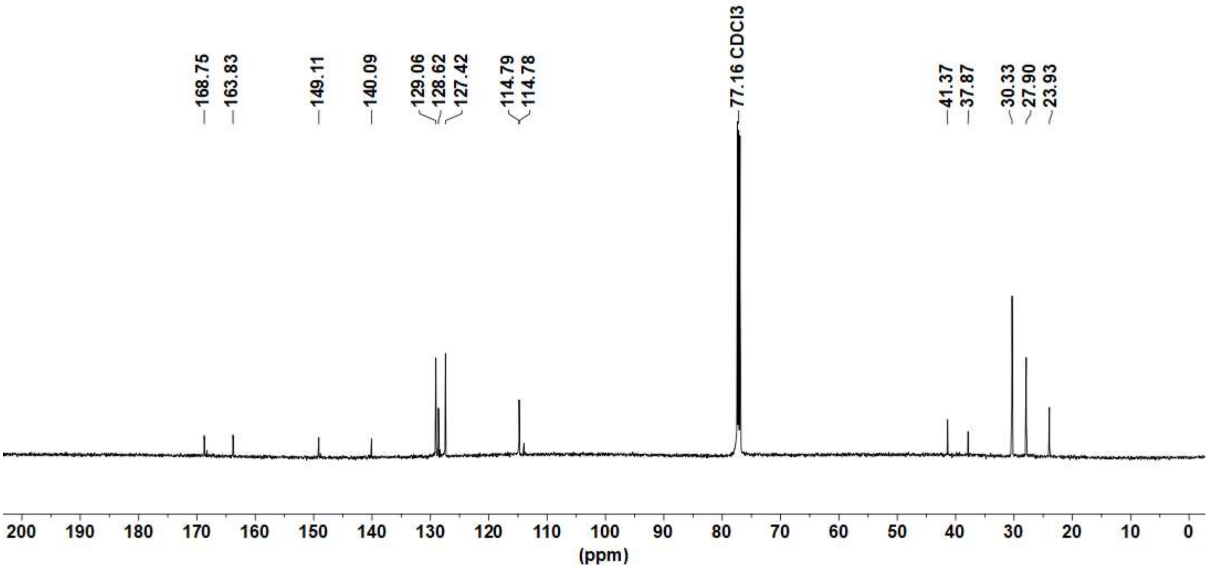

Figure S71: $^{13}C\{^{1}H\}$ NMR spectrum of iodinated *2,6-di-tert-butyl-4-phenyl-pyridine* in $CDCl_3$.

NMR Spectra of protonated 2,6-di-tertbutyl-4-phenylpyridine[$AlCl_4$] salt:

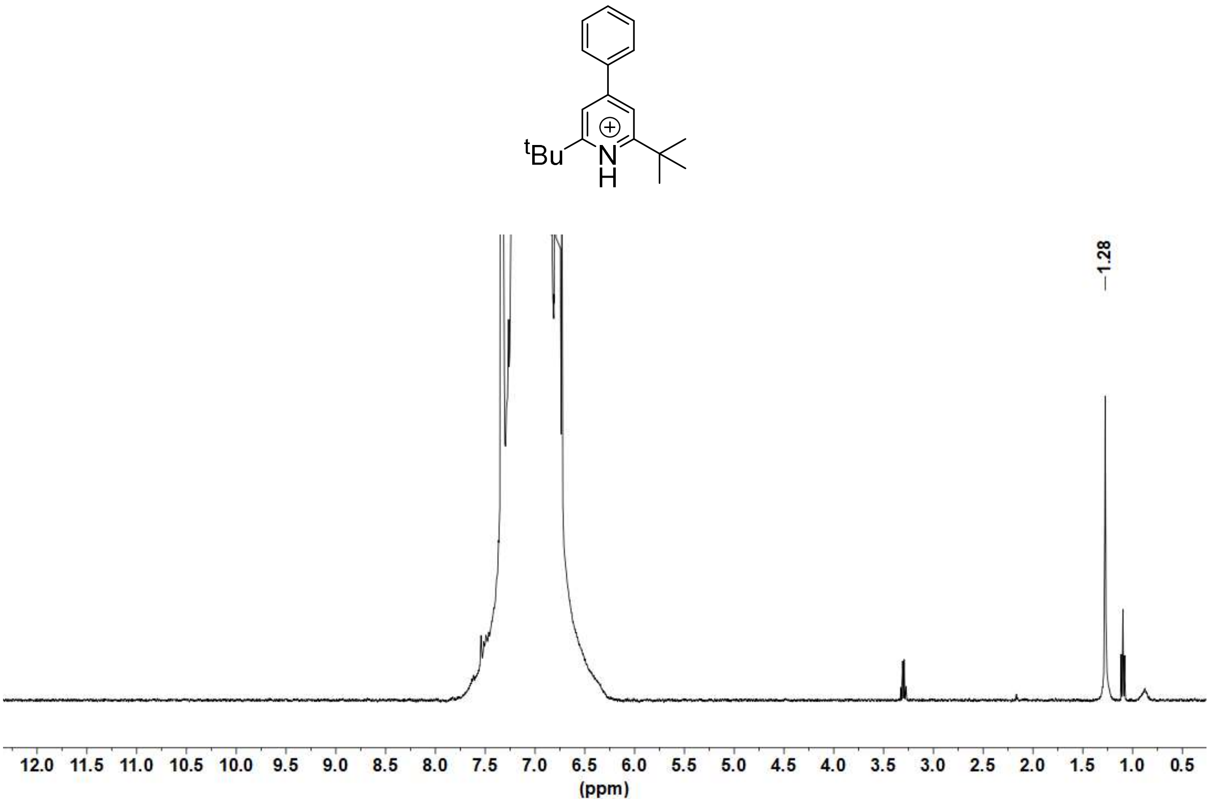

Figure S72: ^{1}H NMR spectrum of protonated 2,6-di-tert-butyl-4-phenyl-pyridine in PhCl. *Note: impurity resonances at 3.49 and 0.7 ppm are due to diethylether.*

NMR Spectra of (**12**):

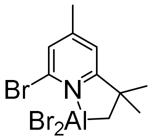

12

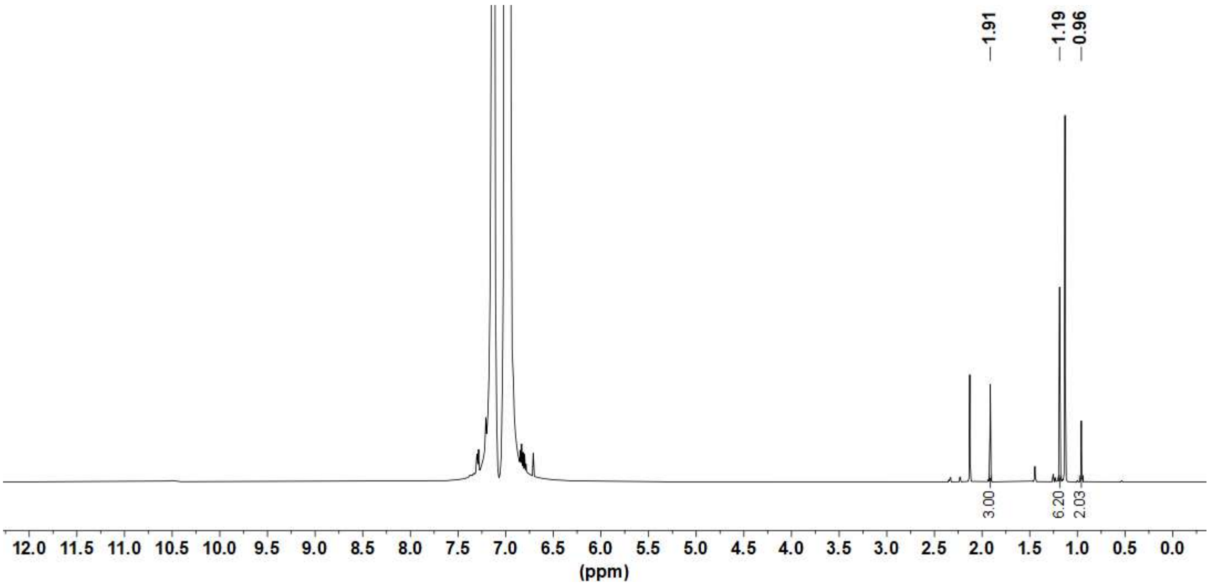

Figure S 73: ^{1}H NMR spectrum of (**12**) in PhCl. *Note: other resonances are the protonated 2-Br-4-methyl-6-^{t}Bu-pyridine by-product (see* **Figure S79***).*

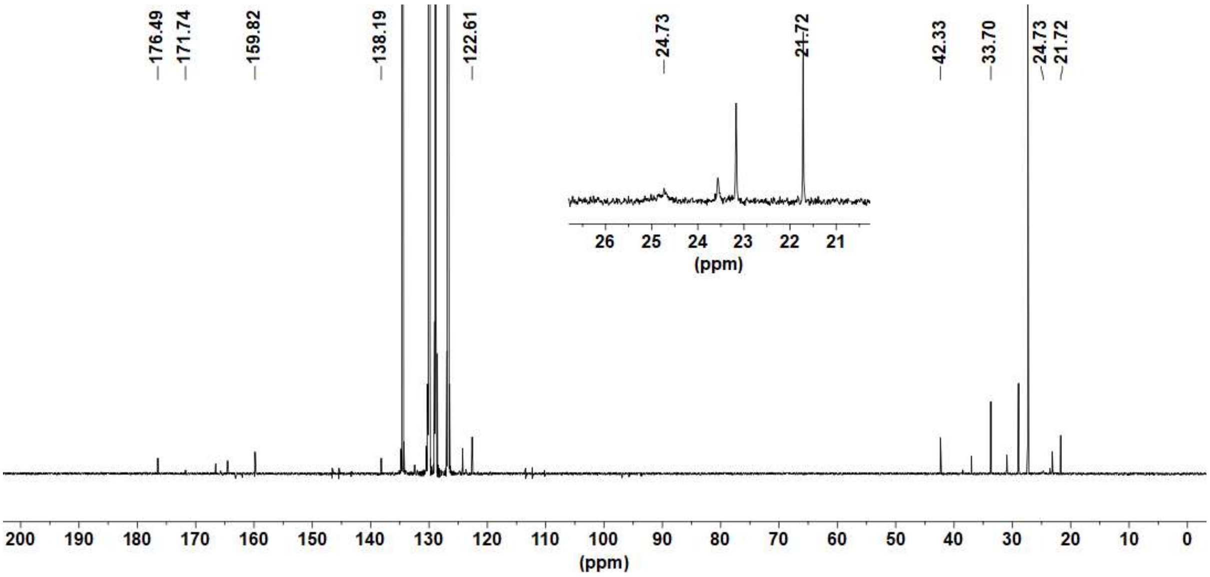

Figure S74: $^{13}C\{^{1}H\}$ NMR spectrum of (**12**) in PhCl. *Note: other resonances are protonated 2-Br-4-methyl-6-^{t}Bu-pyridine by-product (see* **Figure S80***).*

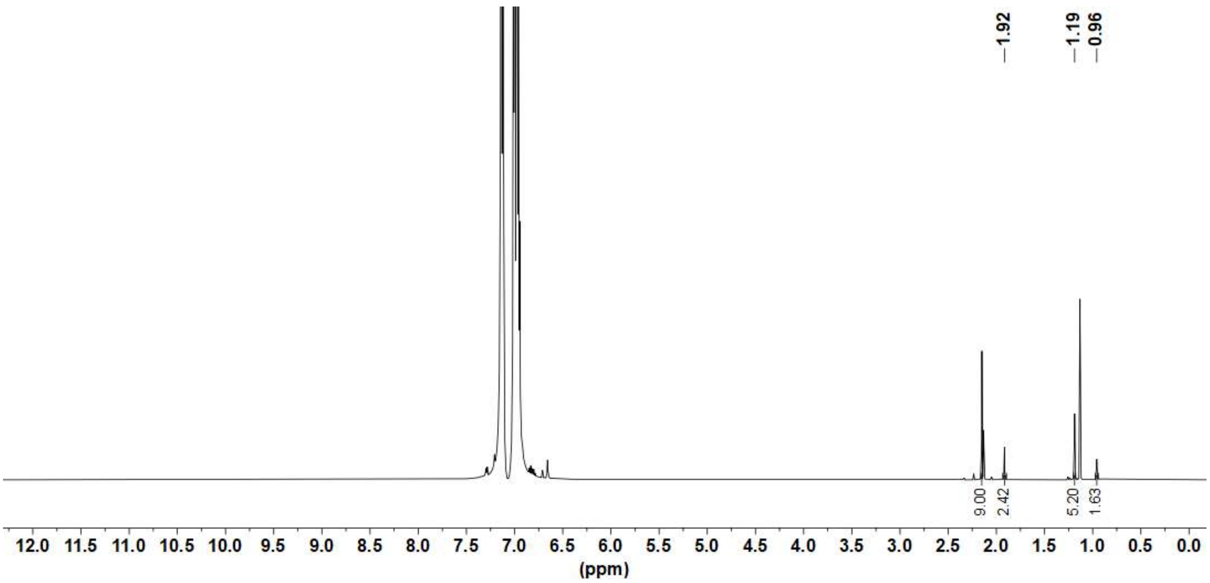

Figure S75: ^{1}H NMR spectrum of (**12**) in PhCl with internal standard (mesitylene, 0.05 mmol). *Note: other resonances are protonated 2-Br-4-methyl-6-^{t}Bu-pyridine by-product (see* **Figure S79***).*

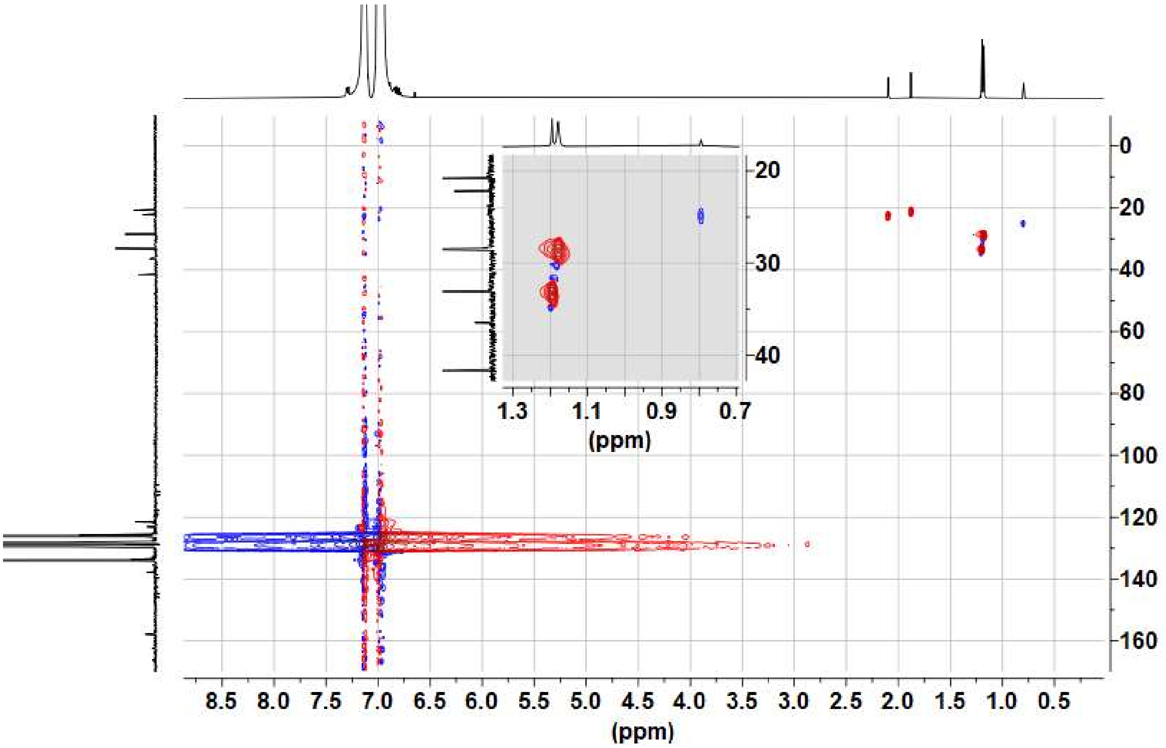

Figure S76: $^{1}H-^{13}C\{^{1}H\}$ HSQC NMR spectrum of (**12**) in PhCl. *Note: other resonances are protonated 2-Br-4-methyl-6-^{t}Bu-pyridine by-product (see* **Figure S79** *and* **Figure S80***).*

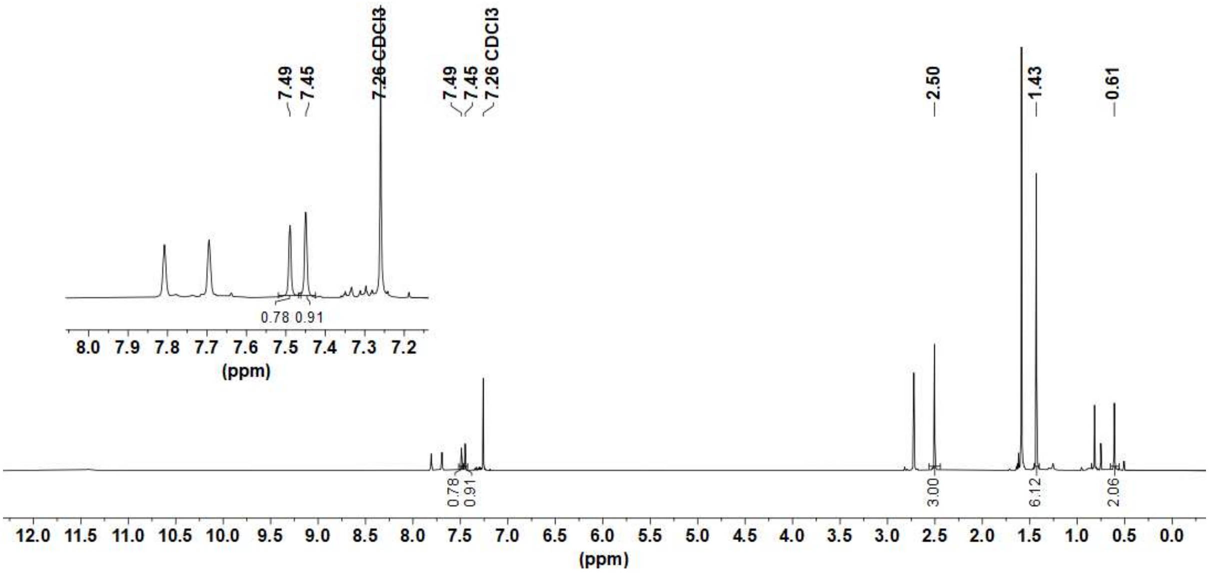

Figure S77: ^{1}H NMR spectrum of (**12**) in $CDCl_3$. *Note: other resonances are due to protonated 2-Br-4-methyl-6-^{t}Bu-pyridine by-product (see* **Figure S81***).*

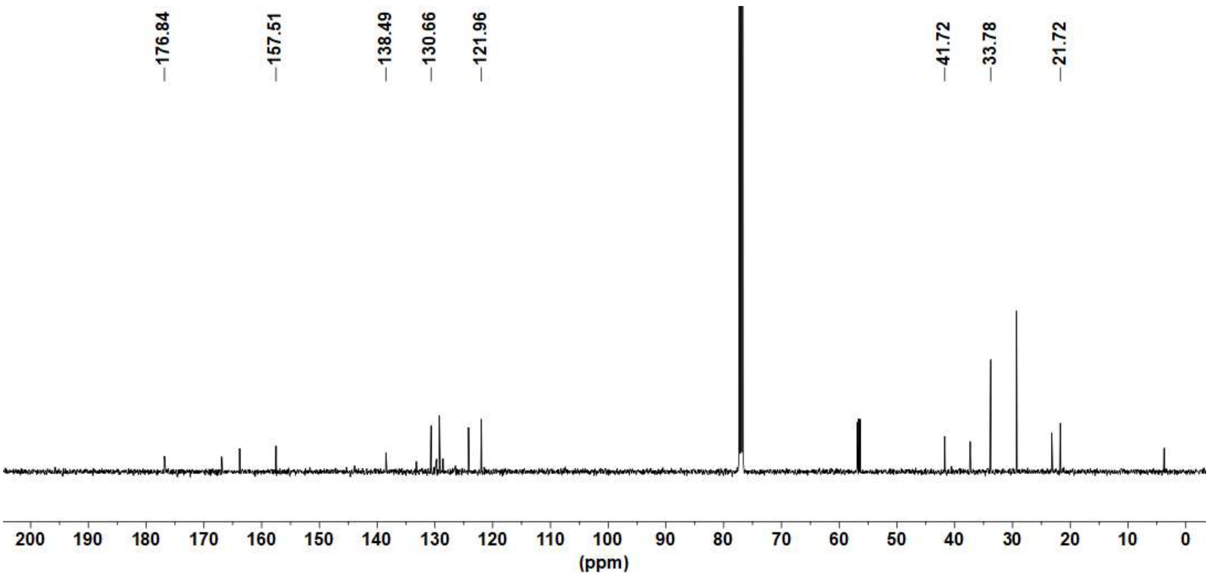

Figure S78: $^{13}C\{^1H\}$ NMR spectrum of (**12**) in $CDCl_3$. *Note: other resonances are protonated 2-Br-4-methyl-6-^{t}Bu-pyridine by-product (see* **Figure S82***). Impurities at 134.35, 129.86, 128.73 and 126.59 correspond to chlorobenzene and at 55 to CD_2Cl_2.*

NMR Spectra of protonated **2Br-py[AlCl$_4$] salt**:

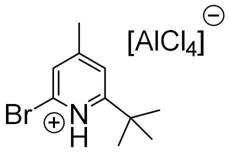

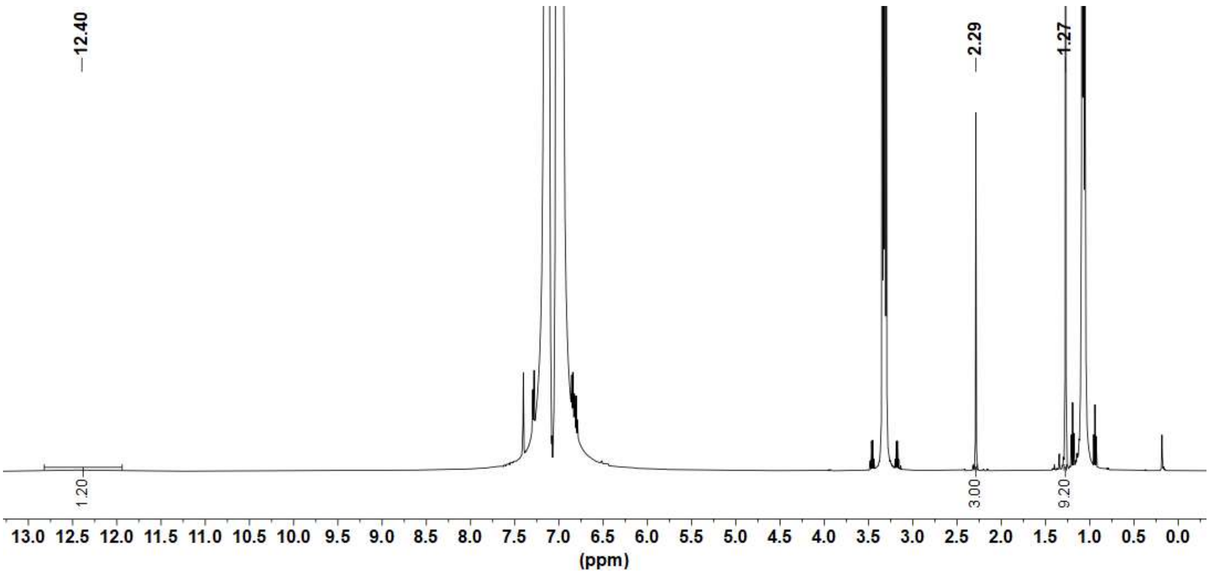

Figure S79: ^{1}H NMR spectrum of protonated **2Br-py** in PhCl. *Note: impurity resonances at 3.49 and 1.12 ppm are due to diethylether.*

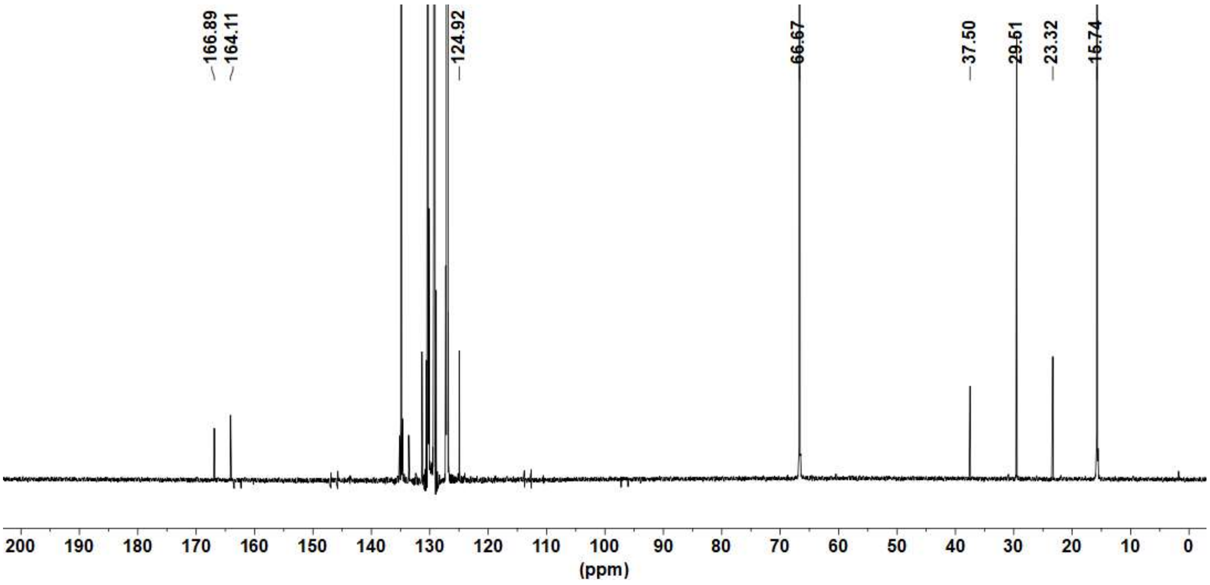

Figure S80: ^{13}C{^{1}H} NMR spectrum of protonated **2Br-py** in PhCl. *Note: impurity resonances at 66.6 and 15.7 ppm are due to diethyl ether.*

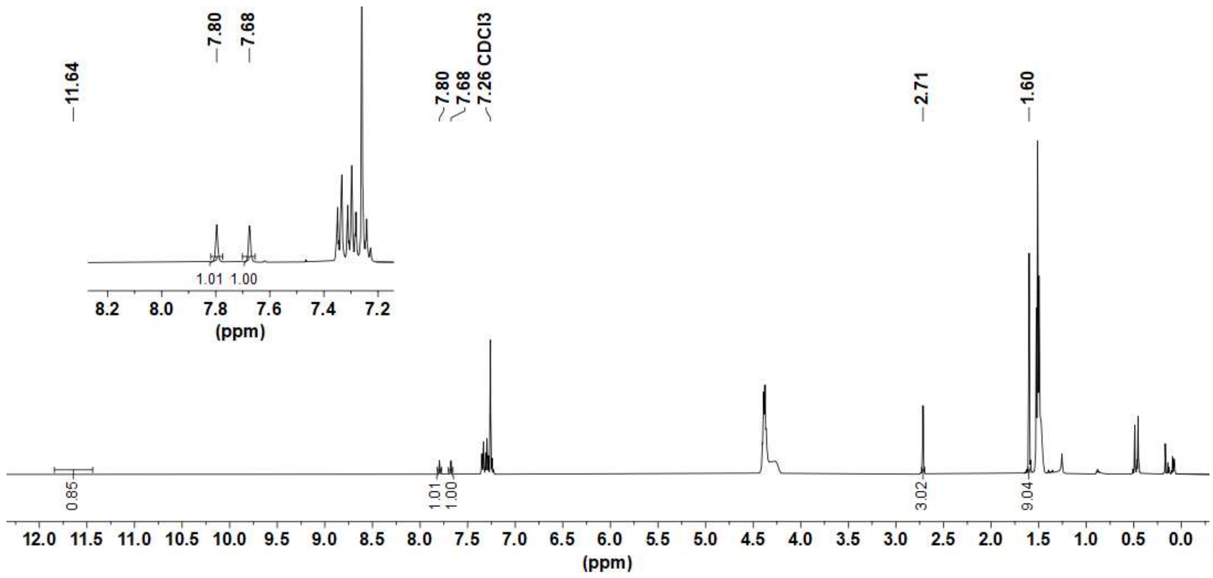

Figure S81: ^{1}H NMR spectrum of protonated **2Br-py** in $CDCl_3$. *Note: impurity resonances at 4.45 and 1.55 ppm are due to diethylether. Impurity resonances at ca. 7.30 ppm is due to chlorobenzene.*

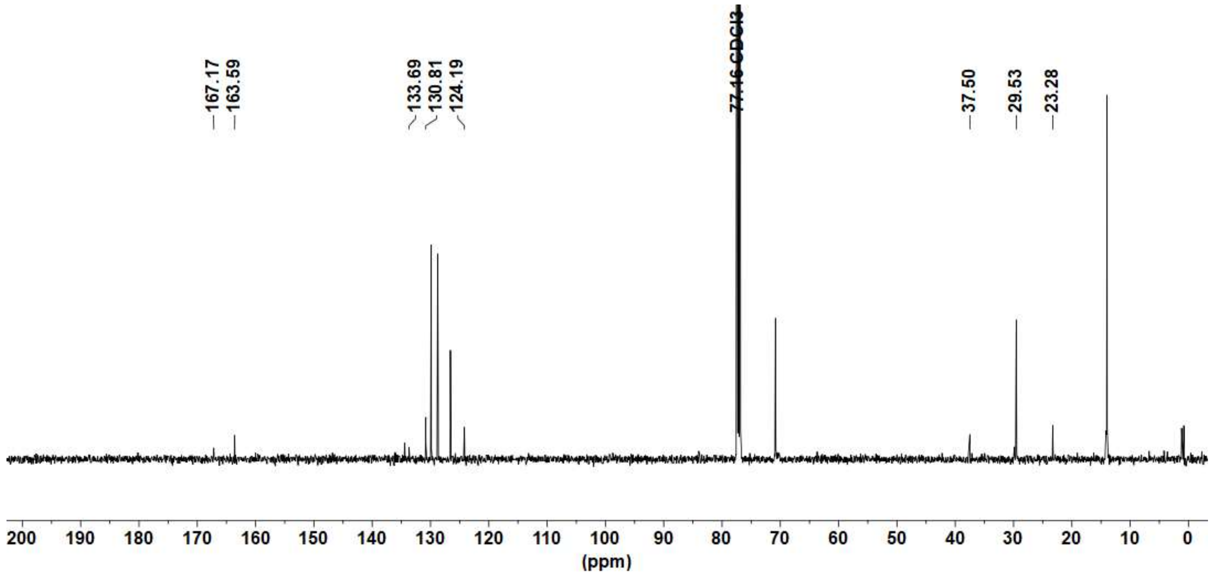

Figure S82: $^{13}C\{^1H\}$ NMR spectrum of protonated **2Br-py** in $CDCl_3$. *Note: impurity resonances at 71.28 and 13.16 ppm are due to diethylether. Resonances at 134.35 ppm, 129.87 ppm, 128.76 ppm and 126.59 ppm correspond to residual chlorobenzene.*

NMR Spectra of (**14**):

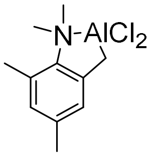

14

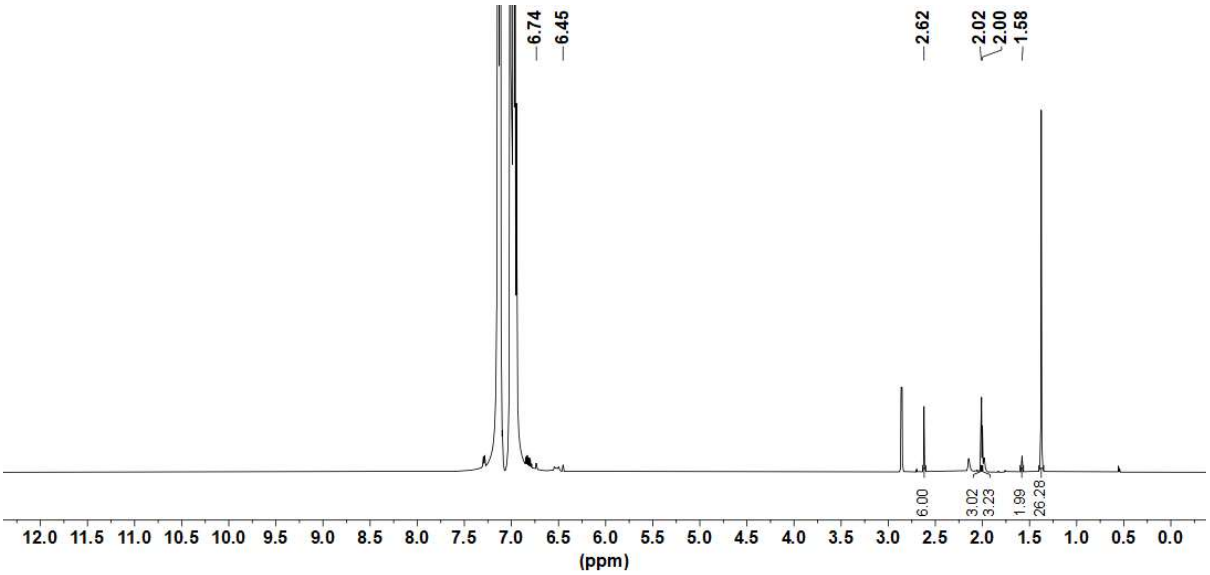

Figure S83: ^{1}H NMR spectrum of (**14**) in PhCl with cyclohexane. *Note: By-product resonances present due to the protonated MesNMe$_2$ (see* **Figure S89***).*

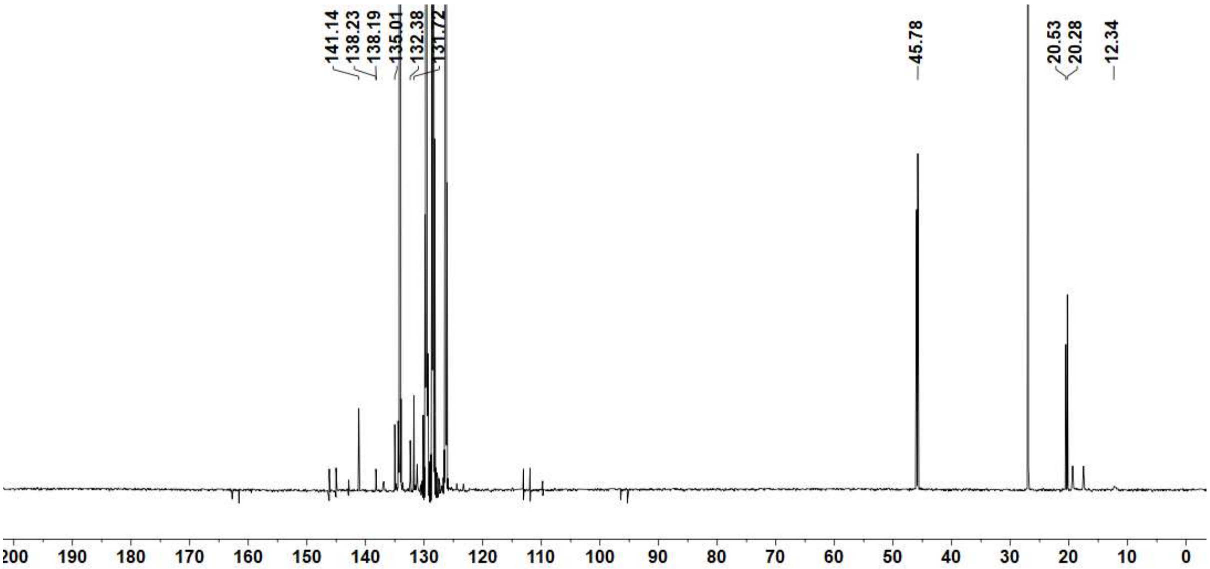

Figure S84: ^{13}C{^{1}H} NMR spectrum of (**14**) in PhCl with internal standard (cyclohexane). *Note: By-product resonances present due to the protonated MesNMe$_2$ (see* **Figure S90***).*

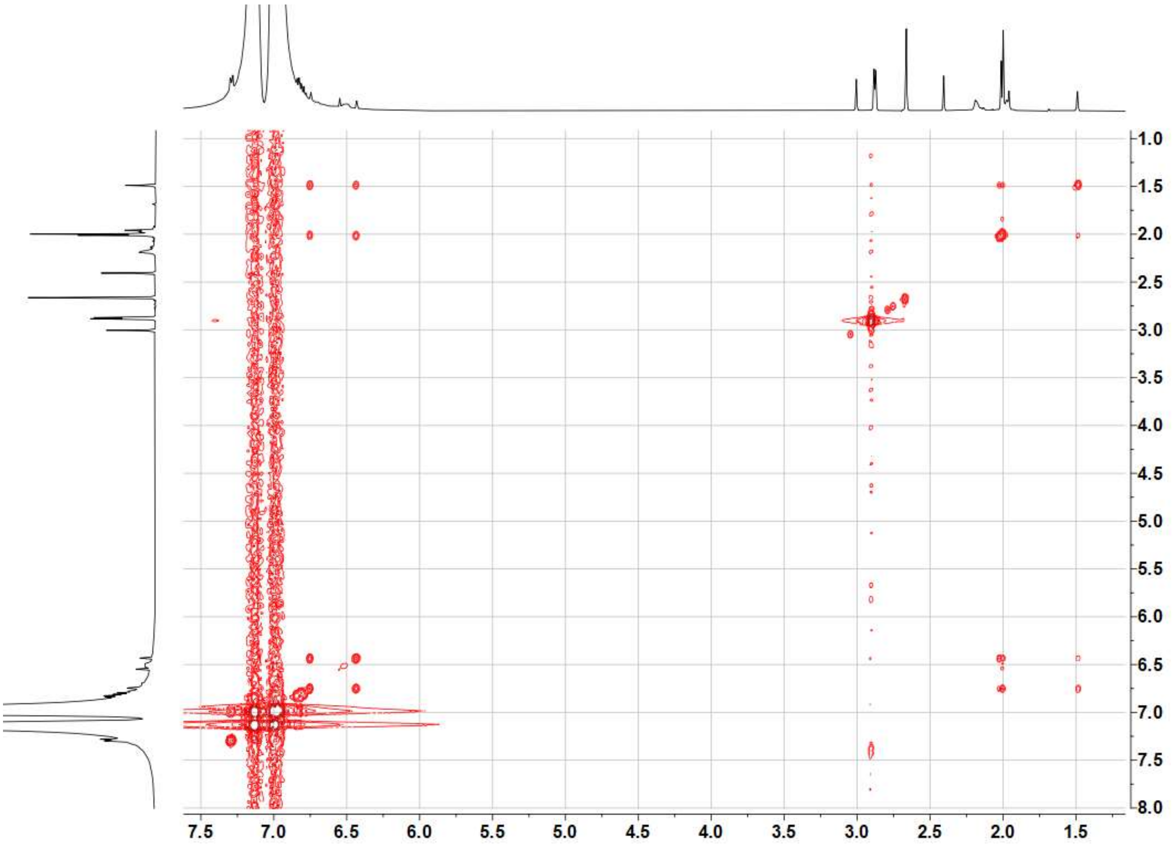

Figure S85: ^{1}H-^{1}H-COSY NMR spectrum of (**14**). *Note: by-product resonances present due to protonated MesNMe₂* (*see* **Figure S89**).

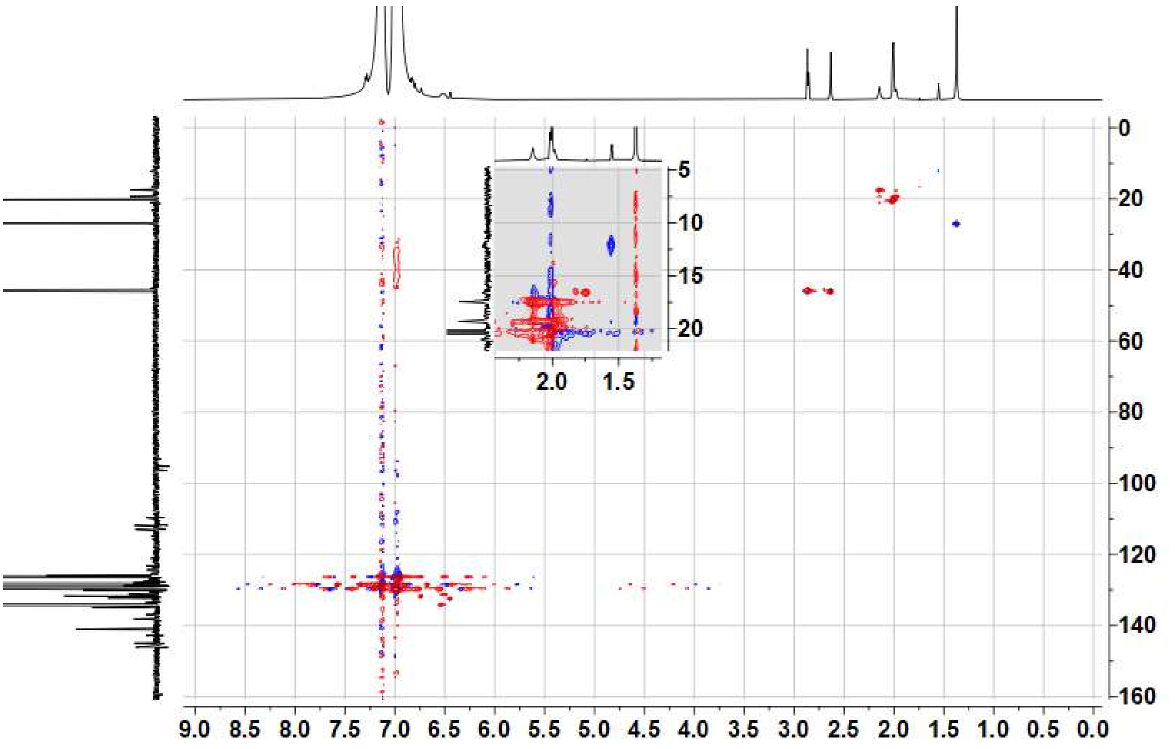

Figure S86: ^{1}H-$^{13}C\{^{1}H\}$-HSQC NMR spectrum of (**14**) in PhCl with internal standard (cyclohexane). *Note: by-product resonances present due to protonated* $MesNMe_2$ (*see* **Figure S89** *and* **Figure S90**).

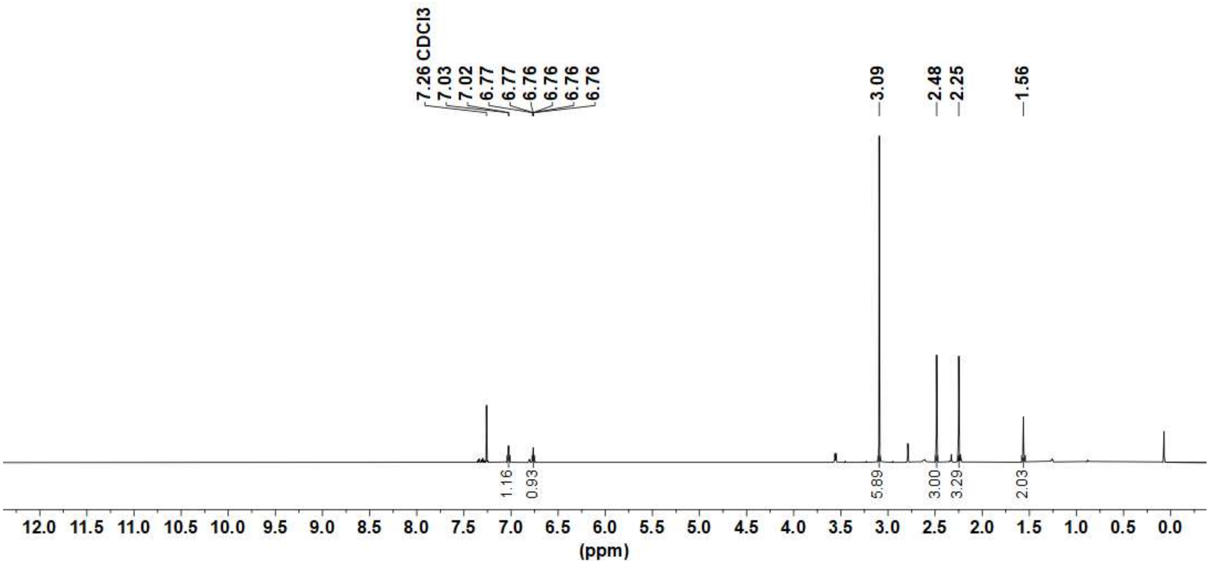

Figure S87: ^{1}H NMR spectrum of (**14**) in $CDCl_3$.

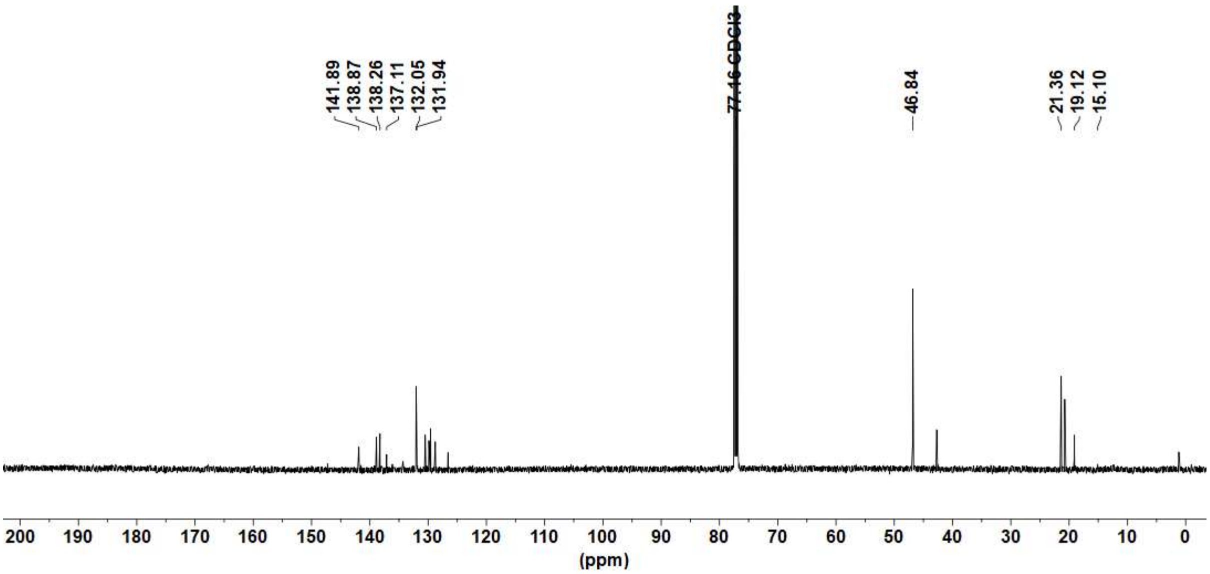

Figure S88: ^{13}C{^{1}H} NMR spectrum of (**14**) in $CDCl_3$. *Note: impurity resonances at 134.35, 129.87 ppm, 128.76 ppm and 126.59 ppm correspond to chlorobenzene.*

NMR Spectra of protonated $MesNMe_2[AlCl_4]$ salt:

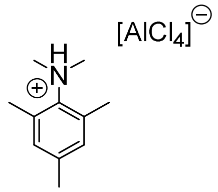

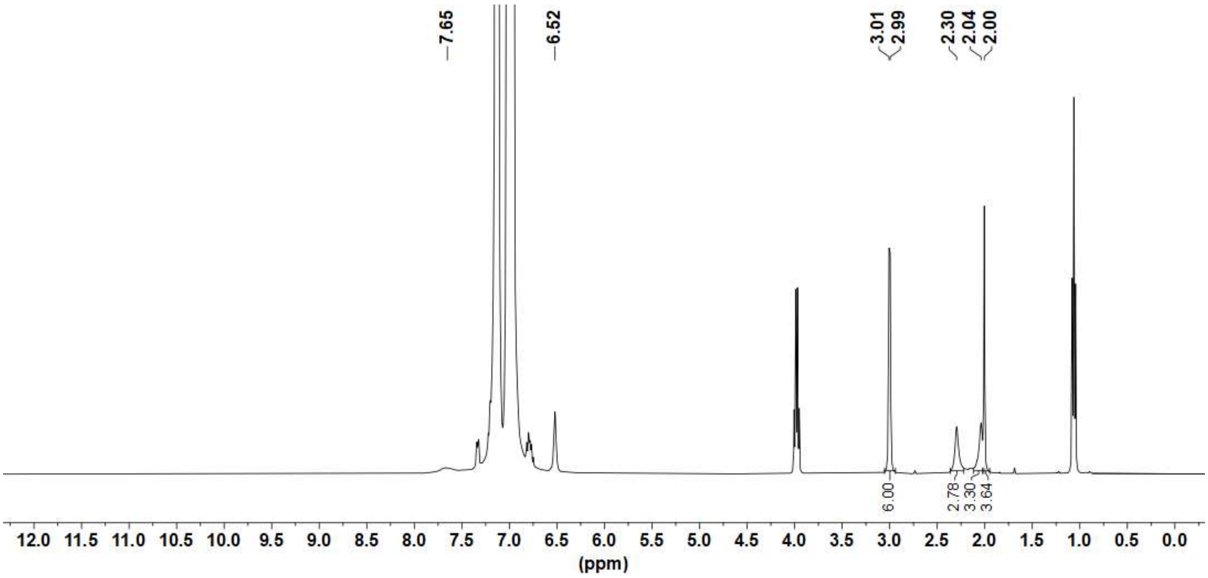

Figure S89: ^{1}H NMR spectrum of (protonated *MesNMe$_2$*) in PhCl. *Note: impurity resonances at 3.99 and 1.06 ppm are due to diethylether.*

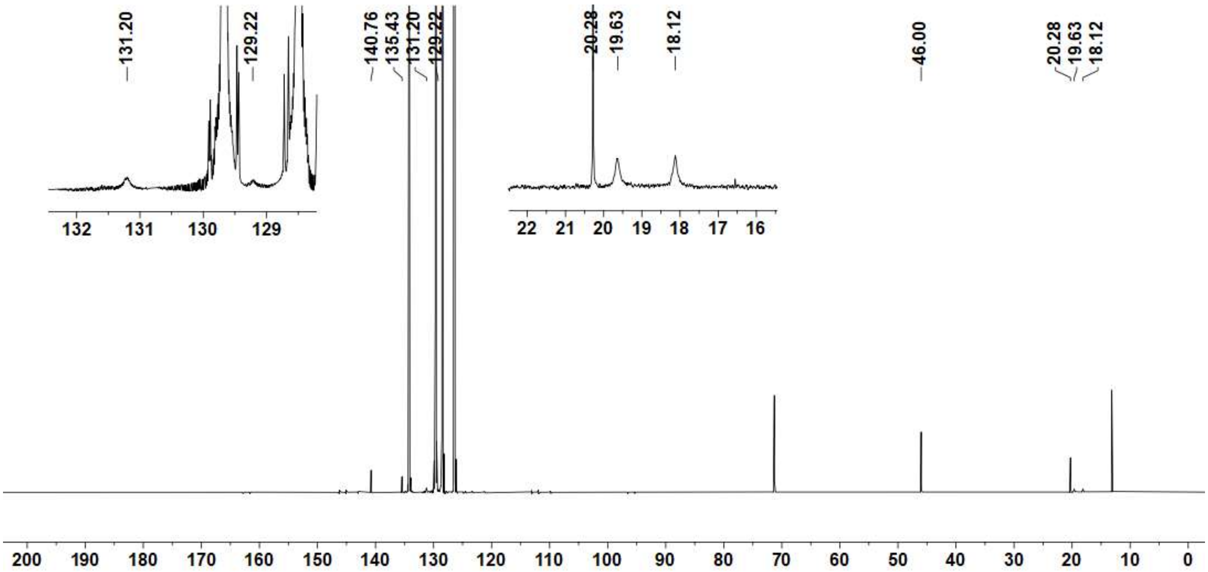

Figure S90: ^{13}C{^{1}H} NMR spectrum of (protonated *MesNMe$_2$*) in PhCl. *Note: impurity resonances at 71.28 and 13.16 ppm are due to diethylether.*

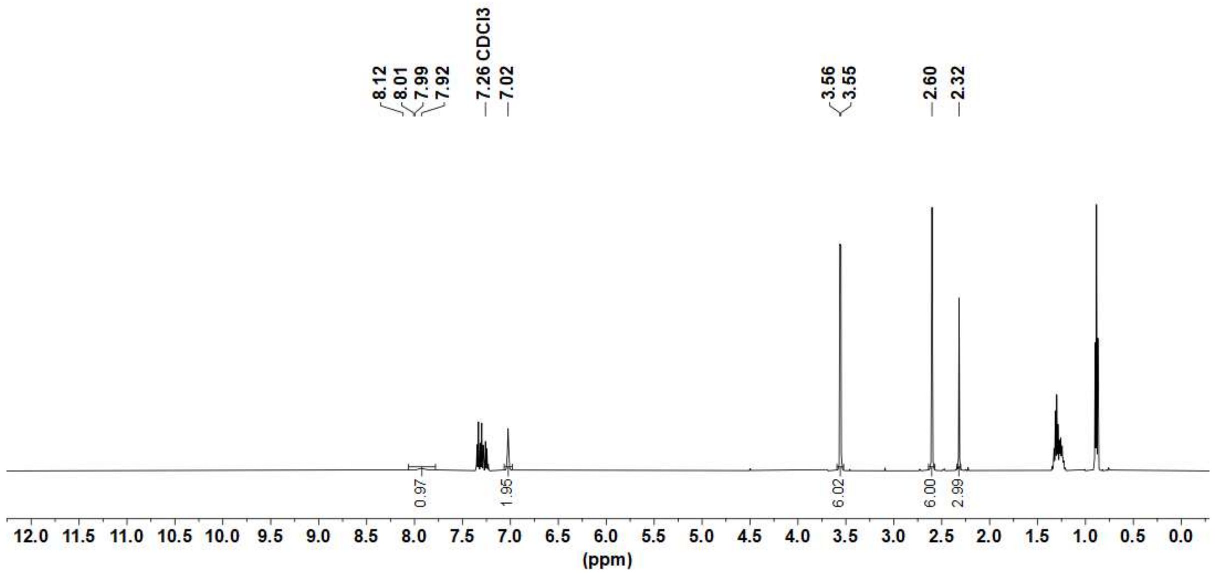

Figure S91: ^{1}H NMR spectrum of protonated *MesNMe$_2$* by-product in $CDCl_3$. *Note: Resonances at 0.8-1.3 ppm are due to pentane used to precipitate this product, trace chlorobenzene is observed at 7.30 ppm.*

10. Synthesis of C(sp^3)−H functionalised 2Br-py

10.1. Synthesis of 2-bromo-4-methyl-6-*t*Bu-pyridine (2Br-Py)

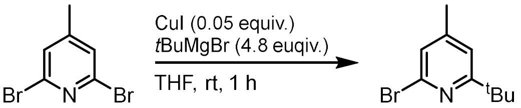

2Br-pyr

2,6-dibromopyridine (0.5 g, 2.0 mmol, 1.0 equiv.) was dissolved in THF (5 mL). CuI (0.019 g, 0.1 mmol, 0.05 equiv.) was added, followed by addition of *tert*-butyl(chloro)magnesium (1 M in THF, 9.6 mL, 9.6 mmol, 4.8 equiv.) at 0 °C. The reaction was stirred at 25 °C for 1 hr. The mixture was quenched with aq. NH_4Cl (17 mL) and extracted with ethyl acetate (3 X 17 mL). The organic phase was separated and washed once with water (17 mL), dried over Na_2SO_4 and filtered. The filtrate was concentrated *in vacuo* and purified by flash column chromatography (100% PET – 100% ethyl acetate) to give **2Br-Py** as a colourless oil in 61% yield.

^{1}H NMR (500 MHz, CDCl$_3$) δ 7.11 – 7.07 (m, 1H), 7.07 – 7.02 (m, 1H), 2.29 (s, 3H), 1.32 (s, 9H).**^{13}C{^{1}H} NMR (126 MHz, CDCl$_3$)** δ 170.85, 150.02, 141.48, 125.62, 119.15, 37.54, 30.14, 20.96.

HRMS (ESI$^+$) *m/z* calcd for $C_{10}H_{15}NBr$: 228.0382 [M+nH]$^+$, found 228.0388.

10.2. Synthesis of (13)

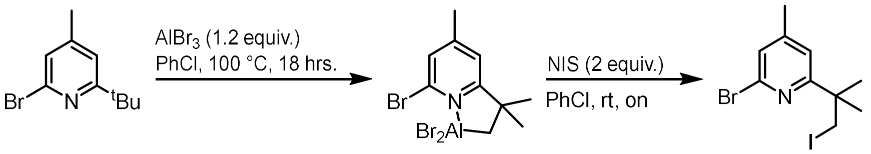

12 44% **13** 38 %

The iodination of 2-bromo-4-methyl-6-*t*Bu-pyridine (0.157 g, 0.688 mmol, 1 equiv.) proceeds via **General Procedure 1** using $AlBr_3$ (0.220 g, 0.826 mmol, 1.2 equiv.). A Young's ampule was charged with the starting material and the mixture was dissolved in PhCl (3 mL). The reaction was heated to 100 °C overnight. The aluminated compound is formed in 44% internal yield. The aluminated compound **12** was then functionalised *in situ* by addition of NIS (1.30) g, 1.376 mmol, 2 equiv.) and stirred overnight at room temperature. The remaining solid was filtered off via vacuum filtration. The organic phase was extracted using pentane (2x20 mL), washed with $Na_2S_2O_3$ (aq.) (3x20 mL) and dried over $NaSO_4$, filtrated and concentrated *in*

vacuo. The crude was purified with silica flash column chromatography (100% PET – 100% DCM) to give 38% yield (0.041 g). Note yield is based on **12**. So overall yield from both steps is 17%.

^{1}H NMR (500 MHz, $CDCl_3$) δ 7.16 (s, 1H), 7.02 (s, 1H), 3.58 (s, 2H), 2.32 (s, 3H), 1.47 (s, 6H). **^{13}C{^{1}H} NMR (126 MHz, $CDCl_3$)** δ 166.15, 150.24, 141.74, 126.50, 119.99, 41.08, 27.68, 22.64, 21.06.

HRMS (ESI$^+$) *m/z* calcd for $C_{10}H_{14}BrIN$: 353.9349 [M+H]$^+$, found 353.9342.

10.3. Synthesis of (15)

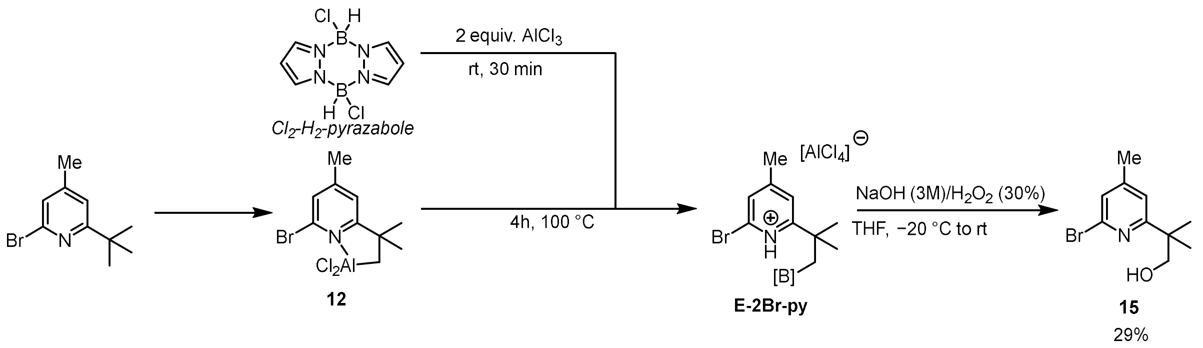

Dichloropyrazabole (0.068 g, 0.3 mmol, 1 equiv.) was added to $AlCl_3$ (0.078 g, 0.6 mmol, 2 equiv.) and stirred at room temperature for 30 minutes until all dissolved in chlorobenzene (2 mL). **E-2Br-py** from 2-bromo-4-methyl-6-*t*Bu-pyridine (0.136 g, 0.6 mmol, 2 equiv.) was synthesised according to **General Procedure 2.** The reaction mixture of $AlCl_3$ and dichloropyrazabole was transferred to the mixture containing the metalated species (**12**) and the mixture heated for 4 h at 100 °C. The reaction mixture was then diluted with THF (5 mL) and cooled to −20 °C. H_2O_2 (5 mL, 30% (aq.)) was added followed by addition of NaOH (5 mL, 3 M). The mixture was stirred at −20 °C for 1 h and then warmed to room temperature and stirred for another hour. The crude was extracted using diethylether (3x20 mL) and washed with K_2CO_3 (aq.) (3x20 mL). The combined organic phases were dried over $MgSO_4$, filtered and concentrated *in vacuo*. The product was obtained as a colourless oil in 29% yield (mass / mmol) after flash column chromatography (100% PET to 100% Ethyl acetate).

^{1}H NMR (500 MHz, $CDCl_3$) δ 7.16 (s, 1H), 7.05 (s, 1H), 3.89 (br s, 1H, <u>*OH*</u>), 3.71 (s, 2H), 2.32 (s, 3H), 1.29 (s, 6H). **^{13}C{^{1}H} NMR (126 MHz, $CDCl_3$)** 169.22, 150.93, 140.70, 126.32, 120.59, 71.77, 41.63, 25.59, 21.09.

HRMS (EI$^+$) *m/z* calcd for $C_{10}H_{13}BrNO$: 242.0175 [M−H]$^-$, found 242.0171.

10.4. Synthesis of (16)

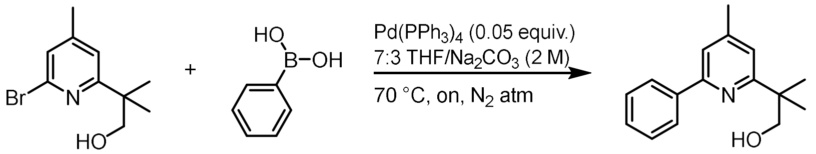

15 **16** 86%

Compound **15** (51.0 mg, 0.209 mmol, 1 equiv.) and phenylboronic acid (35.7 mg, 0.293 mmol, 1.4 equiv.) were added to a round bottomed flask and dissolved in a 7:3 (v/v) THF : 2 M aqueous Na_2CO_3 (anhydrous) solution (2.3 mL THF, 1 mL of Na_2CO_3). The solution was then degassed with nitrogen. Tetrakis(triphenylphosphine)palladium(0) (12.6 mg, 0.011 mmol, 5 mol%) was then added and the solution again degassed. The mixture then was heated to reflux (70 °C) overnight under nitrogen atmosphere. The mixture was poured into water and extracted with CH_2Cl_2.The organic phases were combined and dried over magnesium sulphate. After filtration, the CH_2Cl_2 was removed *in vacuo*. Flash column chromatography (100% PET to 100% ETA) gave colourless waxy solid in 86% yield (0.043 g / 0.089 mmol).

^{1}H NMR (500 MHz, $CDCl_3$) δ 7.96 – 7.88 (m, 2H), 7.50 – 7.43 (m, 2H), 7.43 – 7.38 (m, 2H), 7.09 – 7.06 (m, 1H), 5.48 (br s, 1H, *OH*), 3.81 (s, 2H), 2.42 (s, 3H), 1.37 (s, 6H). **^{13}C{^{1}H} NMR (126 MHz, $CDCl_3$) δ** 168.21, 155.64, 148.96, 139.37, 129.09, 128.95, 127.03, 119.83, 119.39, 72.35, 41.14, 25.95, 21.70.

HRMS (ESI$^+$) *m/z* calcd for $C_{16}H_{20}NO$: 242.1539 [M+H]$^+$, found 242.1538.

11. NMR Spectra of the C(sp^3)−H functionalised 2Br-Py

NMR Spectra of **2Br-Py**:

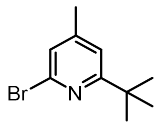

2Br-pyr

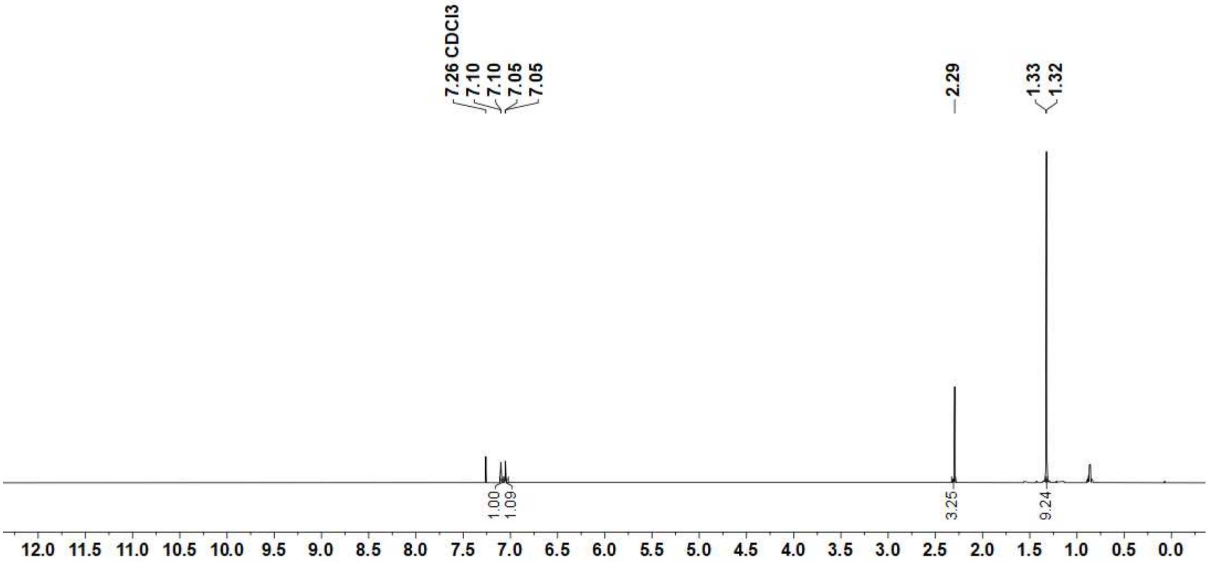

Figure S92: ^{1}H NMR spectrum of (**2Br-Py**) in $CDCl_3$.

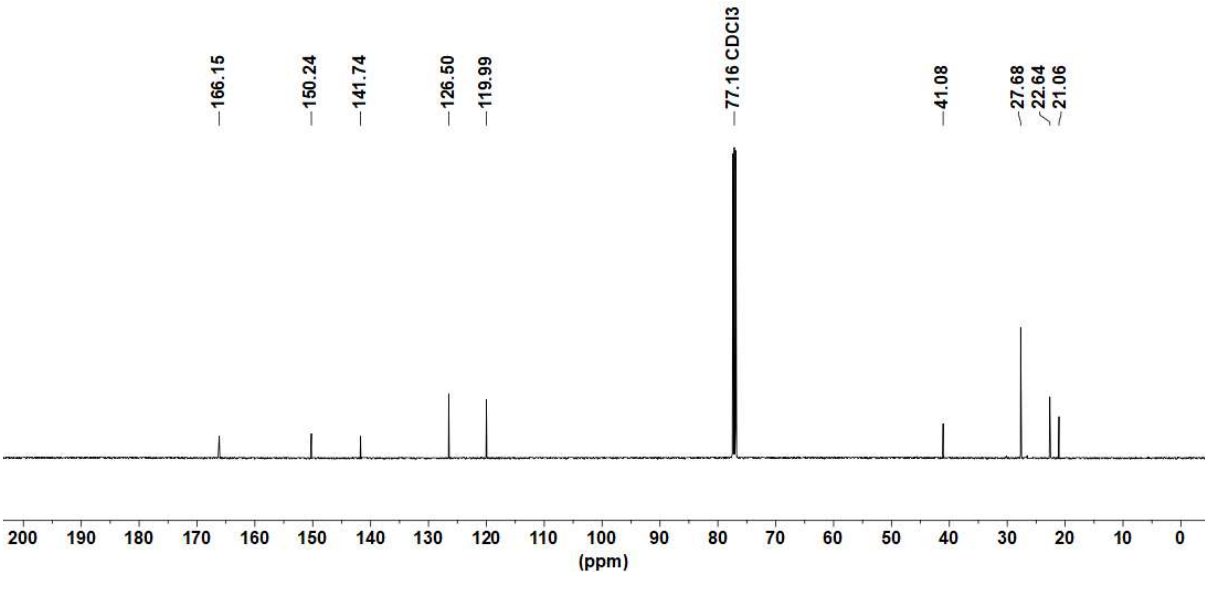

Figure S93: $^{13}C\{^1H\}$ NMR spectrum of (**2Br-Py**) in $CDCl_3$.

NMR Spectra of (**13**):

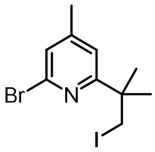

13

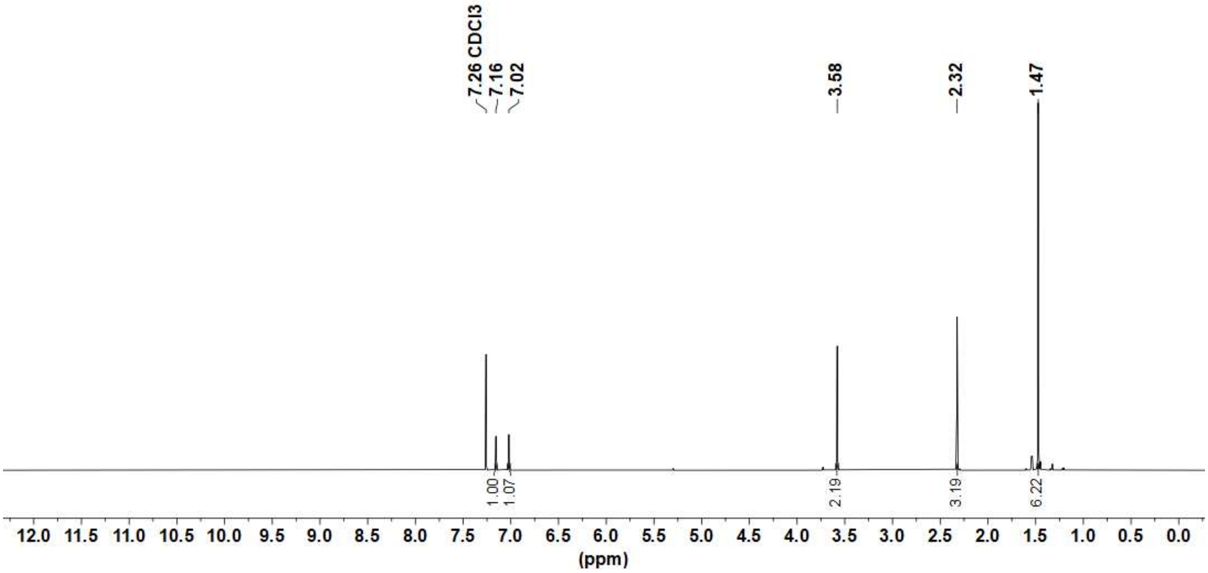

Figure S94: ^{1}H NMR spectrum of (**13**) in $CDCl_3$.

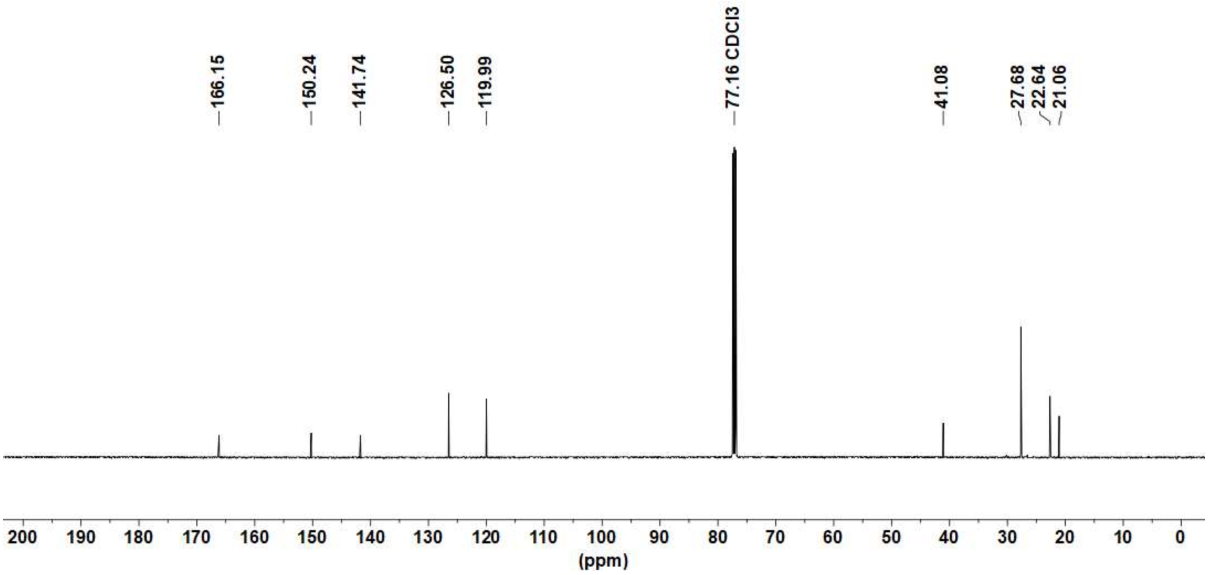

Figure S95: $^{13}C\{^{1}H\}$ NMR spectrum of (**13**) in $CDCl_3$.

NMR Spectra of (**15**):

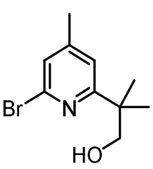

15

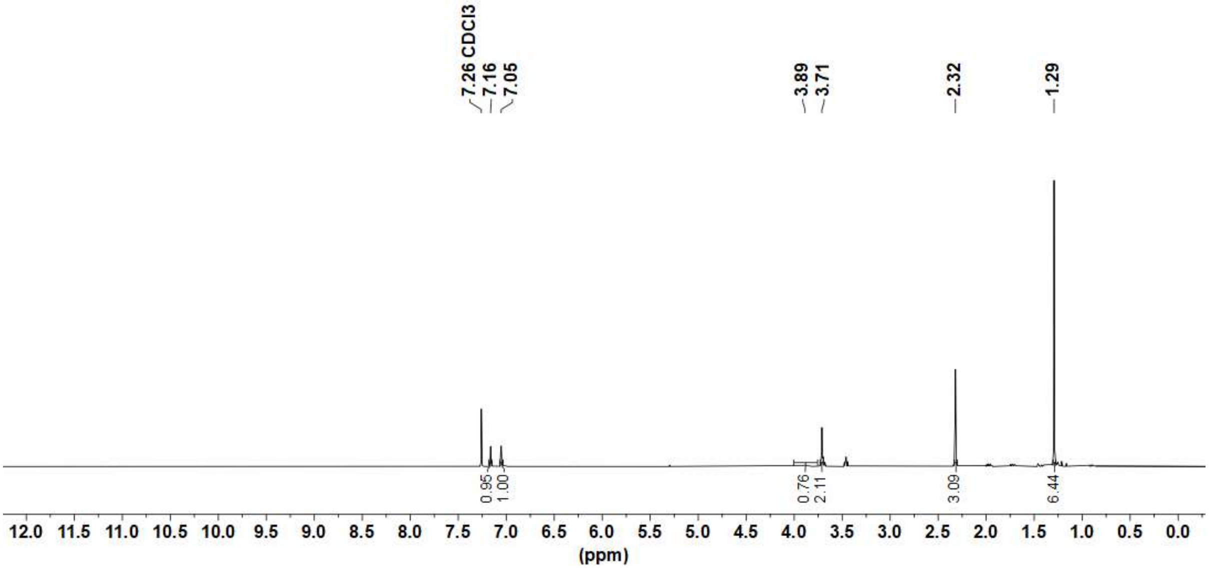

Figure S96: ^{1}H NMR spectrum of (**15**) in $CDCl_3$.

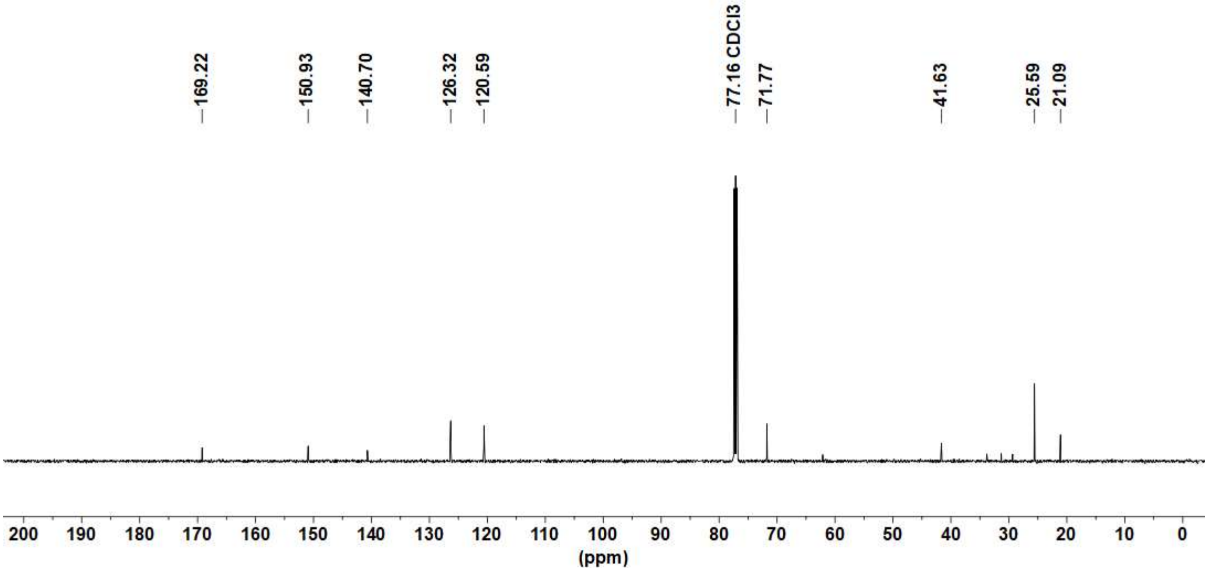

Figure S97: $^{13}C\{^{1}H\}$ NMR spectrum of (**15**) in $CDCl_3$.

<u>NMR Spectra of (**16**):</u>

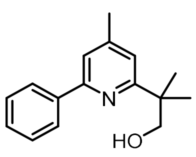

16

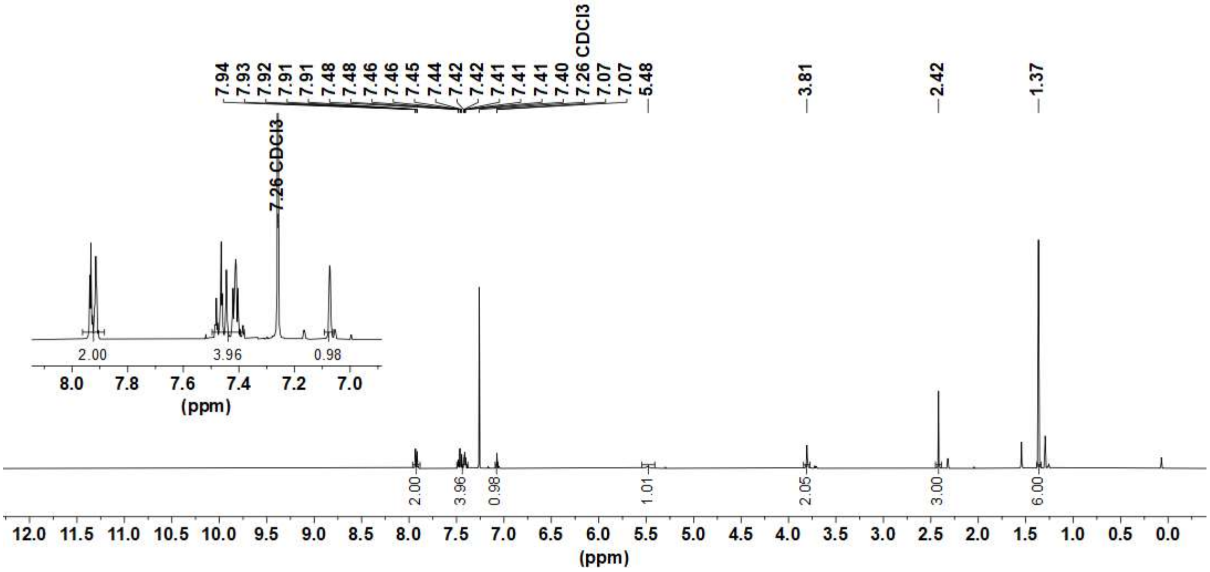

Figure S98: ^{1}H NMR spectrum of (**16**) in $CDCl_3$.

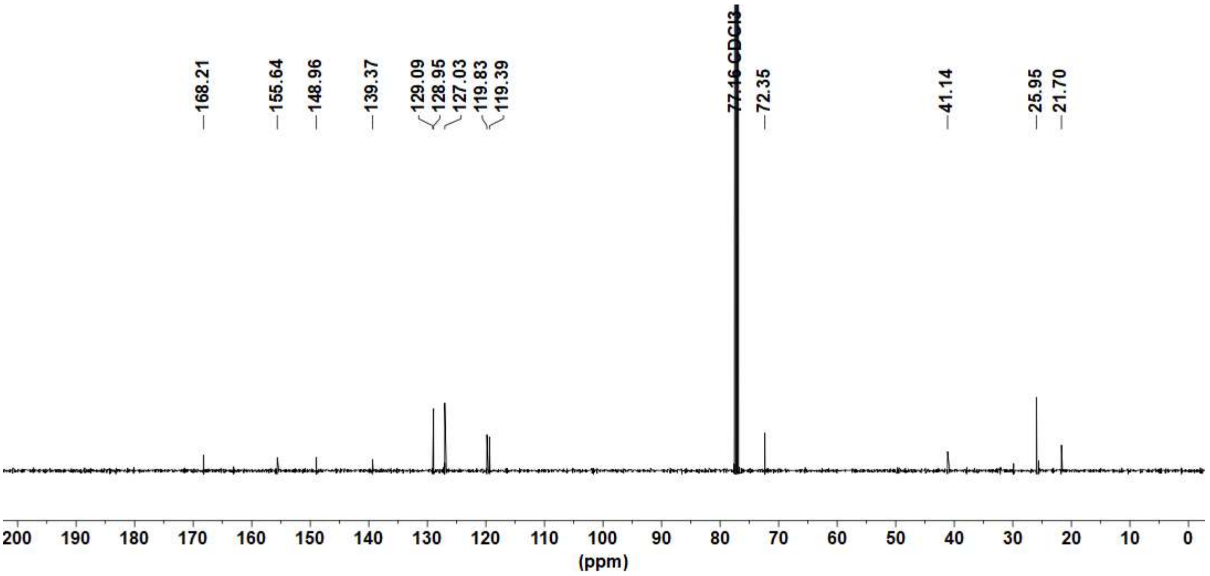

Figure S99: $^{13}C\{^{1}H\}$ NMR spectrum of (**16**) in $CDCl_3$.

12. Mechanistic Studies

11.1. Effect of 1.2 or 2.5 equiv. of MX_3 on metallation of pyridine

To a J. Young's NMR tube was added 2,6-^{t}Bu-4-methyl-pyridine or $MesNMe_2$ (1 equiv., 0.1 mmol), $GaCl_3$ or $AlCl_3$ respectively (2.5 or 1.0 equiv., 0.25 or 0.1 mmol), and chlorobenzene (1 mL). This was heated and analysed periodically by NMR spectroscopy.

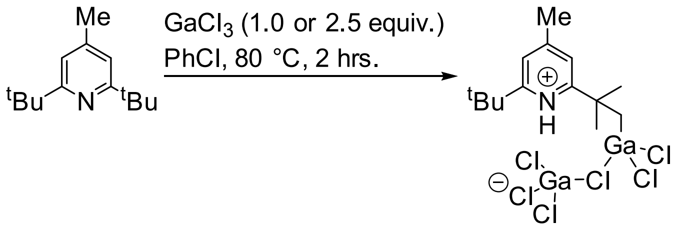

8

NMR Profiles of alumination of pyridine with $GaCl_3$ (**8**)

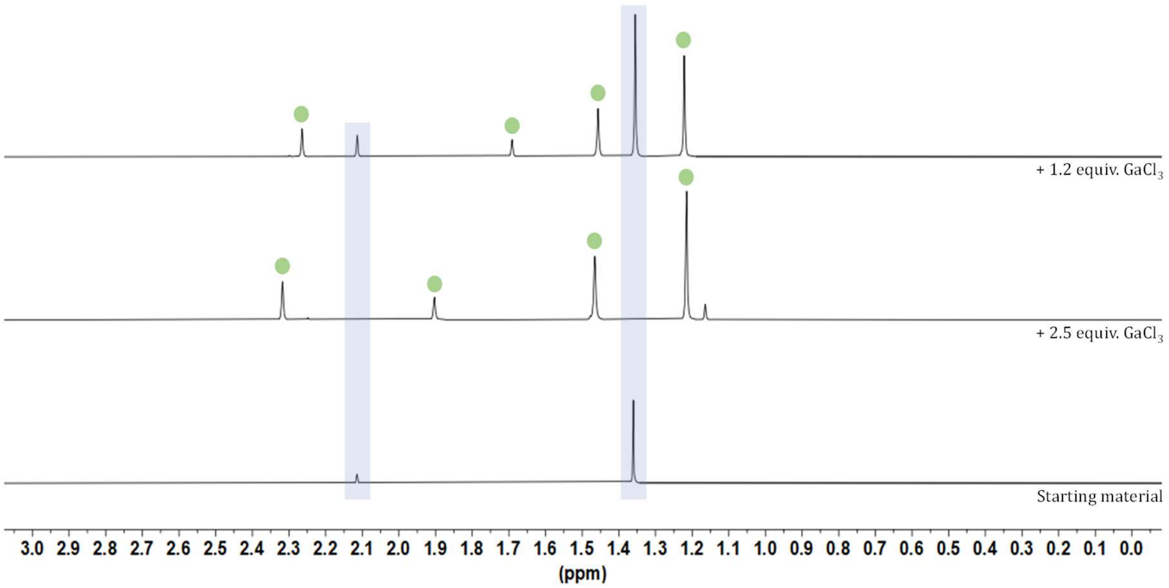

Figure S100: Stacked ^{1}H NMR spectra recorded in reaction solvent chlorobenzene after the reaction has finished. With 1.2 equiv. of $GaCl_3$ used, 45% of starting material 2,6-*t*Bu-4-methyl-pyridine is left unreacted. Peaks marked in green correspond to **8.**

11.2. Reaction of 2,6-*t*Bu-4-methyl-pyridine with 2.5 equiv. $GaCl_3$ over time

To a J. Young's NMR tube was added 2,6-^tBu-4-methyl-pyridine or $MesNMe_2$ (1 equiv., 0.1 mmol), $GaCl_3$ (2.5 equiv., 0.25 mmol), and chlorobenzene (1 mL). This was heated to 50 °C and analysed periodically by NMR spectroscopy. Note, heating to 80°C results in a rapid reaction.

<u>NMR Profiles of metallation of pyridine with $GaCl_3$ (**8**)</u>

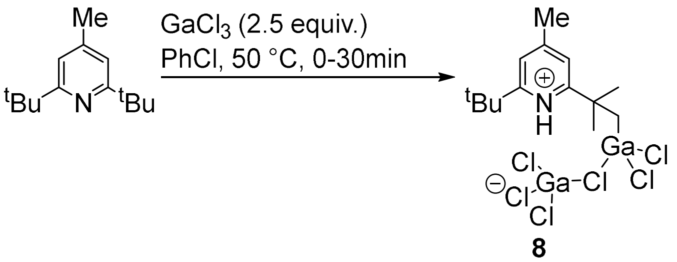

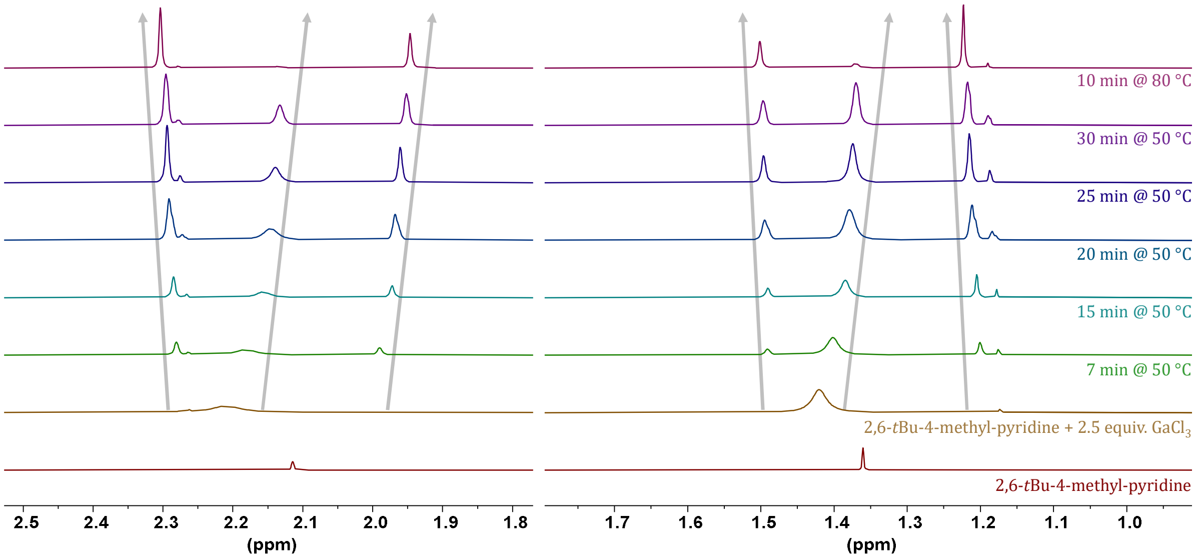

Figure S101: Stacked ^1H NMR spectra recorded in reaction solvent chlorobenzene. With 2.5 equiv. of $GaCl_3$ at specific points in time. The rise of signals associated with **8** is observed simultaneous to the broad signals corresponding to the N···Ga_2Cl_6 adduct (2.2→2.1 ppm, 1.4→1.3 ppm) sharpening and decreasing. Other minor resonances are due to trace protonated base.

11.3. *In-situ* NMR for the alumination of $MesNMe_2$

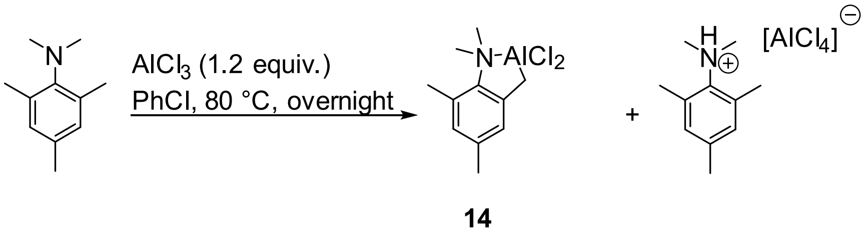

To a J. Young's NMR tube $MesNMe_2$ (1 equiv., 0.1 mmol), $AlCl_3$ (2.5 equiv., 0.25), and chlorobenzene (1 mL) were added. The reaction was heated at 80 °C overnight and analysed by NMR spectroscopy. A ca. 1:1 mixture of aluminated and protonated aniline is observed.

<u>NMR Profiles of alumination of 2,4,6-trimethyl-*N,N*-aniline with $AlCl_3$</u>

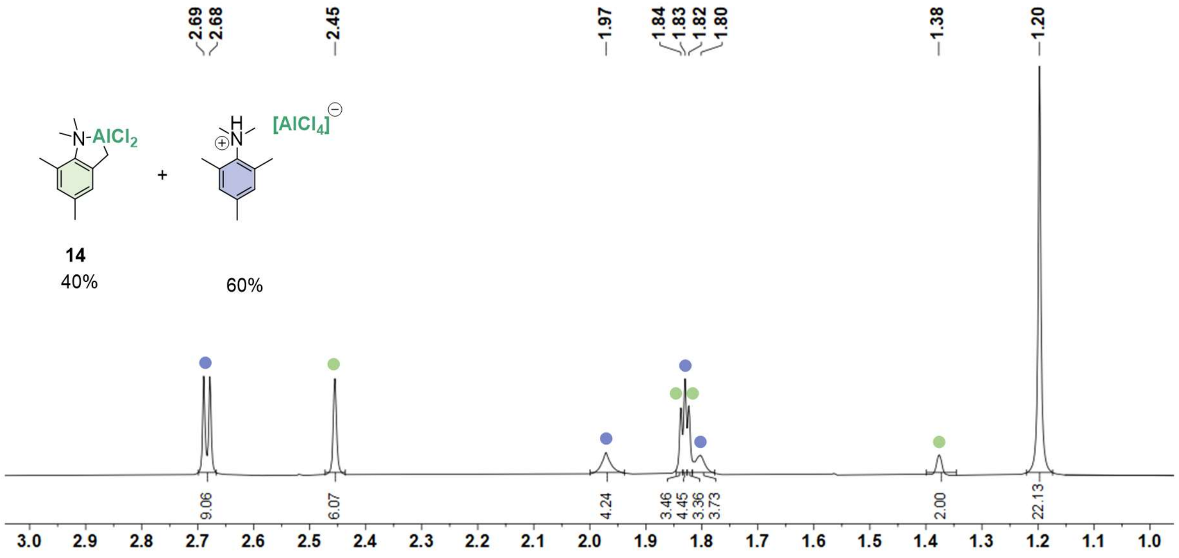

Figure S102: ^{1}H NMR spectrum of alumination of $MesNMe_2$. Spectra recorded in reaction solvent C_6H_5Cl. *Note: Signal at 1.20 ppm is due to internal standard (cyclohexane).*

Note, the $MesN(H)Me_2[AlCl_4]$ resonances are broadened at ambient temperature, thus VT NMR studies were performed.

VT NMR of product mixture from the reaction of $MesNMe_2$ with $AlCl_3$

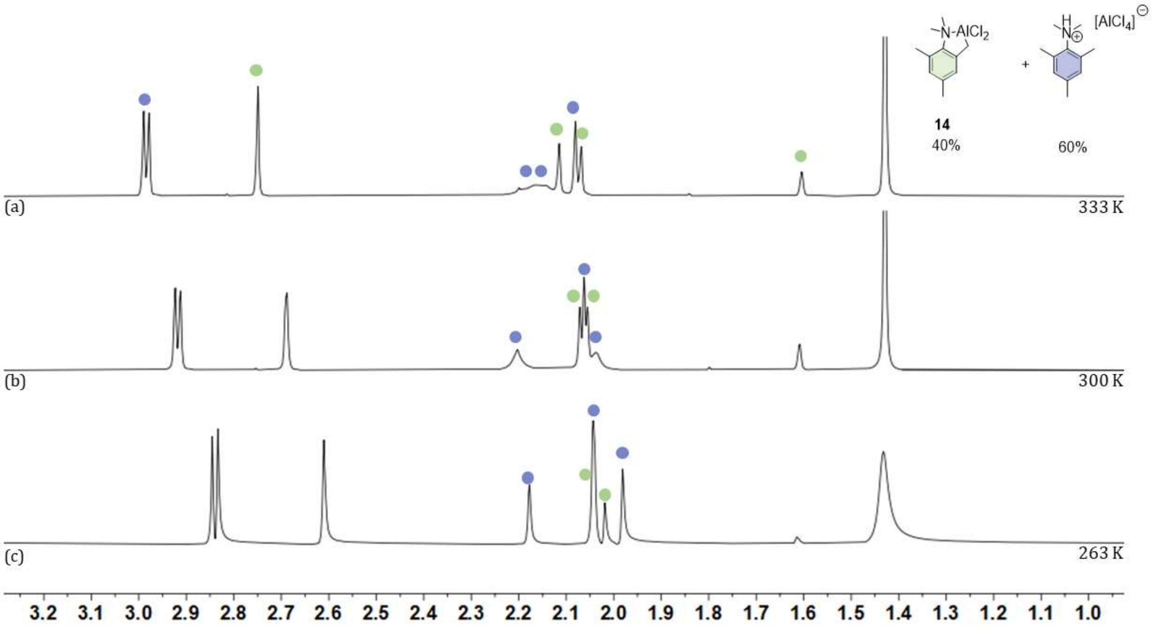

Figure S103: ^1H NMR spectra of product mixture from the reaction of $MesNMe_2$ with $AlCl_3$ at 333 K (a), 300 K (b) and 263 K (c). Spectra recorded in reaction solvent C_6H_5Cl. *Note: Resonance at 1.20 ppm is due to internal standard (cyclohexane).*

11.3. Attempt at *in-situ* alkylation of benzophenone using compound 1

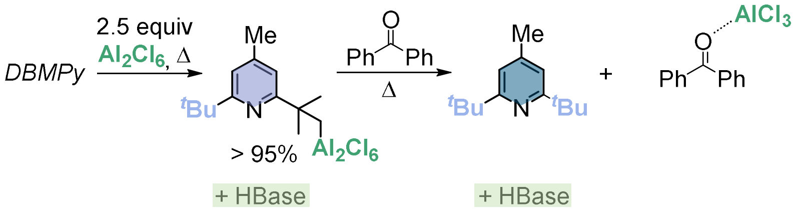

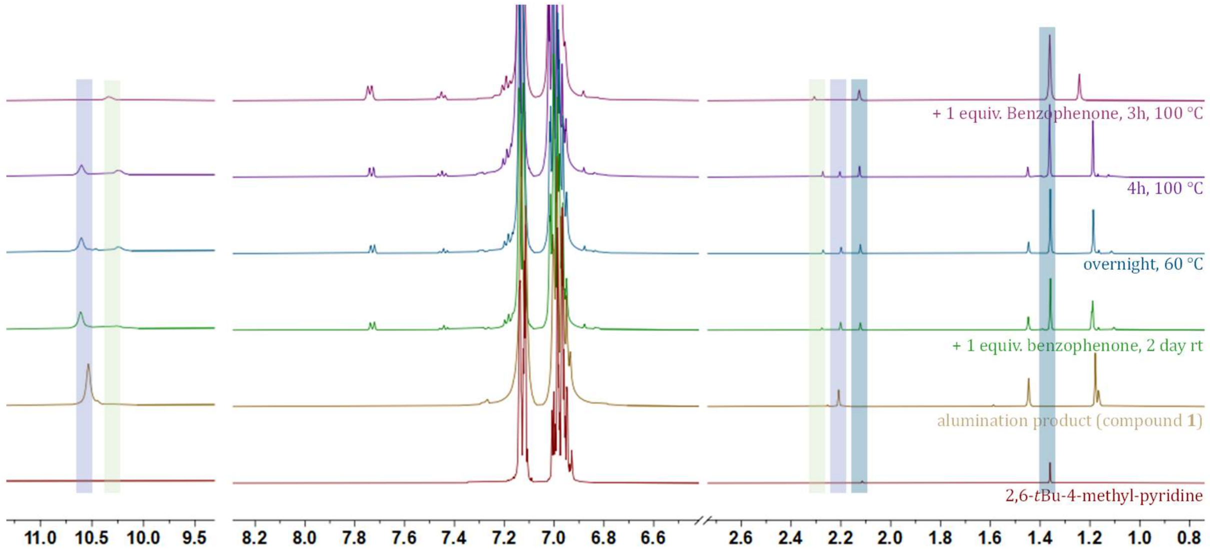

Figure S104: ^{1}H NMR spectra of alumination of 2,6-tBu-4-methylpyridine, followed by addition of benzophenone and resulting regeneration of the starting material DBMPy. Aluminated compound **1** is depicted in blue, the free base in dark blue and protonated base in green.

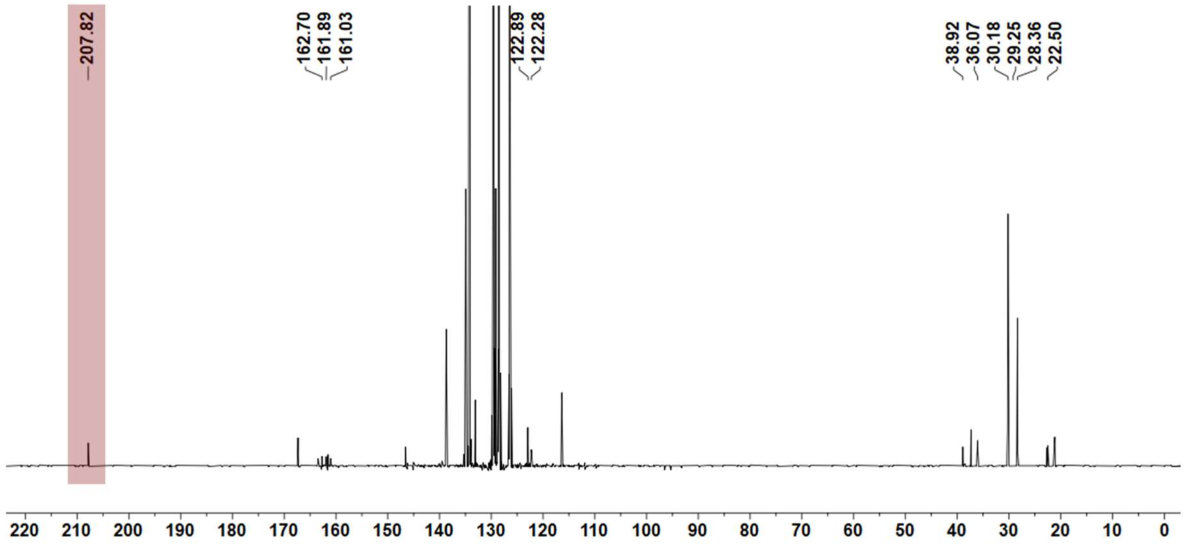

Figure S105: ^{13}C{^{1}H} NMR spectra of the reaction mixture from the addition of benzophenone to **1**. This is resulting in reaction to reform the free base. The characteristic C=O···$AlCl_3$ resonance is marked in red.

13. Computational Data

13.1. Computational Details

All calculations were performed using Gaussian 16. Conformers for each compound were generated and ranked using CREST v2.12 at the GFN2-xTB level of theory, and the lowest energy conformer taken forward for further calculations. Geometry optimisation was carried out at the PBE0-D3(BJ)/Def2-SVP level of theory and frequency calculations carried out at the same level to confirm a minima (no imaginary frequencies) or saddle point (a single imaginary frequency) and obtain thermochemical corrections. Transition state conformers were generated and ranked as previously described[3] at the PBE0-D3(BJ)/Def2-SVP level of theory. IRC calculations were used to confirm a transition state. A single-point correction was calculated at the MN15/Def2-TZVPP(SMD: chlorobenzene) level of theory and combined with the previous thermochemical corrections.

See additional supplementary file for cartesian coordinates for all calculated states in .xyz format.

14. Crystallographic Data

14.1. Crystal structure of compound 8-$GaCl_3$

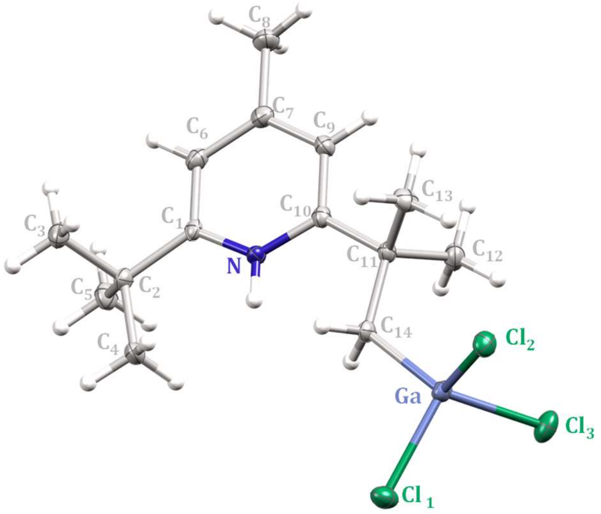

Molecular structure of **8-$GaCl_3$** in solid state (50% thermal ellipsoid probability). Selected bond distances (Å) and angles (°) for **8-$GaCl_3$**: Ga–C_{14} = 1.980(2), C_{11}-C_{14}-Ga = 120.22(15)°, C_{14}-Ga-$Cl_{2/3(eclipsed)}$ = 117.28(7)°, C_{14}-Ga-$Cl_{1(non-eclipsed)}$ = 106.14(7)

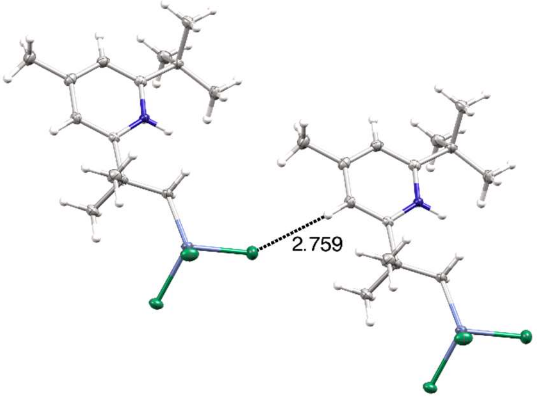

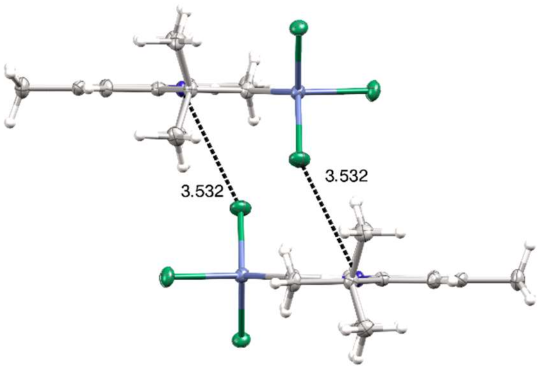

Experimental: Single colourless plate-shaped crystals of **8-$GaCl_3$** crystallised by solvent evaporation at room temperature after 2 months. A suitable crystal with dimensions 0.20 × 0.07 × 0.22 mm^3 was selected and mounted on a MITIGEN holder in Paratone oil on a XtaLAB Synergy-S diffractometer with a HyPix-Arc 100 detector. The crystal was kept at a steady T = 100.0(10) K during data collection. The structure was solved using Olex2 (Dolomanov, 2009) with Olex2.solve (Bourhis, 2015) solution program using Charge Flipping. The model was refined with the SHELXL (Sheldrick, 2015) refinement package using Least Squares minimisation on F^2.

**Compound**	**8-$GaCl_3$**
Formula	$C_{14}H_{23}Cl_3GaN$
$D_{calc.}$/ g cm^{-3}	1.451
μ/mm^{-1}	2.023
Formula Weight	381.426
Colour	colourless
Shape	plate-shaped
Size/mm^3	0.20x0.07x0.02
T/K	100.0(10)
Crystal System	triclinic
Space Group	P-1
a/Å	9.3022(4)
b/Å	9.8702(3)
c/Å	10.2146(5)
α/°	82.384(3)
β/°	71.778(4)
γ/°	79.439(3)
V/Å^3	872.89(7)
Z	2
Wavelength/Å	0.71073
Radiation type	Mo K_α
Θ_{min}/°	4.22
Θ_{max}/°	61.14
Measured Refl's.	19700
Indep't Refl's	4665
R_{int}	0.0463
Parameters	178
Restraints	0
Largest Peak	0.91
Deepest Hole	-0.37
GooF	1.014
wR_2 (all data)	0.0994
wR_2	0.0935
R_1 (all data)	0.0546
R_1	0.0383

15. References

[1] K. Niedenzu and H. Nöth, *Chem. Ber.*, 1983, **116**, 1132–1153.

[2] S. Trofimenko, *Journal of the American Chemical Society* **1967**, *89*, 3165-3170.

[3] D. R. Willcox, N. Cironis *Angewandte Chemie* **2025**, *64*, e202418495.

[4] O.V. Dolomanov, L. J. Bourhis, R. J. Gildea, J. A. K. Howard, H. Puschmann, *J. Appl. Cryst.* **2009**, 42, 339-341.

[5] L. J. Bourhis, O.V. Dolomanov, R. J. Gildea, J. A. K. Howard, H. Puschmann **2015**, *A71*, Acta Cryst., 59-75.

[6] Sheldrick, G.M. **2015**, *C71*, Acta Cryst. 3-8.
